# Mechanism Insights into the Iridium(III)- and B(C_6_F_5_)_3_-Catalyzed Reduction of CO_2_ to the Formaldehyde Level with Tertiary Silanes

**DOI:** 10.1021/acs.inorgchem.2c03330

**Published:** 2022-12-06

**Authors:** Jefferson Guzmán, Asier Urriolabeitia, Marina Padilla, Pilar García-Orduña, Víctor Polo, Francisco J. Fernández-Alvarez

**Affiliations:** † Facultad de Ciencias, Departamento de Química Inorgánica, Instituto de Síntesis Química y Catálisis Homogénea, Universidad de Zaragoza, CSIC, Zaragoza 50009, Spain; ‡ Facultad de Ciencias, Departamento de Química Física, BIFI, Universidad de Zaragoza, Zaragoza 50009, Spain

## Abstract

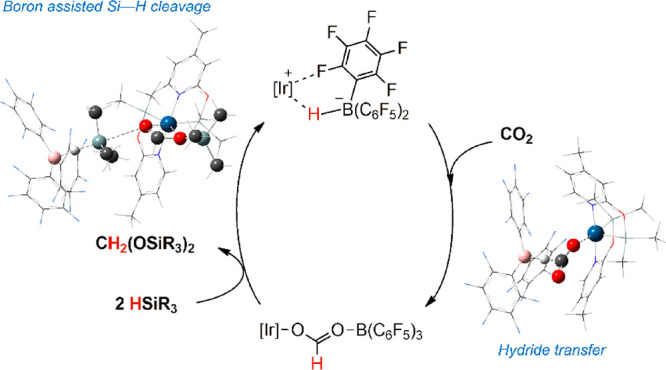

The catalytic system [Ir(CF_3_CO_2_)(κ^2^-NSi^Me^)_2_] [**1**; NSi^Me^ = (4-methylpyridin-2-yloxy)dimethylsilyl]/B(C_6_F_5_)_3_ promotes the selective reduction of CO_2_ with tertiary silanes to the corresponding bis(silyl)acetal. Stoichiometric and catalytic studies evidenced that species [Ir(CF_3_COO-B(C_6_F_5_)_3_)(κ^2^-NSi^Me^)_2_] (**3**), [Ir(κ^2^-NSi^Me^)_2_][HB(C_6_F_5_)_3_] (**4**), and [Ir(HCOO-B(C_6_F_5_)_3_)(κ^2^-NSi^Me^)_2_] (**5**) are intermediates of the catalytic process. The structure of **3** has been determined by X-ray diffraction methods. Theoretical calculations show that the rate-limiting step for the **1**/B(C_6_F_5_)_3_-catalyzed hydrosilylation of CO_2_ to bis(silyl)acetal is a boron-promoted Si–H bond cleavage via an iridium silylacetal borane adduct.

## Abstract 2

The study of the iridium(III) (κ^2^-NSi^Me^)_2_/B(C_6_F_5_)_3_-catalyzed selective reduction of CO_2_ to the formaldehyde level with tertiary silanes is reported. Mechanistic studies support a boron-assisted Si−H cleavage involving an iridium silylacetal borane adduct as the rate-limiting step of the process.

The potential of CO_2_ as a renewable and cheap C1 carbon source has received increasing attention over recent years.^1^ The major difficulties to achieve this goal are the kinetic and thermodynamic stability of CO_2_, which hampers most of its chemical transformations. In this regard, catalysis has proven to be an essential tool for transforming CO_2_ into value-added chemicals. Although great advances have been made in the field of the catalytic transformation of CO_2_, there are still many challenges to overcome for its utilization as a raw material on an industrial scale.^2,3^

Formic acid, formaldehyde, methanol, and methane are C1 chemicals that can be obtained from the reduction of CO_2_. In this work, we focus on formaldehyde, which is obtained industrially by the partial oxidation of methanol and has an annual demand of 30 million tons.^4^ The catalytic hydrogenation of CO_2_ to formaldehyde has been scarcely reported.^5^ However, several examples of the catalytic reduction of CO_2_ to the formaldehyde level with hydrosilanes^6−14^ or hydroboranes^15^ have been reported. Catalytic systems based on Zr,^6^ Re,^7^ Ru,^8^ Co,^9^ Ni,^10^ Pd,^11^ Pt,^11^ Sc,^12^ Mg,^13^ and Zn^13^ complexes and germylene-B(C_6_F_5_)_3_ adducts^14^ have proven to be effective for the selective reduction of CO_2_ with hydrosilanes to the corresponding bis(silyl)acetal. It is noteworthy that all of these catalytic systems require the use of a Lewis acid, such as B(C_6_F_5_)_3_, to selectively achieve the formation of the corresponding bis(silyl)acetal.^16^ The selectivity of these processes depends on the metal/B(C_6_F_5_)_3_ ratio. Thus, with an excess of borane, the formation of methane is facilitated.^6−14^ Although the effectivity of B(C_6_F_5_)_3_ as a hydrosilylation catalyst is well-known,^17^ B(C_6_F_5_)_3_ alone cannot catalyze the hydrosilylation of CO_2_.^6a,18^

It has recently been proven that bis(silyl)acetal, H_2_C(OSiPh_3_)_2_, provides a means to incorporate CH_*n*_ (*n* = 1 or 2) moieties into organic molecules.^19^ Therefore, developing catalytic systems effective for the reduction of CO_2_ to the bis(silyl)acetal level using hydrosilanes is of great interest.

To date, few studies have been reported on the mechanism of these processes. Indeed, the mechanistic discussion remains open. For example, two different mechanisms have been proposed for the bis(phosphino)borylnickel hydride/B(C_6_F_5_)_3_-catalyzed reduction of CO_2_ to the formaldehyde level with hydrosilanes. Thus, while Rodriguez et al. proposed a boron-promoted Si–H activation mechanism,^10a,10b^ Ke et al. proposed a nickel-promoted Si–H mechanism.^10c^

Understanding the mechanisms that operate in different transition-metal-catalyzed processes to reduce CO_2_ with hydrosiloxanes is one of our aims.^20^ We have recently reported that species [Ir(CF_3_CO_2_)(κ^2^-NSi^Me^)_2_] [**1**; NSi^Me^ = (4-methylpyridin-2-yloxy)dimethylsilyl] catalyzes the selective reduction of CO_2_ with HSiMe(OSiMe_3_)_2_ to the corresponding methoxysilane, CH_3_OSiMe(OSiMe_3_)_2_, or silylformate, HCO_2_SiMe(OSiMe_3_)_2_, under mild reaction conditions. The selectivity of this catalytic system can be easily tuned by controlling the pressure of CO_2_.^21^ It is noteworthy that the two active positions of the catalytic systems based on **1** are trans located to two silyl groups; in addition, the Ir–Si bond in such species is stronger than would be expected for a traditional Ir–silyl bond.^22^ Hence, the positions trans to the Ir–Si bonds in Ir(κ^2^-NSi^Me^)_2_ complexes are highly activated.

We now report that using **1** as a catalyst precursor in the presence of catalytic amounts of B(C_6_F_5_)_3_ allow achievement of the selective formation of bis(silyl)acetals by the reaction of CO_2_ with hydrosilanes (Scheme 1).

**Scheme 1:**
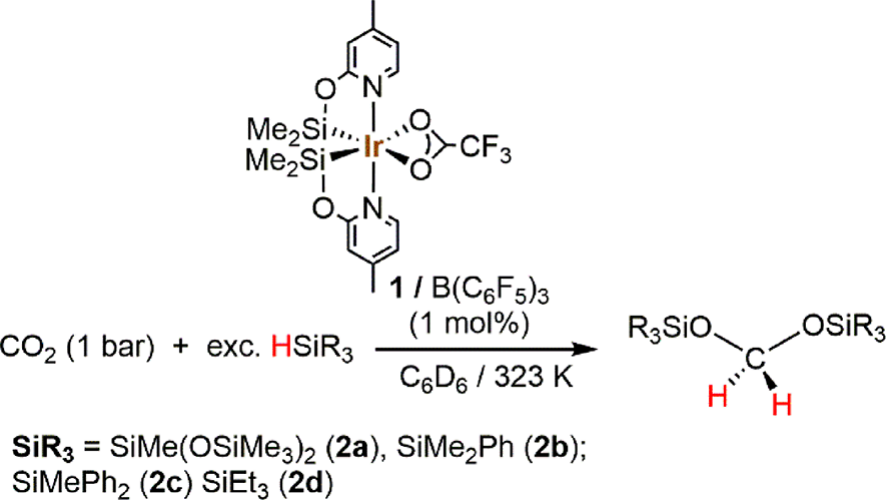
**1**-Catalyzed (1.0 mol %) Reactions of CO_2_ with Tertiary Silanes in the Presence of B(C_6_F_5_)_3_ (1.0 mol %)

^1^H NMR studies of the **1**/B(C_6_F_5_)_3_ (1:1 ratio; 1.0 mol %)-catalyzed reaction of CO_2_ (1 bar) with HSiMe(OSiMe_3_)_2_ (HMTS) in C_6_D_6_ at 323 K show the slow and selective formation of H_2_C{OSiMe(OSiMe_3_)_2_}_2_ (**2a**; Table 1, entry 1). To explore the scope of this catalytic process, we performed the reaction of CO_2_ with different silicon hydrides (HSiMe_2_Ph, HSiMePh_2_, HSiEt_3_, and HSiMe(OSiMe_3_)_2_) in the presence of **1**/B(C_6_F_5_)_3_ (1:1) in C_6_D_6_. The best reaction conditions were found to be CO_2_ (1 bar) and 323 K. The reactions are highly selective to the formation of the corresponding bis(silyl)acetal (Table 1, entries 1, 2, 4, and 5). The nature of silane influences the reaction performance. The best reaction rates were obtained using HSiMe_2_Ph and HSiMePh_2_ (Table 1). The reactions with HMTS and HSiEt_3_ were slower, which can be attributable to the higher hindrance of the Si–H bond in such compounds.

**Table 1:** Results from the **1** (1.0 mol %)- and BR_3_-Catalyzed Reaction of CO_2_ with Hydrosilanes in C_6_D_6_ at 323 K

							ratio of the reaction products			
entry	silane	borane	equiv of BR_3_	CO_2_ (bar)	time (h)	conversion (%)^a^	OCHO (%)^b^	OCH_2_O (%)^b^	CH_3_O (%)^b^	CH_4_ (%)
1	HMTS	B(C_6_F_5_)_3_	1	1	16	28		>99	<1	
					40	74		>99	<1	
2	HSiMe_2_Ph	B(C_6_F_5_)_3_	1	1	16	93		>99	<1	
					40	>99		>99		
3	HSiMe_2_Ph	BPh_3_	1	1	24	73	81	12	7	
4	HSiMePh_2_	B(C_6_F_5_)_3_	1	1	16	78		>99	<1	
					40	>99		>99	<1	
5	HSiEt_3_	B(C_6_F_5_)_3_	1	1	16	12		>99	<1	
					40	25		>99	<1	
6	HSiMe_2_Ph	B(C_6_F_5_)_3_	1	3	8	>99	83	13	4	
7	HSiMe_2_Ph	B(C_6_F_5_)_3_	2	1	24	48				>99^c^
8	HSiMe_2_Ph	B(C_6_F_5_)_3_	0.5	1	24	93	82	18		
9	HSiMe_2_Ph			1	24	50	90	2	8	

a Conversion and selectivity percentages are based on ^1^H NMR integration using hexamethylbenzene (0.0525 mmol) as an internal standard.

b Composition of the mixture of products.

c On the basis of the ^1^H NMR integral of O(SiMe_2_Ph)_2_, 12% CH_4_ was formed.

^1^H NMR studies of the **1**/B(C_6_F_5_)_3_ (1:1; 1.0 mol %)-catalyzed reaction of CO_2_ with HSiMe_2_Ph in C_6_D_6_ at 323 K demonstrate the influence of CO_2_ pressure on the reaction performance; at 3 bar, the reactions are faster but less selective than those at at 1 bar (Table 1, entries 2 and 6). The stoichiometry of borane is a key factor in the selectivity of these catalytic processes. Within the range of 1–3 bar of CO_2_, if the load of B(C_6_F_5_)_3_ is increased from 1.0 to 2.0 mol %, the reactions are selective toward the formation of methane^23^ and O(SiMe_2_Ph)_2_, albeit at a lower rate (Table 1, entry 7). While reducing the amount of B(C_6_F_5_)_3_ to 0.5 mol % does not alter the activity, the selectivity is affected, resulting in the formation of silylformate (82%) and bis(silyl)acetal (18%) as secondary products (Table 1, entry 8). In the absence of additives, the catalyst precursor **1** promotes the reduction of CO_2_ (1 bar) with HSiMe_2_Ph to give silylformate (90%) as major reaction product (Table 1, entry 9).

^1^H NMR studies of the **1**-catalyzed (1.0 mol %) reaction of CO_2_ (1 bar) with HSiMe_2_Ph in the presence of BPh_3_ (1.0 mol %), instead of B(C_6_F_5_)_3_, show a slower and less selective reaction. After 24 h, a 73% conversion of hydrosilane is reached to give a mixture of the corresponding silylformate (81%), bis(silyl)acetal (12%), and methoxysilane (7%) (Table 1, entry 3). Therefore, BPh_3_ plays a role in the activity and selectivity of the process, although to a lesser degree than B(C_6_F_5_)_3_, which can be correlated to its lower Lewis acidic character.^24^

^1^H NMR studies of the reaction of **1** with B(C_6_F_5_)_3_ evidenced the quantitative formation of [Ir(CF_3_COO-B(C_6_F_5_)_3_)(κ^2^-NSi^Me^)_2_] [**3** (CCDC 2218258); Scheme 2]. Contrarily, no reaction is observed between **1** and BPh_3_ under the same conditions. The molecular structure of **3** has been confirmed by X-ray diffraction studies (Figure S38). The geometrical parameters of the [Ir(κ^2^-NSi^Me^)_2_] fragment (see the Supporting Information, SI) agree with those of **1**, with short Ir–Si bond lengths [2.2526(11) and 2.2599(11) Å]. The Ir–O bond length in **3** [2.285(3) Å] is shorter than those found in **1** [2.363(3) and 2.418(3) Å].^22a^

**Scheme 2:**
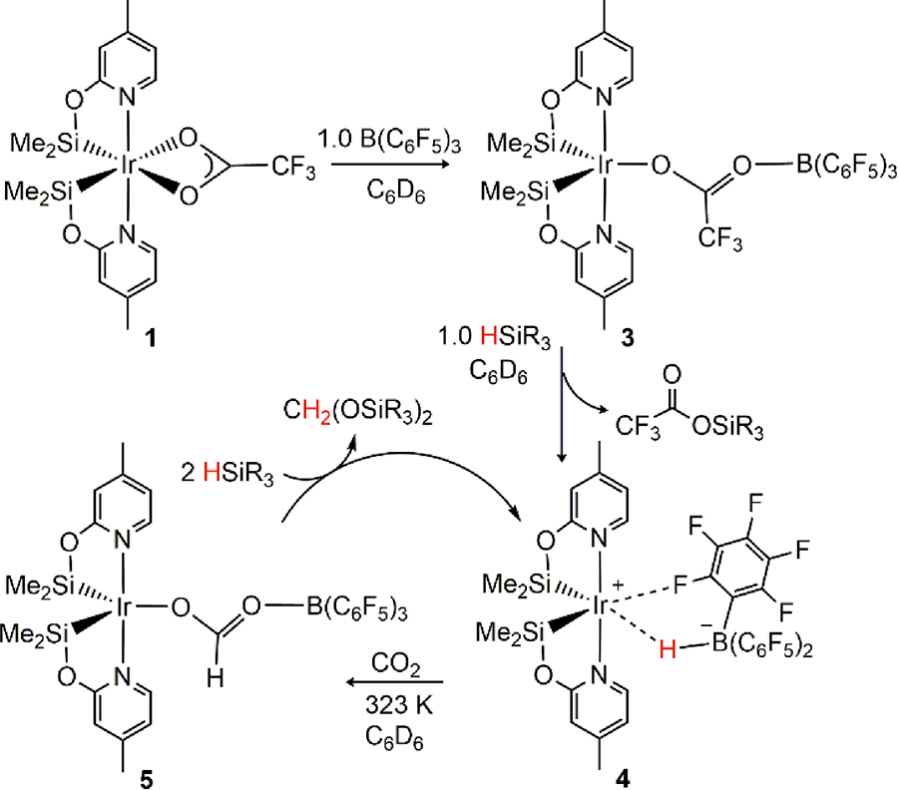
NMR Monitoring of the Stepwise Stoichiometric Reaction

The ^11^B{^1^H} NMR spectra of **3** show a singlet at δ = −1.7 ppm (Figure S18), in agreement with what is expected for the O–B(C_6_F_5_)_3_ fragment^25^ (Scheme 2). The absolute value of the difference between δ_para_ and δ_meta_ of the fluorine atoms Δ(δ_m,p_) in the ^19^F NMR spectra of **3** is 6.3 ppm (Figure S21), which agrees with the presence of a tetracoordinated borate anion.^26,27^

The addition of 1 equiv of HSiMe(OSiMe_3_)_2_, at room temperature (RT), to C_6_D_6_ solutions of **3** gives [Ir(κ^2^-NSi^Me^)_2_][HB(C_6_F_5_)_3_] (**4**) and CF_3_CO_2_SiR_3_. The ^11^B NMR spectra of **4** show a doublet resonance at δ = −15.1 ppm (^1^*J*_B–H_ = 53 Hz),^12b,15a^ which in the ^11^B{^1^H} NMR spectra appears as a singlet (Figures S24 and S25). Moreover, the ^19^F NMR spectra show a Δ(δ_m,p_) value of ∼6 ppm, which is higher than the characteristic values found for noncoordinating [HB(C_6_F_5_)_3_]^−^ anions [Δ(δ_m,p_) < 3 ppm], which indicates a certain degree of coordination of the [HB(C_6_F_5_)_3_]^−^ anion to the metallic center.^17b^

The addition of an excess of HSiMe(OSiMe_3_)_2_ to C_6_D_6_ solutions of **3** produces **4** and CF_3_CH{OSiMe(OSiMe_3_)_2_}_2_. Note that the overreduced product CF_3_CH_2_OSiMe(OSiMe_3_)_2_ is not obtained, which is reminiscent of the **1**/B(C_6_F_5_)_3_ system selectivity toward the bis(silyl)acetal species. This evidences the effective entrapment of B(C_6_F_5_)_3_ in the form of a hydridoborate ion pair because the free borane might promote activation of the Si–H bond toward reduction of the bis(silyl)acetal derivatives, as well as the direct participation of **4** in the catalytic reaction, because **4** not only promotes hydrosilylation of the TFA ligand or CO_2_ but also catalyzes reduction of the R′COOSiR_3_ species (R′ = H, CF_3_).

The ^1^H NMR spectra of **4** in C_6_D_6_ show no changes when pressurized with CO_2_ (3 bar) at RT. However, after the reaction mixture is heated at 323 K, the formation of complex [Ir(HCOOB(C_6_F_5_)_3_)(κ^2^-NSi^Me^)_2_] (**5**) is observed. The presence of a IrOC(H)OB(C_6_F_5_)_3_ moiety in **5** has been demonstrated by means of ^1^H, ^13^C, ^11^B, and ^19^F NMR spectroscopies (Figures S32–S36). The addition of 2 equiv of HSiMe(OSiMe_3_)_2_ to a solution of **5**, in the absence of CO_2_, produces the formation of **2a** and the regeneration of **4** within 1 h at RT (Scheme 2). Exposure of **5** to ^13^CO_2_ (2.7 bar) at 353 K for 48 h did not result in the partial substitution of [Ir]OC(H)OB(C_6_F_5_)_3_ to the ^13^C-enriched [Ir]O^13^C(H)OB(C_6_F_5_)_3_, which suggests that, different from that reported for analogous MOC(H)OB(C_6_F_5_)_3_ (M = Re,^8^ Ni,^10^ Pd,^11^ Pt^11^) species, the CO_2_ insertion step to give **5** is irreversible under the catalytic conditions.

Density functional theory (DFT) studies at the M06L(SMD)/def2-TZVP//B3LYP-D3(BJ)/def2-SVP level have been performed to study in detail the reaction mechanism of CO_2_ hydrosilylation catalyzed by **3** (see the SI). HSiMe_3_ has been selected as a model system for the silanes. The Gibbs free energy energetic profile for the catalyst activation process, from **3** (**A**) to **4** (**D**) (Figure S39), is exoergic by 10.8 kcal mol^–1^. Si–H bond activation occurs via boron-promoted Si–H cleavage **TSBC** (9.0 kcal mol^–1^), which corresponds to a linear S_N_2 nucleophilic attack of the terminal oxygen of the trifluoroacetate ligand to the silicon atom in which the leaving hydride is transferred to the boron moiety. A similar type of activation mechanism has been proposed for Lewis acid PBP–Ni hydrosilylation of CO_2_ based on DFT calculations.^10b^ An alternative mechanism for the Si–H activation step based on a nickel-promoted Si–H cleavage has been proposed.^10c^ In our case, the iridium-promoted Si–H cleavage is energetically disfavored (see Figures S40 and S41 for a comparison of both pathways). Intermediate **D** can be described as a hydroborate moiety and a cationic metallic complex rather than a metal hydride interacting with the Lewis acid. Inspection of the natural bond orbitals reveals a σ(B–H) bonding orbital with an electron population of 1.76 electrons (Figure S42).

The coordination of CO_2_ to **D** leads to the beginning of the catalytic cycle. The Gibbs free energy profile for this process is reported in Figure 1. The first step corresponds to hydride transfer from HB(C_6_F_5_)_3_ to CO_2_ via **TSEF** at an energy barrier of 22.2 kcal mol^–1^ from intermediate **D**. The obtained intermediate **F** is thermodynamically favored (−13.1 kcal mol^–1^) and corresponds to complex **5** experimentally detected by NMR. Following that, the addition of silane leads to σ^1^-*H*-(HSiMe_3_) coordination to **F**, yielding **G**. Then, activation of the Si–H bond takes place via **TSGH**, like the previously reported **TSBC**, consisting of the linear S_N_2 nucleophilic attack of the terminal oxygen atom of the formate to the silicon atom and transfer of the leaving hydride to the boron atom of the Lewis acid. The activation barrier of **TSGH** is 15.9 kcal mol^–1^, leading to intermediate **H**. The subsequent hydride transfer from the hydroborate to the carbon atom of the silylformate coordinated to the metal takes place through **TSHI**, yielding intermediate **I**. Upon reaction with another molecule of HSiMe_3_, the silylformate develops into the final bis(silyl)acetal product via **TSIJ**, with the activation energy for this step being 23.2 kcal mol^–1^. This activation barrier for the boron-promoted Si–H bond cleavage is higher than those of the previously related processes, **TSBC** (9.0 kcal mol^–1^) and **TSGH** (15.9 kcal mol^–1^). It should be noted that, for **TSIJ**, the nucleophilic attack to the silane is performed by an alkoxy group,^10,17c,28^ in contrast with previous steps, where the nucleophilic attack was performed by trifluoroacetate and formate groups.

**Figure 1:**
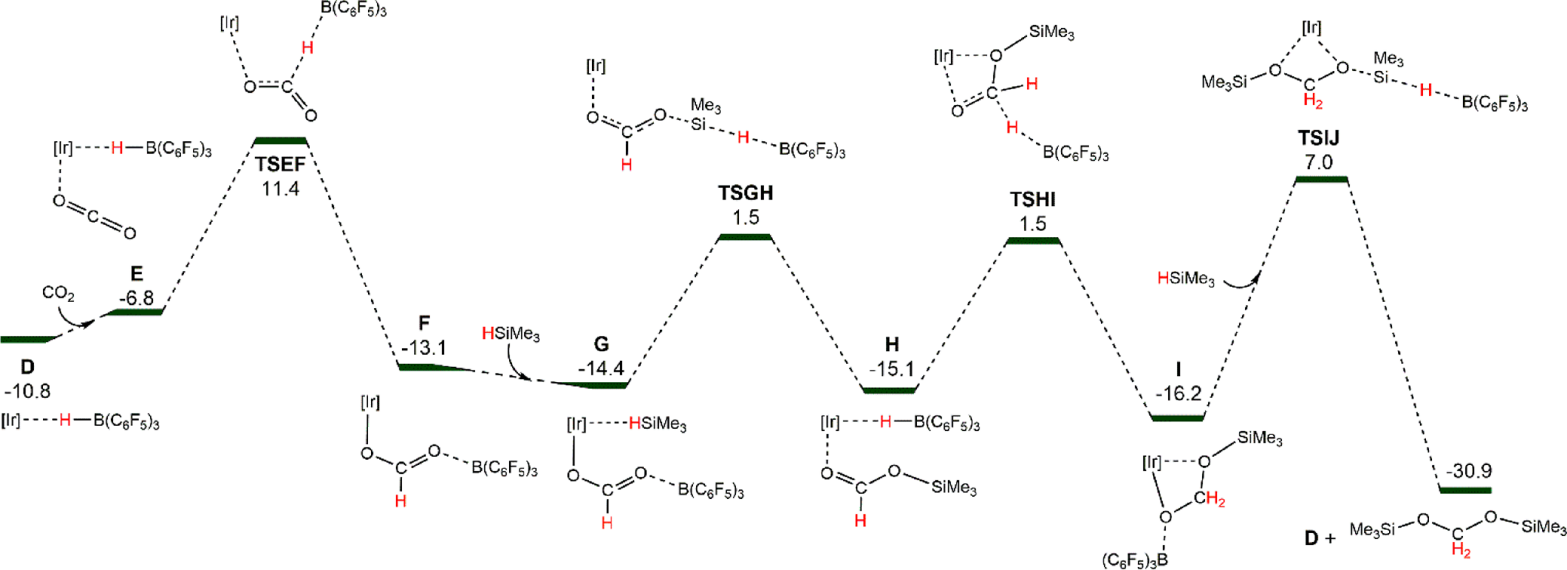
DFT-calculated Gibbs free energy profile for the catalytic formation of bis(silyl)acetal from **E** (kcal mol^–1^) relative to **A**.

The catalytic process is strongly exergonic (−30.9 kcal mol^–1^), and the rate-limiting step is boron-promoted Si–H cleavage by the iridium silylacetal borane adduct **I** (23.2 kcal mol^–1^) characterized by **TSIJ**. This activation barrier agrees with the experimental finding that the reaction proceeds slowly at RT. Indeed, the reaction of **4** with CO_2_ (3 bar) to give **5** requires heating at 323 K. It should be noted that the intermediates proposed in the DFT-calculated catalytic cycle match the experimentally detected species (**4** and **5**; Scheme 2).

In conclusion, this is the first example of an iridium-based catalytic system effective for the selective reduction of CO_2_ to the formaldehyde level with hydrosilanes. The selectivity of this catalytic system to the formation of bis(silyl)acetals is determined by the interaction between the active species and the Lewis acid B(C_5_F_6_)_3_. In fact, any factor that affects that interaction influences the selectivity of the process. Thus, using a borane with a lower Lewis acidity such as BPh_3_, high temperature, or CO_2_ pressure higher than 1.0 bar inhibit the selectivity toward the bis(silyl)acetal. DFT calculations support a boron-promoted Si–H cleavage mechanism, with the rate-limiting step being boron-promoted Si–H cleavage by the iridium silylacetal borane adduct **I**.

## Supporting Information Available

Experimental details, NMR spectra, crystallographic, and theoretical calculation data (PDF)
