# Supplementary material for: Mechanism Insights into the Iridium(III)- and B(C6F5)3-Catalyzed Reduction of CO2 to the Formaldehyde Level with Tertiary Silanes
Source: Inorg Chem. 2022 Dec 6;61(50):20216–21. doi: 10.1021/acs.inorgchem.2c03330 (PMC10468102; doi:10.1021/acs.inorgchem.2c03330)
Supplement: Supplementary file 1 — ic2c03330_si_001.pdf [file ic2c03330_si_001.pdf]

Supporting Information

Mechanism Insights into Iridium(III) and $B(C_6F_5)_3$ Catalyzed Reduction of CO_2 to Formaldehyde Level with Hydrosilanes

Jefferson Guzmán,[a] Asier Urriolabeitia,[b] Marina Padilla,[a] Víctor Polo,[b] and Francisco J. Fernández-Alvarez[a,*]

[a]Departamento de Química Inorgánica – Instituto de Síntesis Química y Catálisis Homogénea (ISQCH),
[b] Departamento de Química Fisica – BIFI,
Universidad de Zaragoza. Facultad de Ciencias 50009, Zaragoza – Spain;

1. Experimental Details 2
2. NMR Spectra 5
3. Crystal structure determination of 3 24
4. Computational Details. 26
5. References....................................71

1. Experimental Details

General information. All reactions and manipulations were carried out under an argon atmosphere by using Schlenk-type techniques or in a Glovebox-MBraun UNIlab. Organic solvents were dried by standard procedures or obtained oxygen- and water-free from a Solvent Purification System (Innovative Technologies). NMR spectra were recorded at room temperature using Bruker AV-300, AV-400, and AV-500 spectrometers. All chemical shifts (δ) are reported in ppm and coupling constants (J) are reported in Hz to apparent peak multiplicity. ^{1}H-^{1}H-COSY, ^{13}C-APT, $^{1}H/^{13}C$ HSQC, $^{1}H/^{13}C$ HMBC and $^{1}H/^{29}Si$ HMBC sequences were used for help in the assignments of the ^{1}H and $^{13}C\{^{1}H\}$ spectra.

Catalytic Reactions. In a Young cap NMR tube complex **1** (2.68 mg, 0.0042 mmol) and $B(C_6F_5)_3$ (2.15 mg, 0.0042 mmol) were dissolved in C_6D_6 (0.4 mL). To this solution, 0.42 mmol of hydrosilane was added ($HSiMe(OSiMe_3)_2$, 114 μL; $HSiEt_3$, 67.0 μL; $HSiMe_2Ph$, 64.4 μL; $HSiMePh_2$, 83.7 μL). The resulting solution was degassed by freeze-pump-thaw procedure, and then the tube was filled with CO_2 (1 bar) and heated if desired.

Catalytic Reactions in presence of an internal standard. In a Young cap NMR tube complex **1** (2.68 mg, 0.0042 mmol), $B(C_6F_5)_3$ (2.15 mg, 0.0042 mmol) and hexamenthylbenzene (8.5 mg, 0.0525 mmol) were dissolved in C_6D_6 (0.4 mL). To this solution, 0.42 mmol of hydrosilane was added ($HSiMe(OSiMe_3)_2$, 114 μL; $HSiEt_3$, 67.0 μL; $HSiMe_2Ph$, 64.4 μL; $HSiMePh_2$, 83.7 μL). The resulting solution was degassed by freeze-pump-thaw procedure, and then the tube was filled with CO_2 (1 bar) and heated at 323 K. The tubes were monitored by ^{1}H NMR spectroscopy after 16 and 40 hours. The results are shown in Table 1.

Stoichiometric Reactions. In a Young cap NMR tube complex **1** (20 mg, 0.031 mmol) and $B(C_6F_5)_3$ (16.1 mg, 0.031 mmol) were dissolved in C_6D_6 (0.4 mL). To this solution, $HSiMe(OSiMe_3)_2$ (8.5 μL, 0.031 mmol or 85 μL, 0.31 mmol) was added. The resulting solution was degassed by freeze-pump-thaw procedure, and then the tube was filled with CO_2 (1 or 3 bar) and heated if desired.

$CH_2\{OSiMe(OSiMe_3)_2\}_2$ (2a): ^{1}H NMR (300 MHz, 298 K, C_6D_6): *δ* 5.28 (s, 2H, CH_2), 0.23 (s, 6H, $SiCH_3$), 0.19 (s, 36H, $Si(CH_3)_3$). ^{13}C APT (75 MHz, 298 K, C_6D_6): *δ* 82.8 (s,

CH_2), 1.8 (s, Si-$(CH_3)_3$), -2.6 (s, $SiCH_3$). ^1H-^{29}Si HMBC (60 MHz, 298 K, C_6D_6): δ 8.2 (s, Si-$(CH_3)_3$), -57.6 (s, $SiOCH_2$).

$CH_2(OSiMe_2Ph)_2$ (2b): ^1H NMR (300 MHz, 298 K, C_6D_6): δ 7.59-7.54 (m, 4H, Ph), 7.24-7.17 (m, 6H, Ph), 5.06 (s, 2H, CH_2), 0.35 (s, 12H, $SiCH_3$). ^{13}C APT (75 MHz, 298 K, C_6D_6): δ 138.0 (s, C_{ipso}), 133.9 (s, Ph), 129.9 (s, Ph), 128.2 (s, Ph), 84.9 (s, CH_2), -1.1 (s, $SiCH_3$). ^1H-^{29}Si HMBC (60 MHz, 298 K, C_6D_6): δ 7.0 (s, $SiOCH_2$).

$CH_2(OSiMePh_2)_2$ (2c): ^1H NMR (300 MHz, 298 K, C_6D_6): δ 7.64-7.60 (m, 8H, Ph), 7.20-7.17 (m, 12H, Ph), 5.23 (s, 2H, CH_2), 0.61 (s, 6H, $SiCH_3$). ^{13}C APT (75 MHz, 298 K, C_6D_6): δ 136.3 (s, C_{ipso}), 134.9 (s, Ph), 130.1 (s, Ph), 128.2 (s, Ph), 85.4 (s, CH_2), -2.2 (s, $SiCH_3$). ^1H-^{29}Si HMBC (60 MHz, 298 K, C_6D_6): δ -2.6 (s, $SiOCH_2$).

$CH_2(OSiEt_3)_2$ (2d): ^1H NMR (300 MHz, 298 K, C_6D_6): δ 5.05 (s, 2H, OCH_2), 1.02 (t, 18H, CH_3), 0.64 (q, 12H, $SiCH_2$). ^{13}C APT (75 MHz, 298 K, C_6D_6): δ 84.6 (s, OCH_2), 7.0 (s, CH_3), 5.3 (s, $SiCH_2$). ^1H-^{29}Si HMBC (60 MHz, 298 K, C_6D_6): δ 18.6 (s, $SiOCH_2$).

[Ir(CF_3COO-B$(C_6F_5)_3$)(κ^2-NSi^{Me})$_2$] (3): ^1H NMR (400 MHz, 298 K, C_6D_6): δ 7.46 (d, 2H, $^3J_{H\text{-}H}$ = 5.9 Hz, py), 6.29 (s, 2H, py), 6.17 (d, 2H, $^3J_{H\text{-}H}$ = 5.9 Hz, py), 1.62 (s, 6H, py-CH_3), 0.46 (s, 6H, Si-CH_3), 0.22 (s, 6H, Si-CH_3). ^{11}B NMR (128 MHz, 298 K, C_6D_6): δ -1.7 (br s, C=O···B(C_6F_5)). ^{13}C APT (100 MHz, 298 K, C_6D_6): δ 168.0 (s, py-C_{ipso}), 163.2 (q, $^2J_{C\text{-}F}$ = 39.6 Hz, $CF_3\mathit{C}O_2$), 154.6 (s, py-C_{ipso}), 148.6 (dm, $^2J_{C\text{-}F}$ = 241 Hz, *orto*-C_6F_5), 145.9 (s, py), 140.1 (dm, $^2J_{C\text{-}F}$ = 248 Hz, *para*-C_6F_5), 137.4 (dm, $^2J_{C\text{-}F}$ = 248 Hz, *meta*-C_6F_5), 119.2 (s, py), 115.6 (q, $^2J_{C\text{-}F}$ = 287.6 Hz, $\mathit{C}F_3CO_2$), 112.6 (s, py), 20.7 (s, py-CH_3), 3.7 (s, Si-CH_3), 2.0 (s, Si-CH_3). ^{19}F NMR (376 MHz, 298 K, C_6D_6): δ -75.0 (s, 1F, CF_3CO_2), -134.2 (d, 6F, $^3J_{F\text{-}F}$ = 21.1 Hz, *orto*-$C_6\mathit{F}_5$), -158.3 (t, 3F, $^3J_{F\text{-}F}$ = 20.1 Hz, *para*-$C_6\mathit{F}_5$), -164.6 (t, 6F, $^3J_{F\text{-}F}$ = 19.0 Hz, *meta*-$C_6\mathit{F}_5$). ^1H-^{29}Si HMBC (80 MHz, 298 K, C_6D_6): δ 40.4 (s, Ir-Si).

[Ir(κ^2-NSi^{Me})$_2$][HB$(C_6F_5)_3$] (4): ^1H NMR (400 MHz, 298 K, C_6D_6): δ 7.52 (d, 2H, $^3J_{H\text{-}H}$ = 6.3 Hz, py), 6.24 (s, 2H, py), 5.55 (d, 2H, $^3J_{H\text{-}H}$ = 6.3 Hz, py), 4.22 (ps q, 1H, $^1J_{H\text{-}B}$ = 57 Hz, HB$(C_6F_5)_3$), 1.53 (s, 6H, py-CH_3), 0.62 (s, 6H, Si-CH_3), 0.27 (s, 6H, Si-CH_3). ^{11}B NMR (128 MHz, 298 K, C_6D_6): δ -15.1 (d, $^1J_{B\text{-}H}$ = 57 Hz, HB$(C_6F_5)_3$). ^{13}C APT (101 MHz, 298 K, C_6D_6): δ 168.1 (s, py-C_{ipso}), 153.8 (s, py-C_{ipso}), 148.9 (dm, $^2J_{C\text{-}F}$ = 244 Hz, *orto*-C_6F_5), 148.1 (s, py), 139.9 (dm, $^2J_{C\text{-}F}$ = 241 Hz, *para*-C_6F_5), 137.6 (dm, $^2J_{C\text{-}F}$ = 239 Hz, *meta*-C_6F_5), 117.9 (s, py), 112.3 (s, py), 20.5 (s, py-CH_3), 3.8 (s, Si-CH_3), 2.1 (s, Si-

CH_3). ^{19}F NMR (376 MHz, 298 K, C_6D_6): δ -132.5 (d, 6F, $^3J_{F-F}$ = 21.6 Hz, *orto*-C_6F_5), -159.2 (br s, 3F, *para*-C_6F_5), -164.1 (br s, 6F, *meta*-C_6F_5). ^1H-^{29}Si HMBC (79 MHz, 298 K, C_6D_6): δ 40.0 (s, Ir-Si).

[Ir(HCOO-B(C_6F_5)$_3$)(κ^2-NSi^{Me})$_2$] (5): ^1H NMR (300 MHz, 298 K, C_6D_6): δ 8.80 (s, 1H, Ir-OCHO), 7.87 (d, 2H, $^3J_{H-H}$ = 5.9 Hz, py), 6.32 (s, 2H, py), 6.11 (d, 2H, $^3J_{H-H}$ = 5.9 Hz, py), 1.59 (s, 6H, py-CH_3), 0.57 (s, 6H, Si-CH_3), 0.37 (s, 6H, Si-CH_3). ^{11}B NMR (96 MHz, 298 K, C_6D_6): δ -1.2 (br s, C=O···B(C_6F_5)). ^{13}C APT (75 MHz, 298 K, C_6D_6): δ 173.1 (s, Ir-OCHO), 168.3 (s, py-C_{ipso}), 153.7 (s, py-C_{ipso}), 148.6 (dm, $^2J_{C-F}$ = 237 Hz, *orto*-C_6F_5), 147.2 (s, py), 140.1 (dm, $^2J_{C-F}$ = 243 Hz, *para*-C_6F_5), 137.5 (dm, $^2J_{C-F}$ = 240 Hz, *meta*-C_6F_5), 118.7 (s, py), 112.3 (s, py), 20.6 (s, py-CH_3), 4.0 (s, Si-CH_3), 2.1 (s, Si-CH_3). ^{19}F NMR (282 MHz, 298 K, C_6D_6): δ -133.6 (d, 6F, $^3J_{F-F}$ = 23.3 Hz, *orto*-C_6F_5), -157.9 (t, 3F, $^3J_{F-F}$ = 20.6 Hz, *para*-C_6F_5), -164.0 (m, 6F, *meta*-C_6F_5). ^1H-^{29}Si HMBC (60 MHz, 298 K, C_6D_6): δ 42.1 (s, Ir-Si).

$CF_3CH\{OSiMe(OSiMe_3)_2\}_2$: ^1H NMR (400 MHz, 298 K, C_6D_6): δ 5.69 (q, 1H, $^3J_{H-F}$ = 3.5 Hz, CF_3CH) . ^{13}C APT (101 MHz, 298 K, C_6D_6): δ 126.6 (q, $^1J_{C-F}$ = 286 Hz, CF_3), 86.8 (q, $^2J_{C-F}$ = 38.0 Hz, CH). ^{19}F NMR (376 MHz, 298 K, C_6D_6): δ -84.6 (d, $^3J_{H-F}$ = 3.5 Hz, CF_3CH). ^1H-^{29}Si HMBC (79 MHz, 298 K, C_6D_6): δ 9.86 (d, J_{Si-F} = 7.9 Hz, $OSiMe_3$), -58.5 (s, CF_3CH-OSi).

2. NMR Spectra

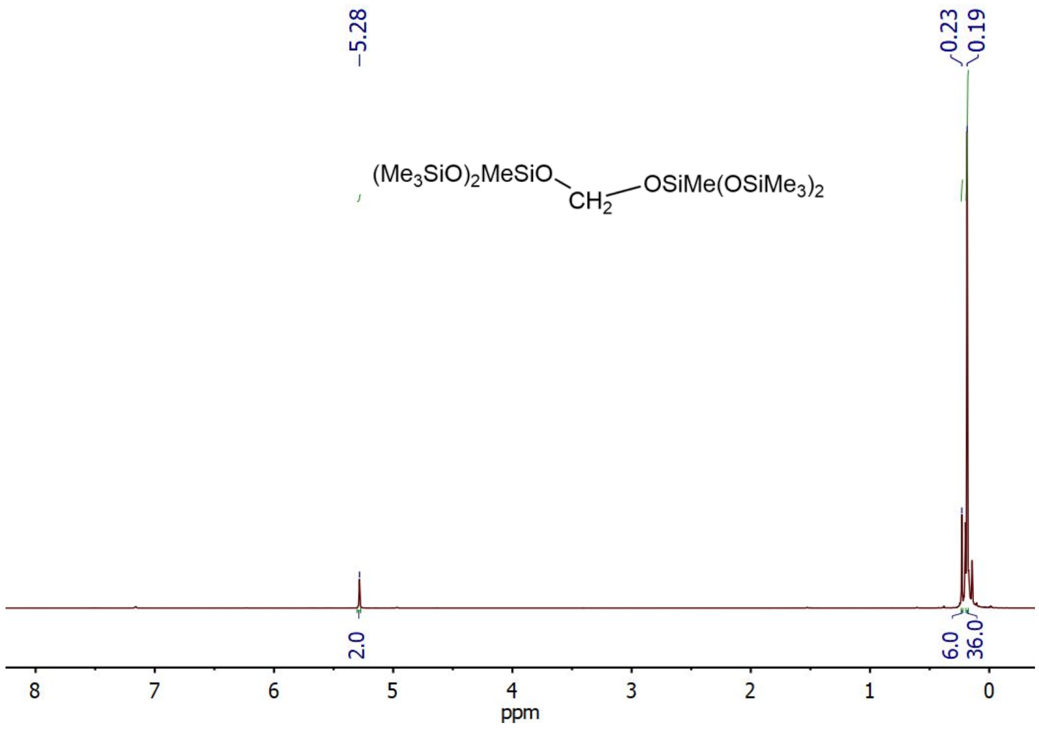

Figure S1. ^{1}H NMR spectrum of **2a** in C_6D_6.

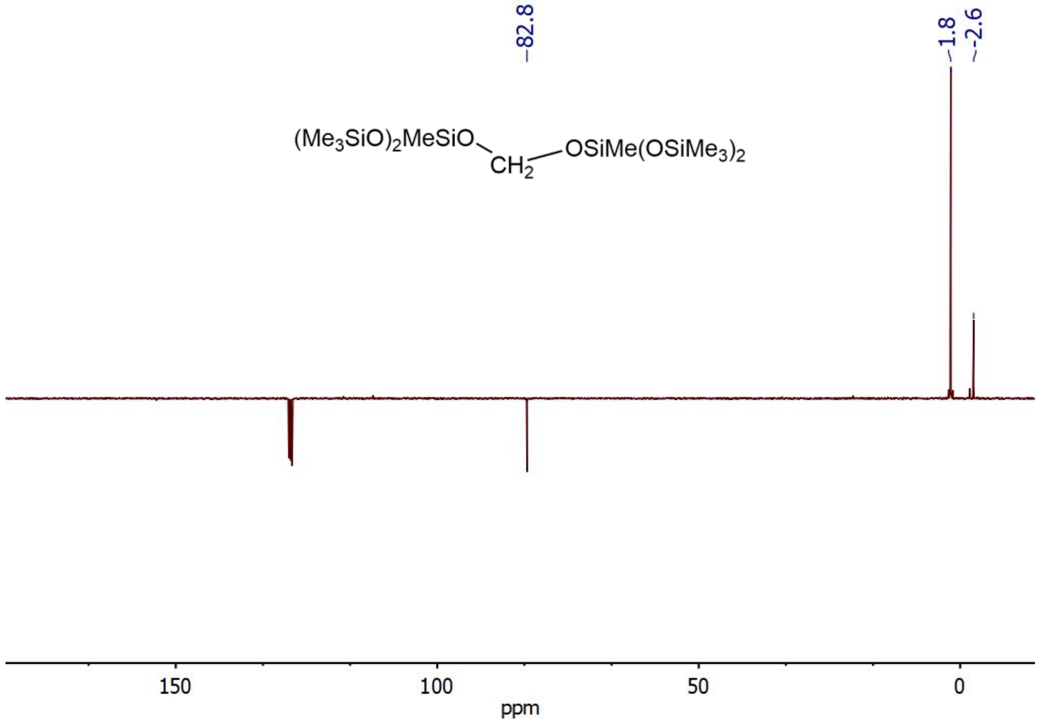

Figure S2. ^{13}C APT NMR spectrum of **2a** in C_6D_6.

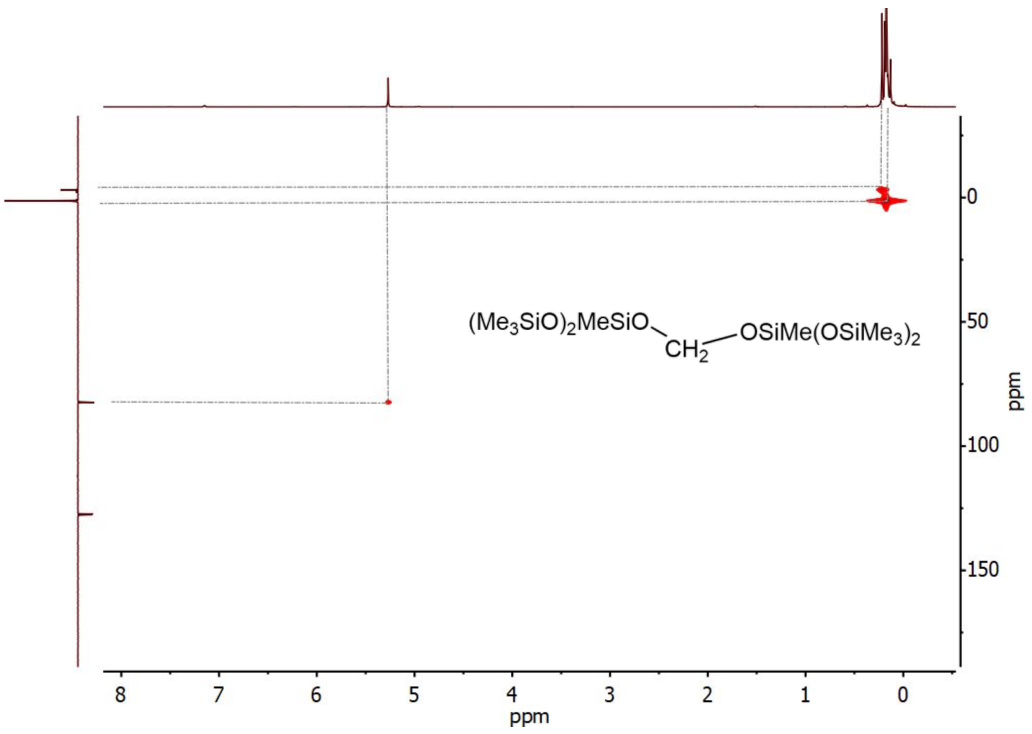

Figure S3. ^{1}H-^{13}C HSQC NMR spectrum of **2a** in C_6D_6.

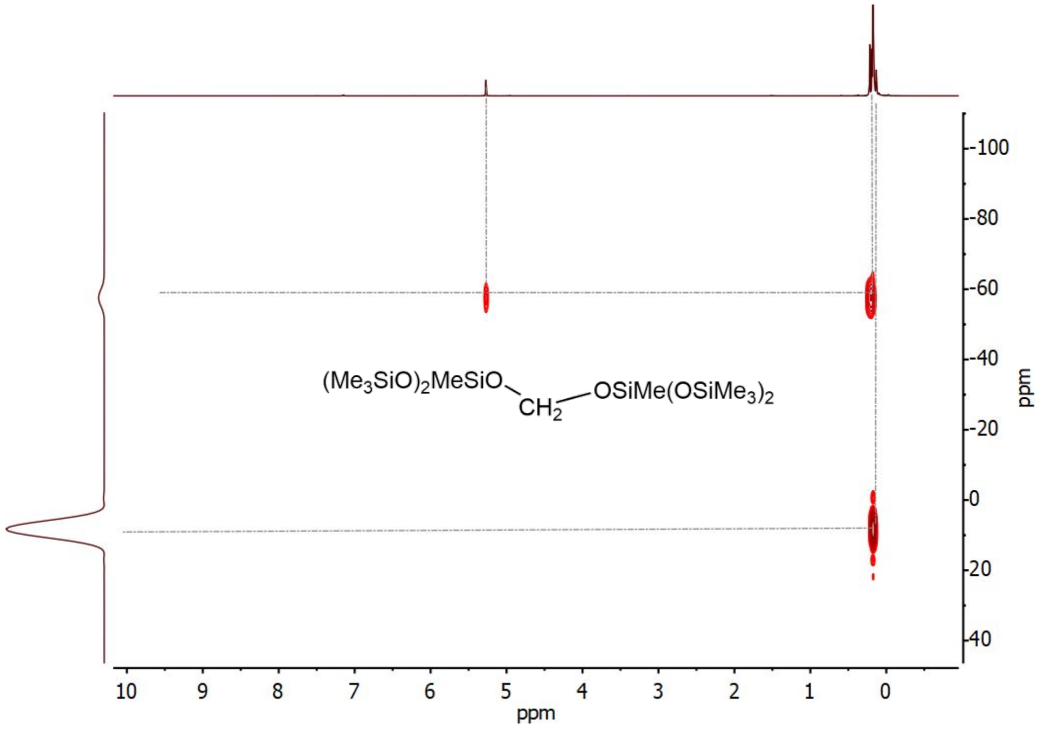

Figure S4. ^{1}H-^{29}Si HMBC NMR spectrum of **2a** in C_6D_6.

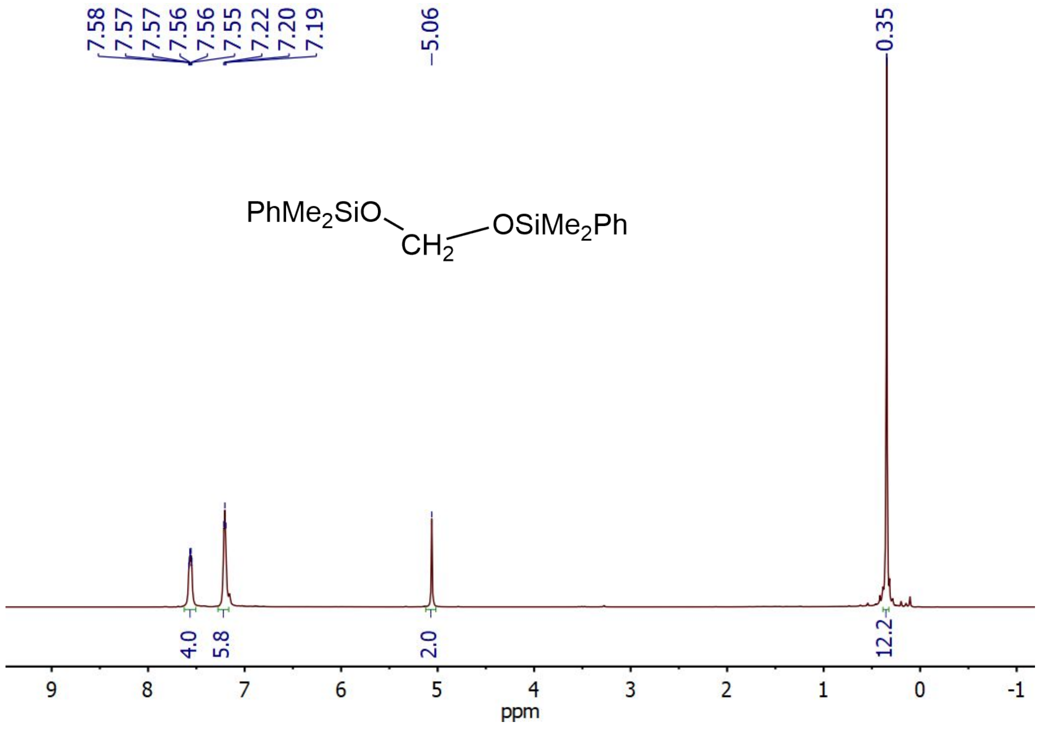

Figure S5. ^{1}H NMR spectrum of **2b** in C_6D_6.

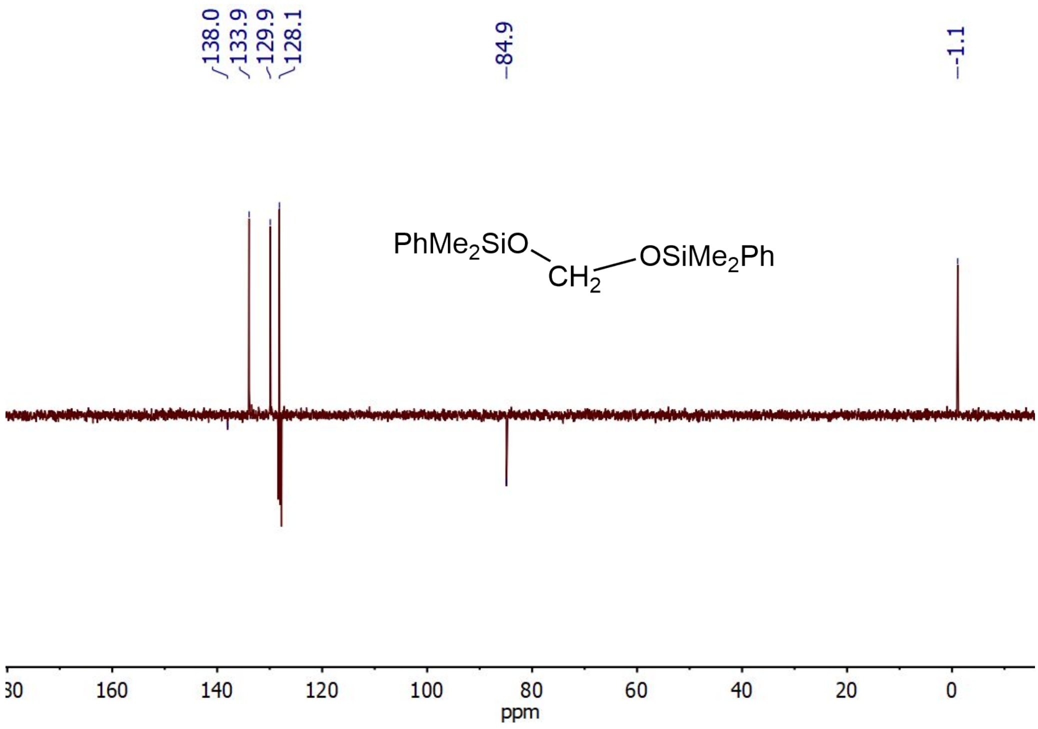

Figure S6. ^{13}C APT NMR spectrum of **2b** in C_6D_6.

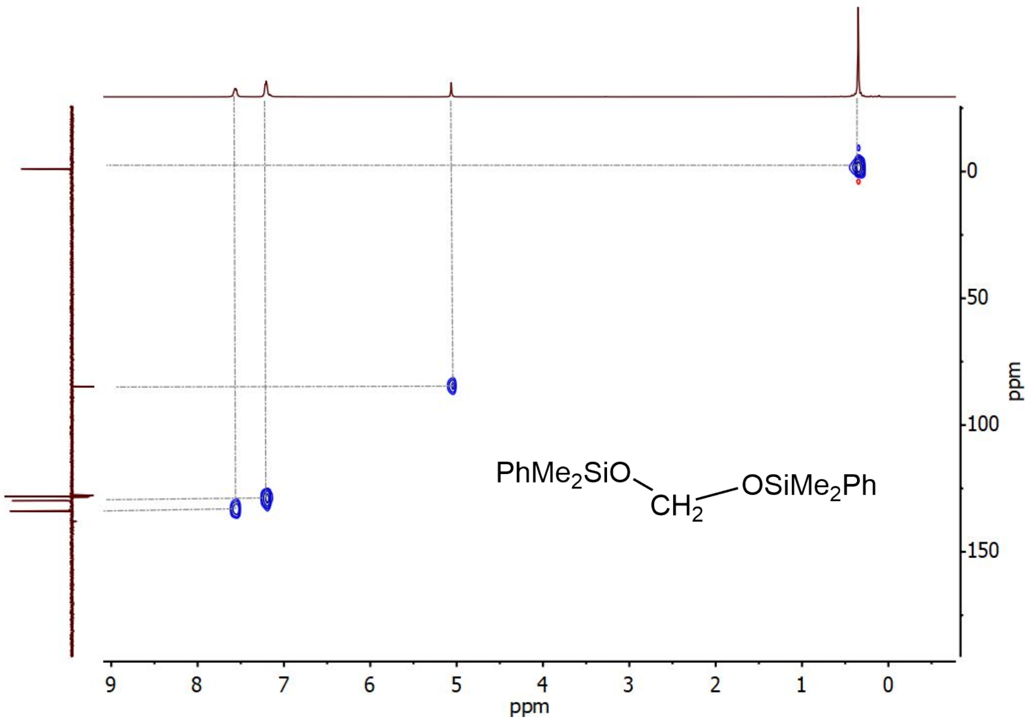

Figure S7. ^1H-^{13}C HSQC NMR spectrum of **2b** in C_6D_6.

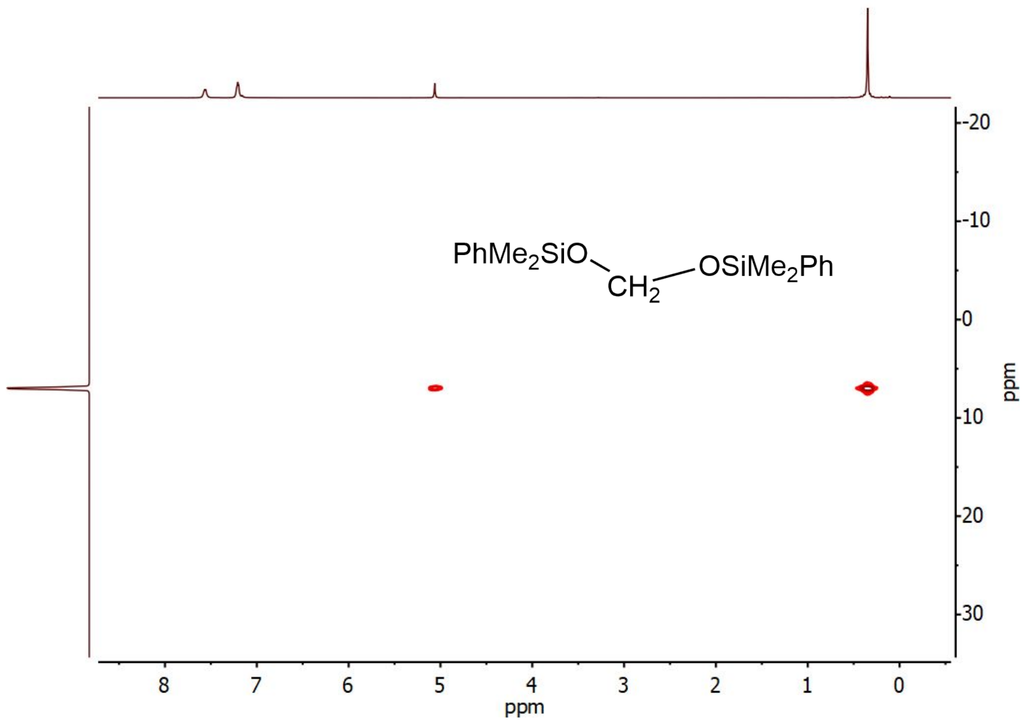

Figure S8. ^1H-^{29}Si HMBC NMR spectrum of **2b** in C_6D_6.

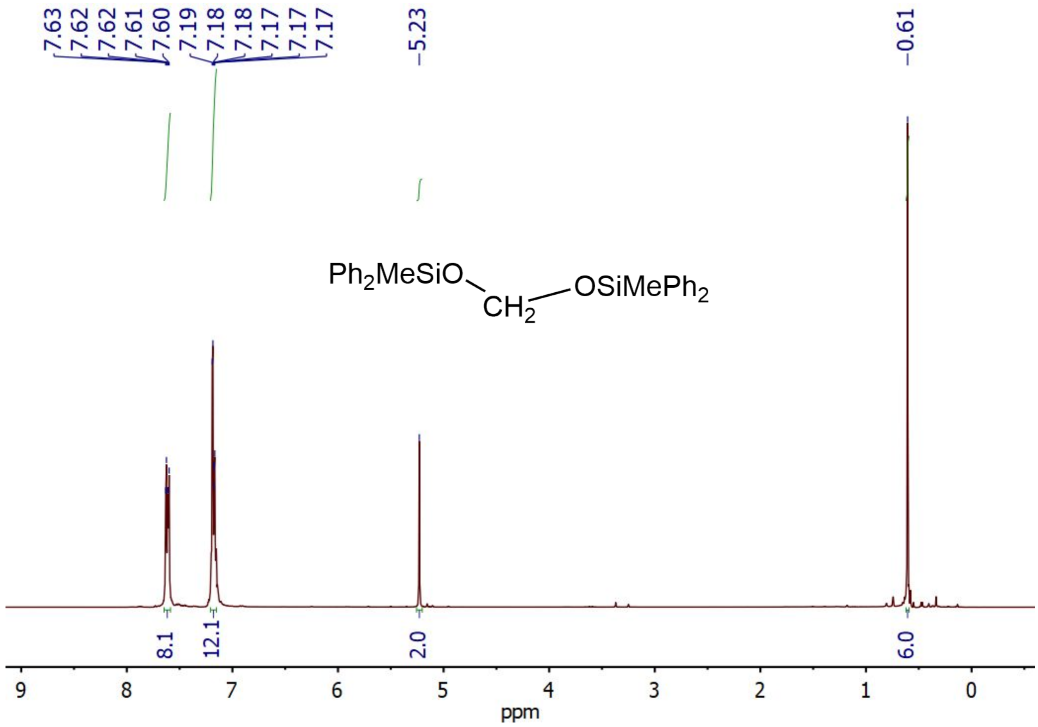

Figure S9. ^{1}H NMR spectrum of **2c** in C_6D_6.

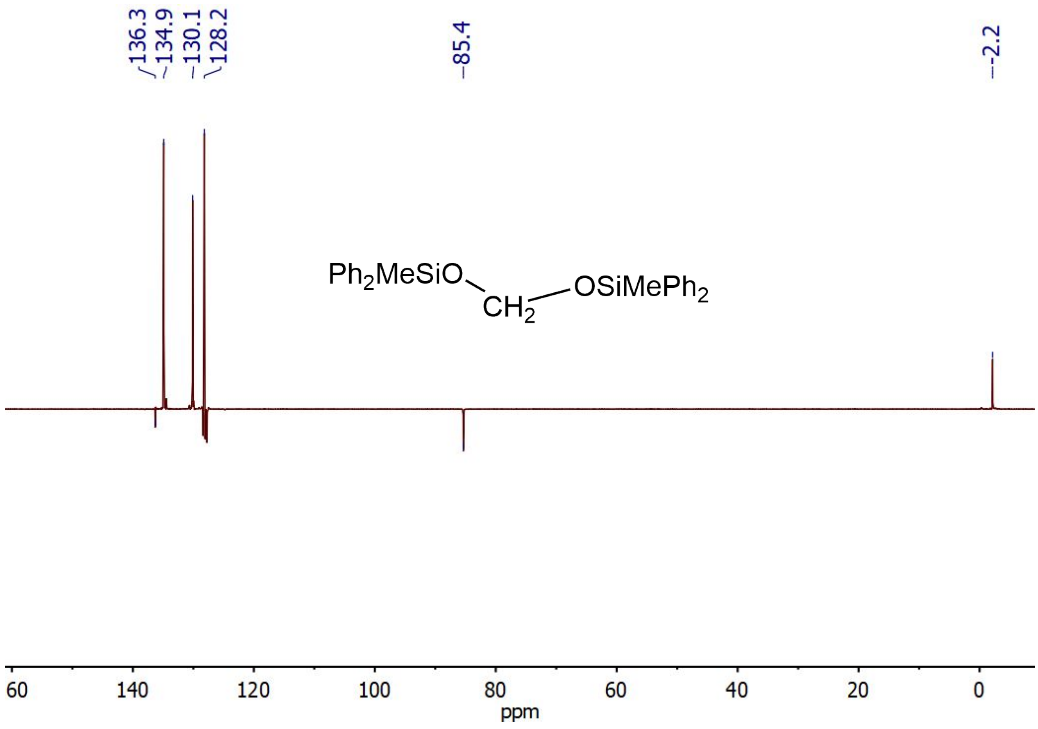

Figure S10. ^{13}C APT NMR spectrum of **2c** in C_6D_6.

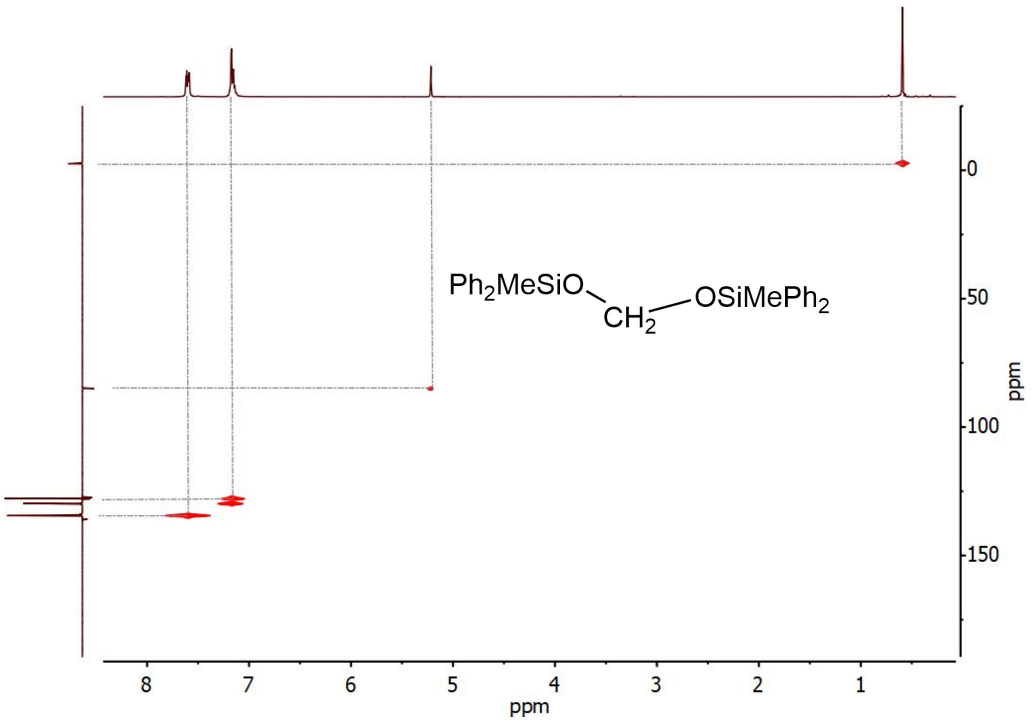

Figure S11. ^{1}H-^{13}C HSQC NMR spectrum of **2c** in C_6D_6.

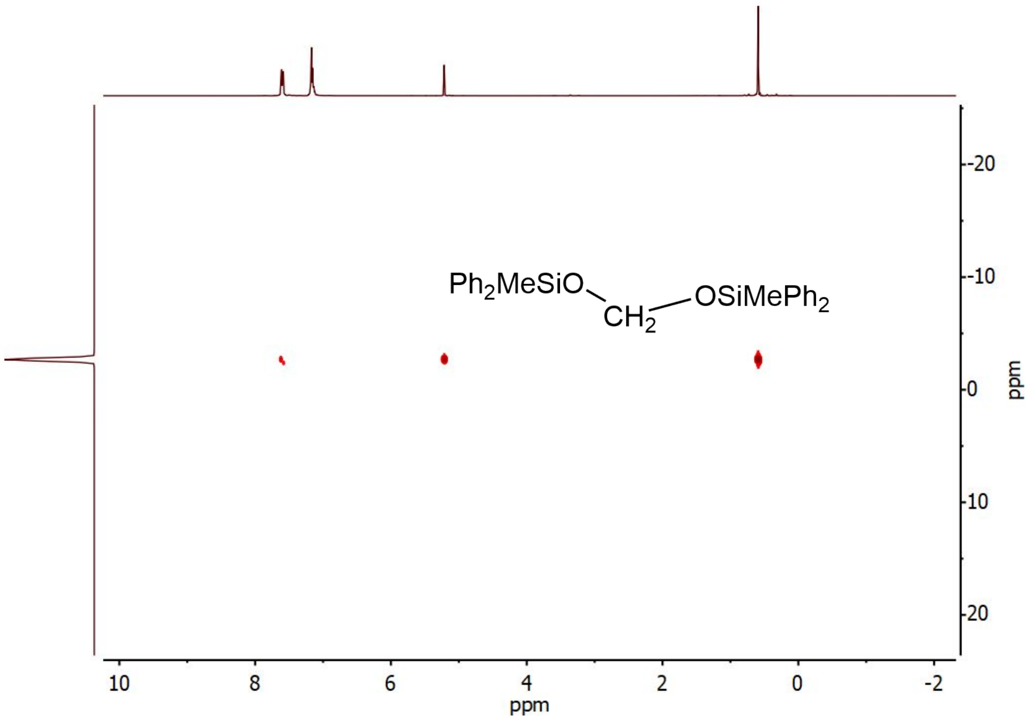

Figure S12. ^{1}H-^{29}Si HMBC NMR spectrum of **2c** in C_6D_6.

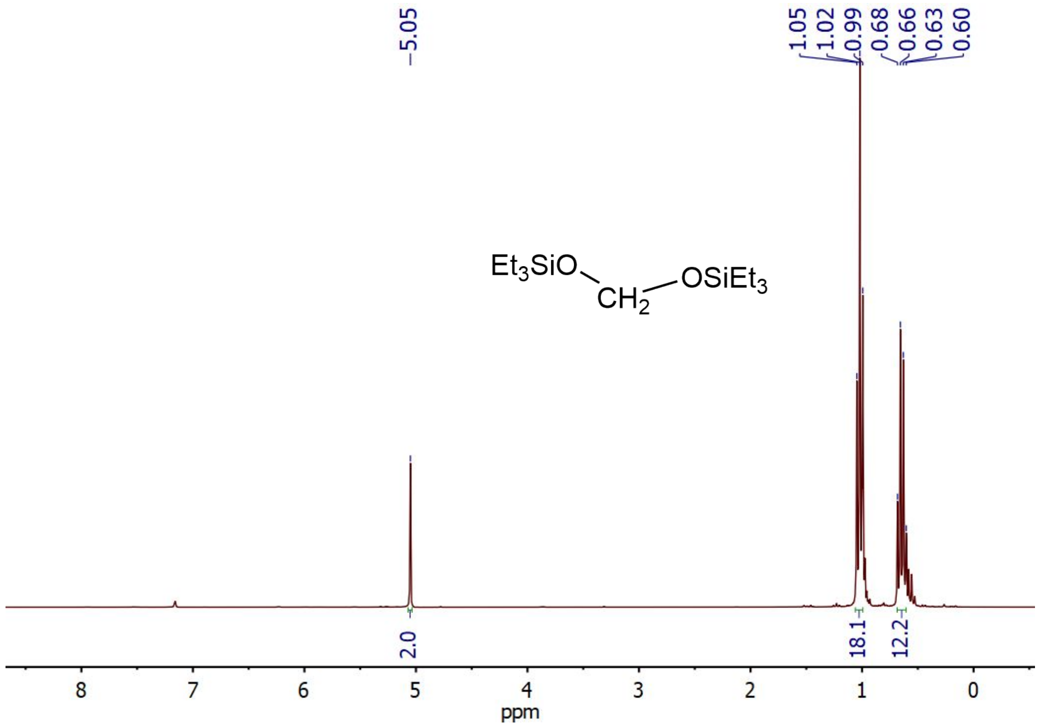

Figure S13. ^{1}H NMR spectrum of **2d** in C_6D_6.

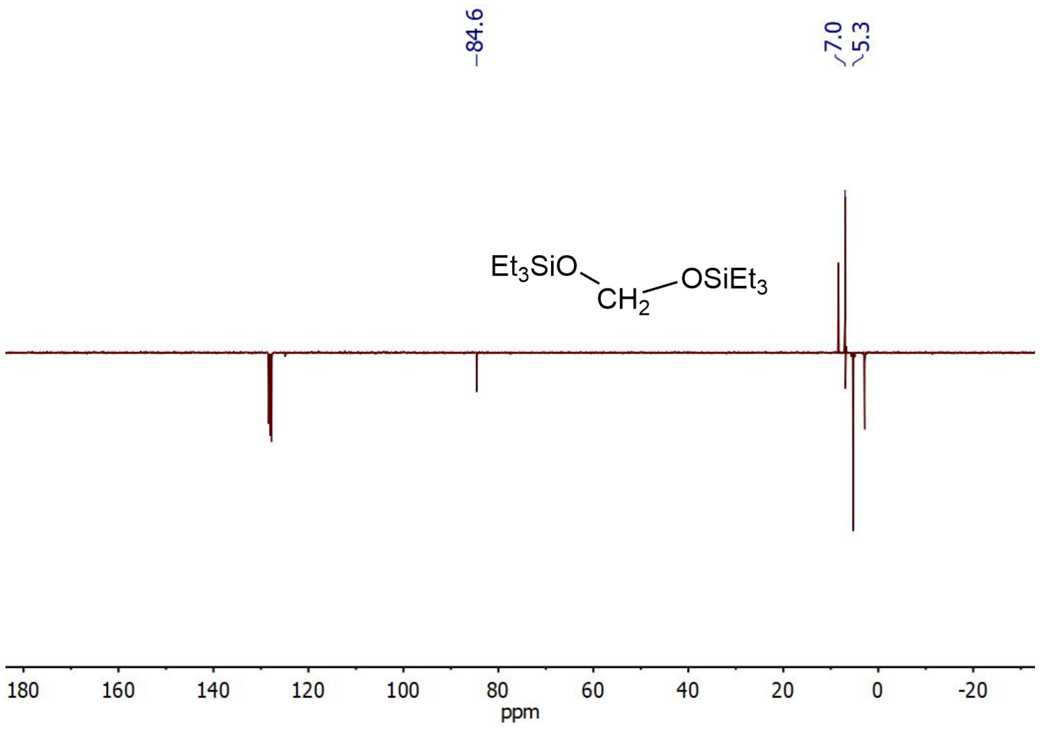

Figure S14. ^{13}C APT NMR spectrum of **2d** in C_6D_6.

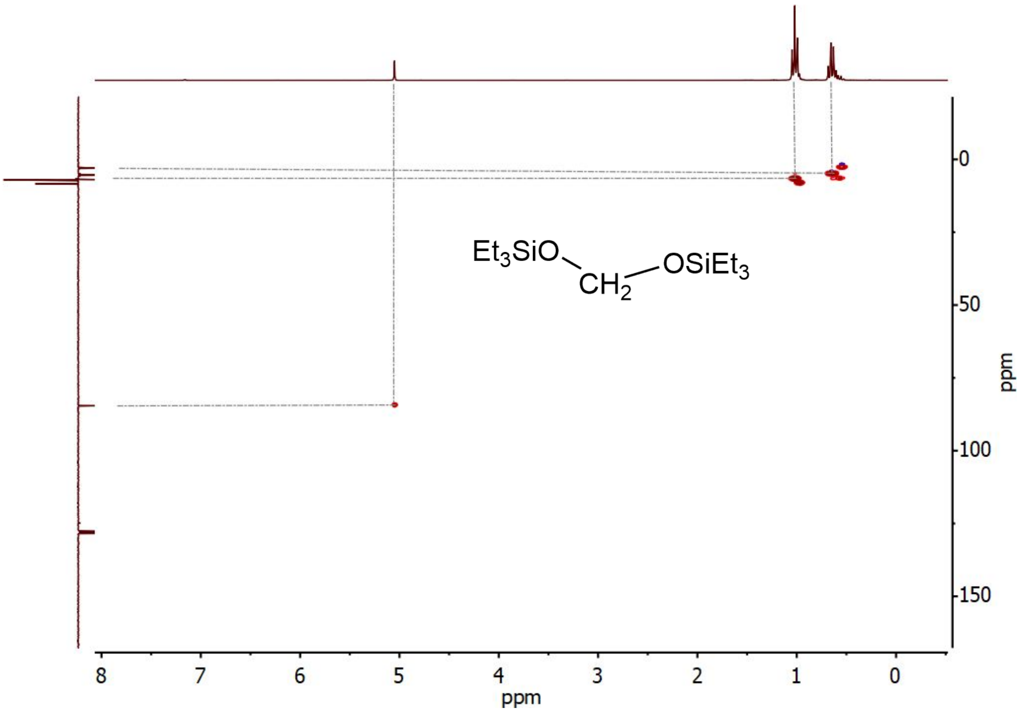

Figure S15. ^{1}H-^{13}C HSQC NMR spectrum of **2d** in C_6D_6.

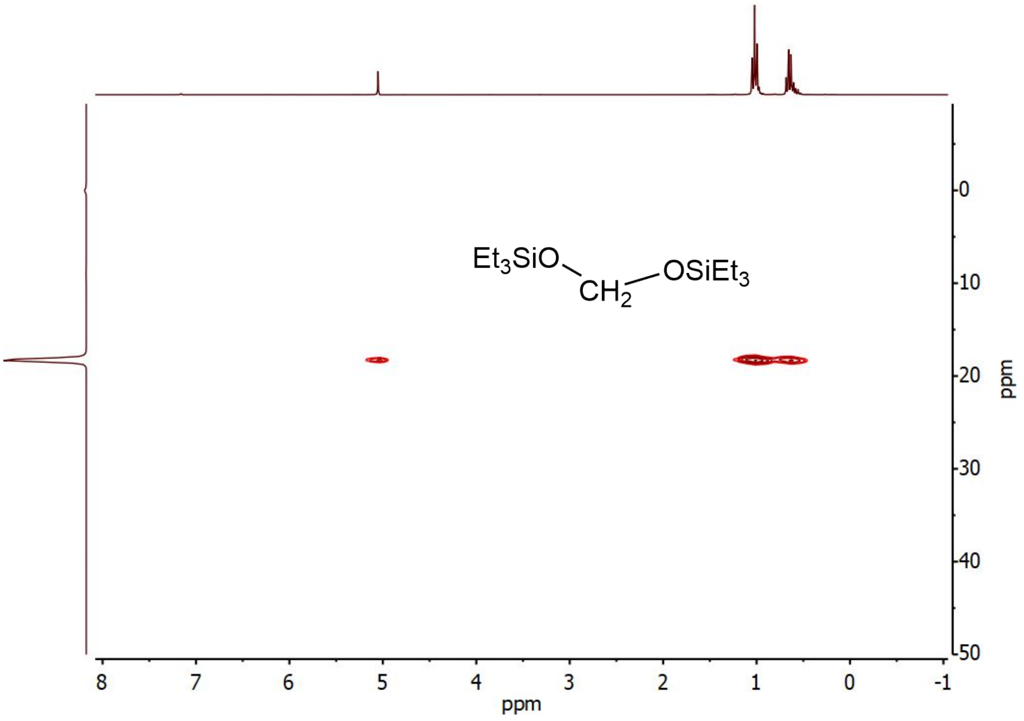

Figure S16. ^{1}H-^{29}Si HMBC NMR spectrum of **2d** in C_6D_6.

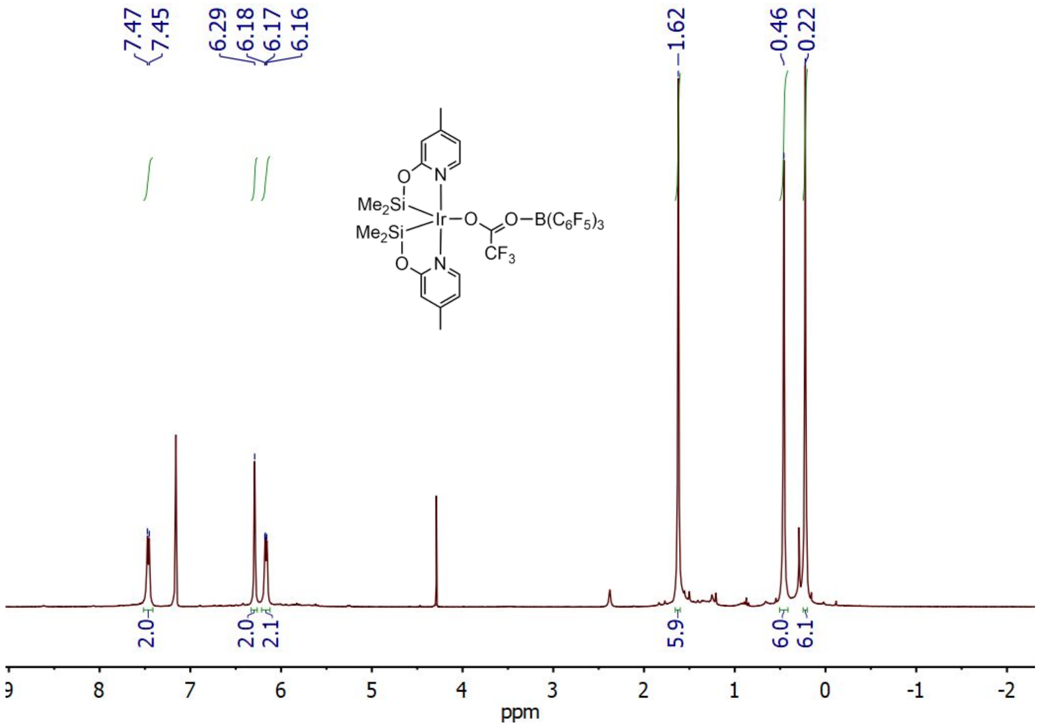

Figure S17. ${}^{1}H$ NMR spectrum of **3** in C_6D_6.

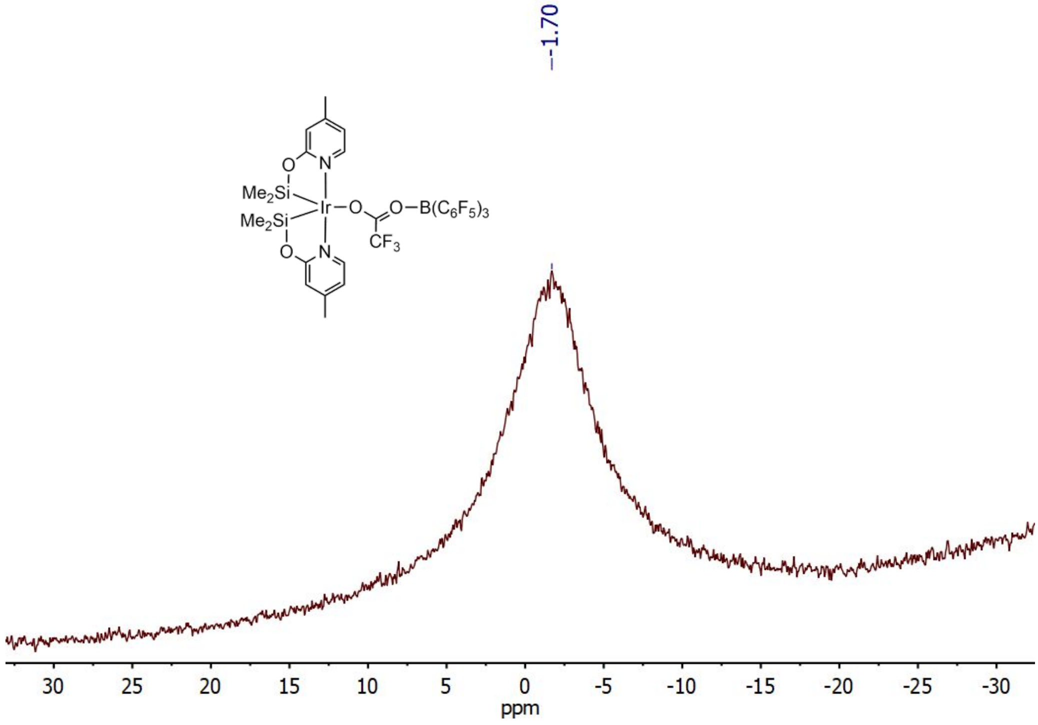

Figure S18. ${}^{11}B$ NMR spectrum of **3** in C_6D_6.

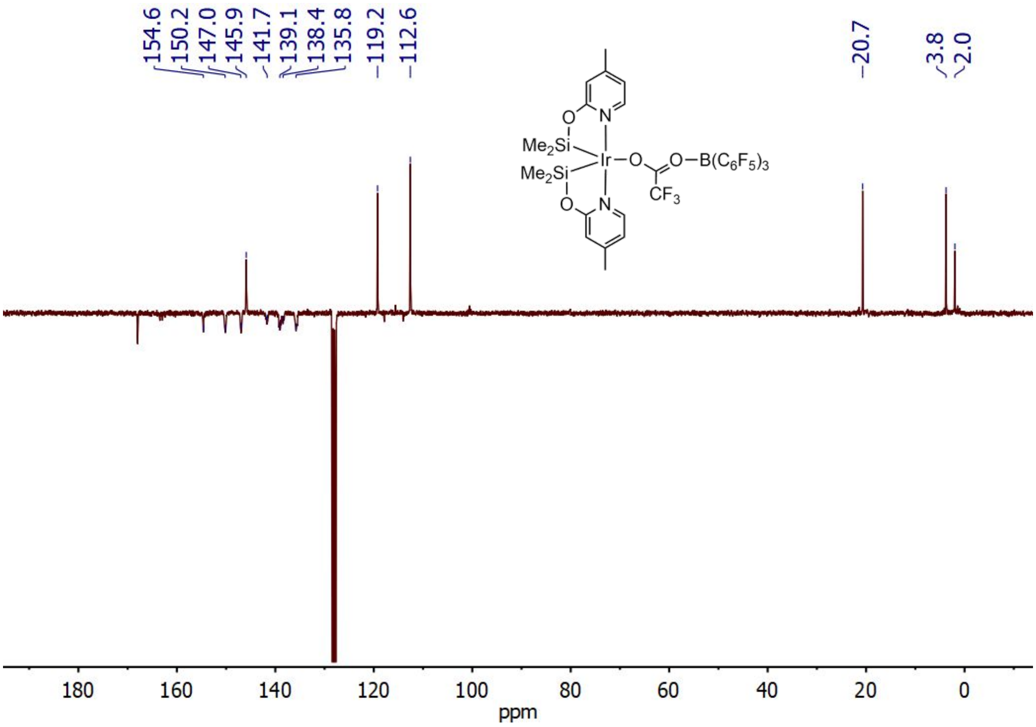

Figure S19. ^{13}C APT NMR spectrum of **3** in C_6D_6.

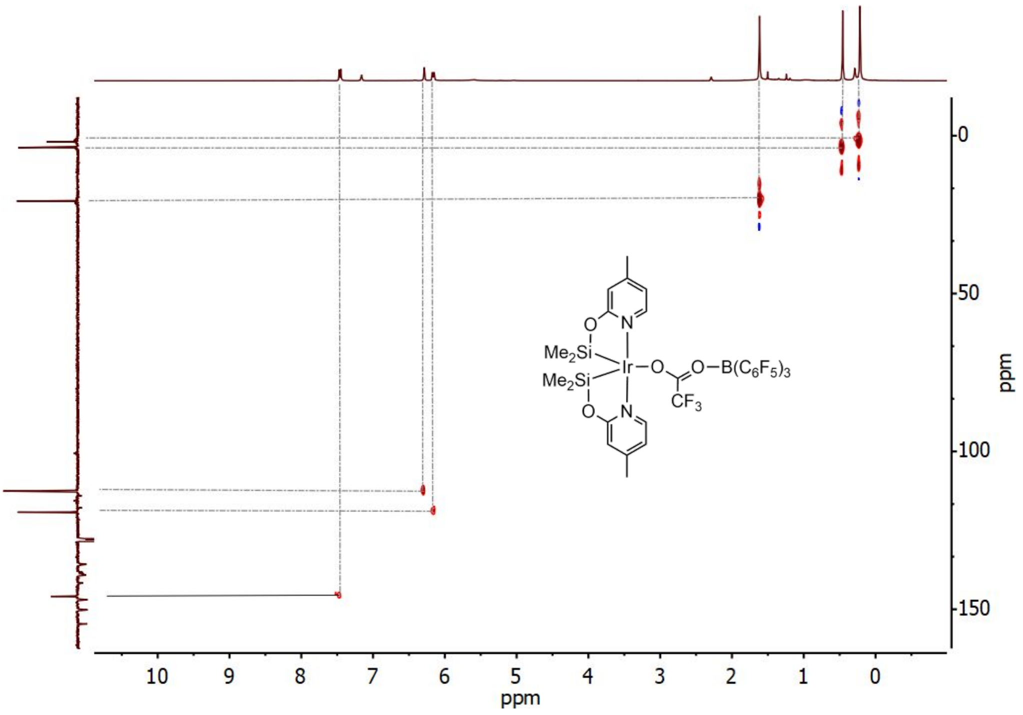

Figure S20. ^{1}H-^{13}C HSQC NMR spectrum of **3** in C_6D_6.

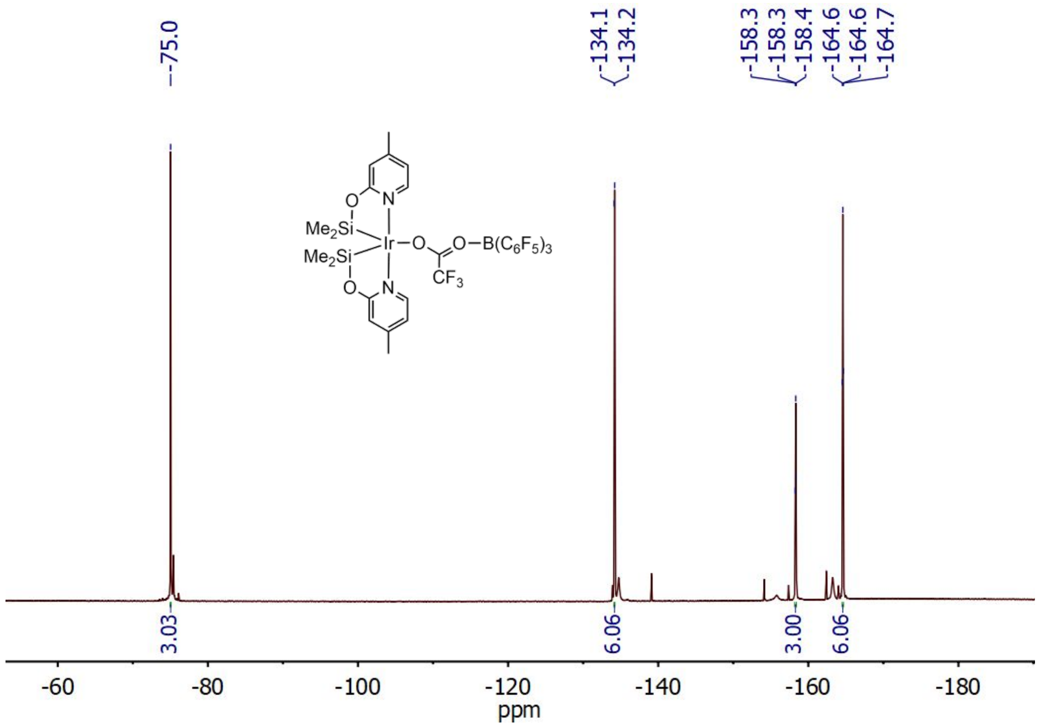

Figure S21. ^{19}F NMR spectrum of **3** in C_6D_6.

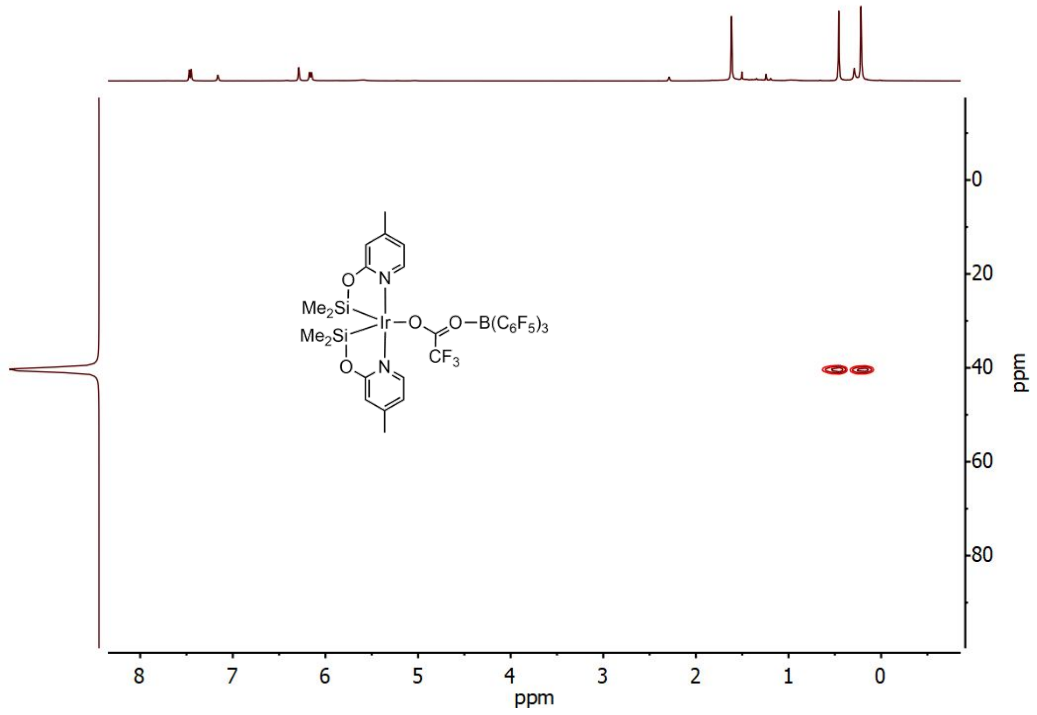

Figure S22. ^{1}H-^{29}Si HMBC NMR spectrum of **3** in C_6D_6.

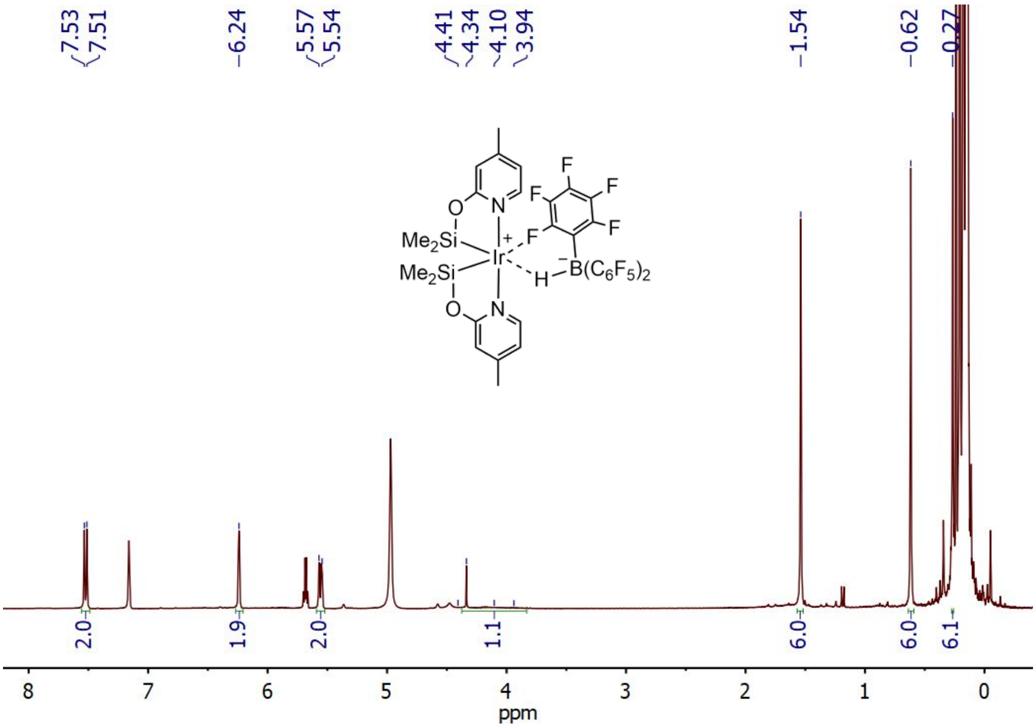

Figure S23. ^{1}H NMR spectrum of **4** in C_6D_6.

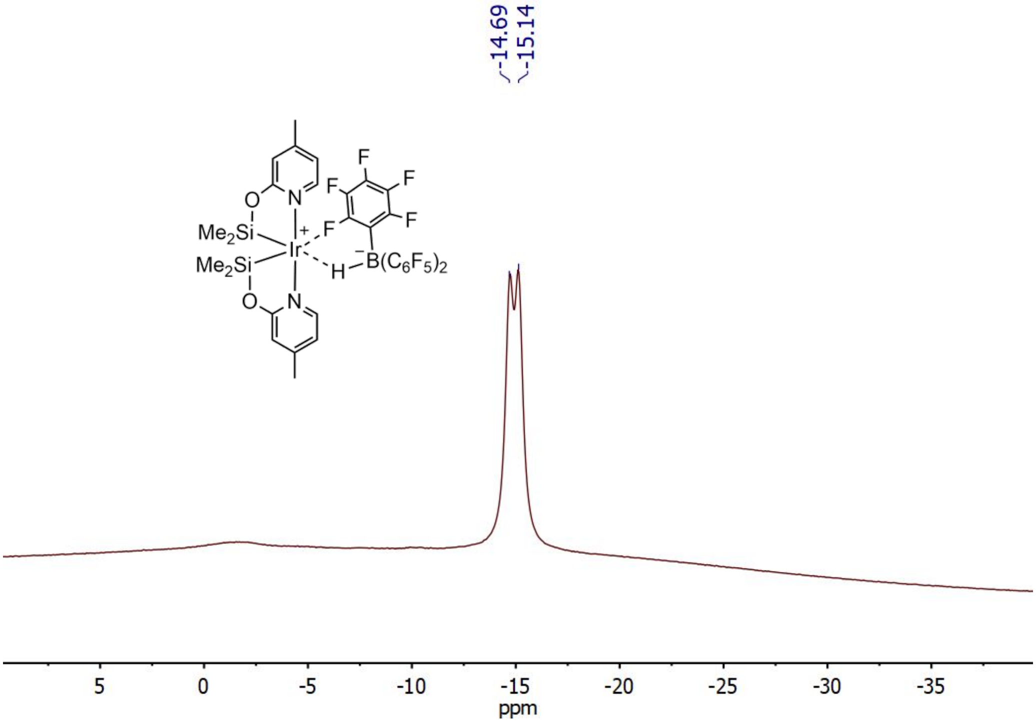

Figure S24. ^{11}B NMR spectrum of **4** in C_6D_6.

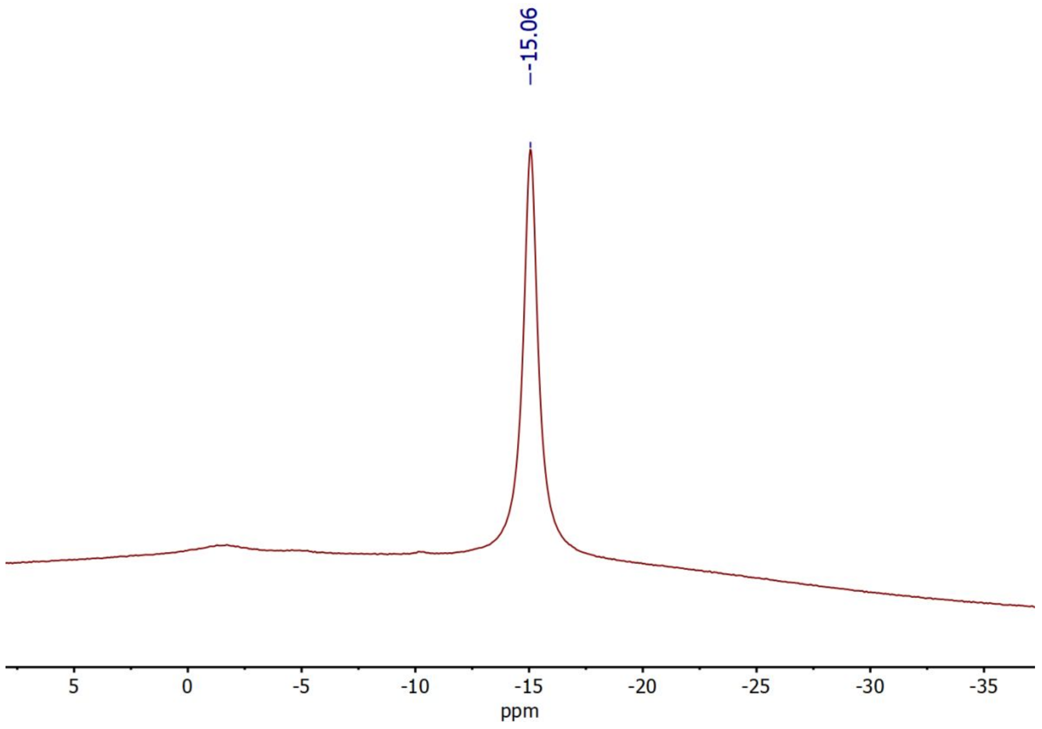

Figure S25. $^{11}B\{^{1}H\}$ NMR spectrum of **4** in C_6D_6.

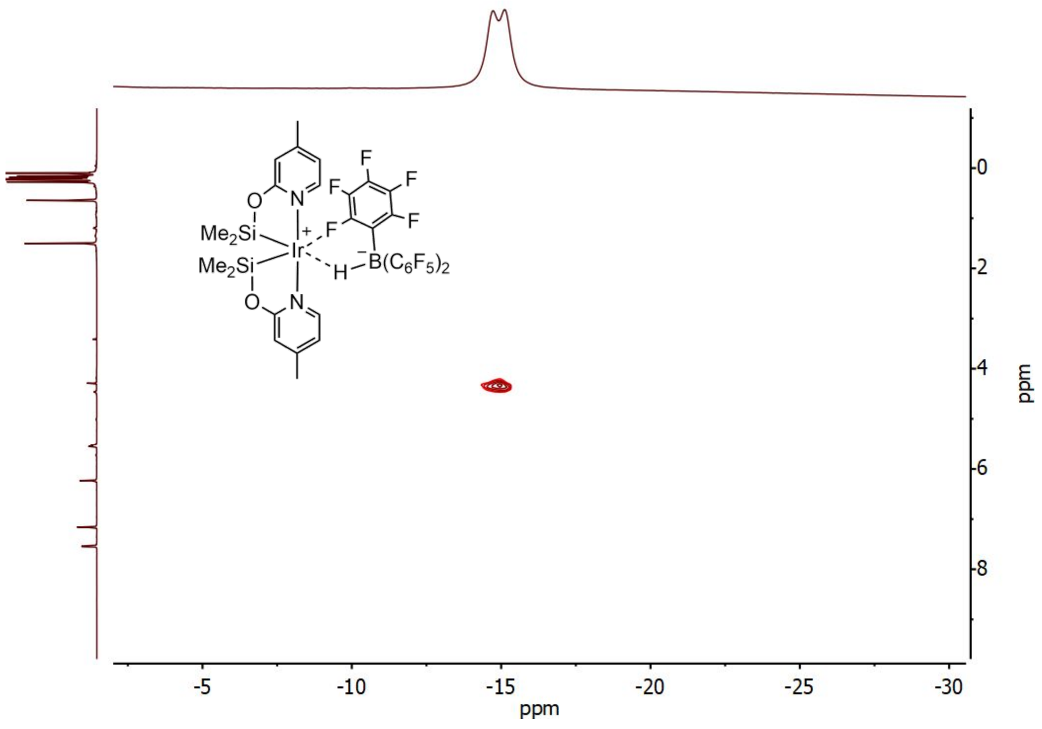

Figure S26. ^{11}B-^{1}H HSQC NMR spectrum of **4** in C_6D_6.

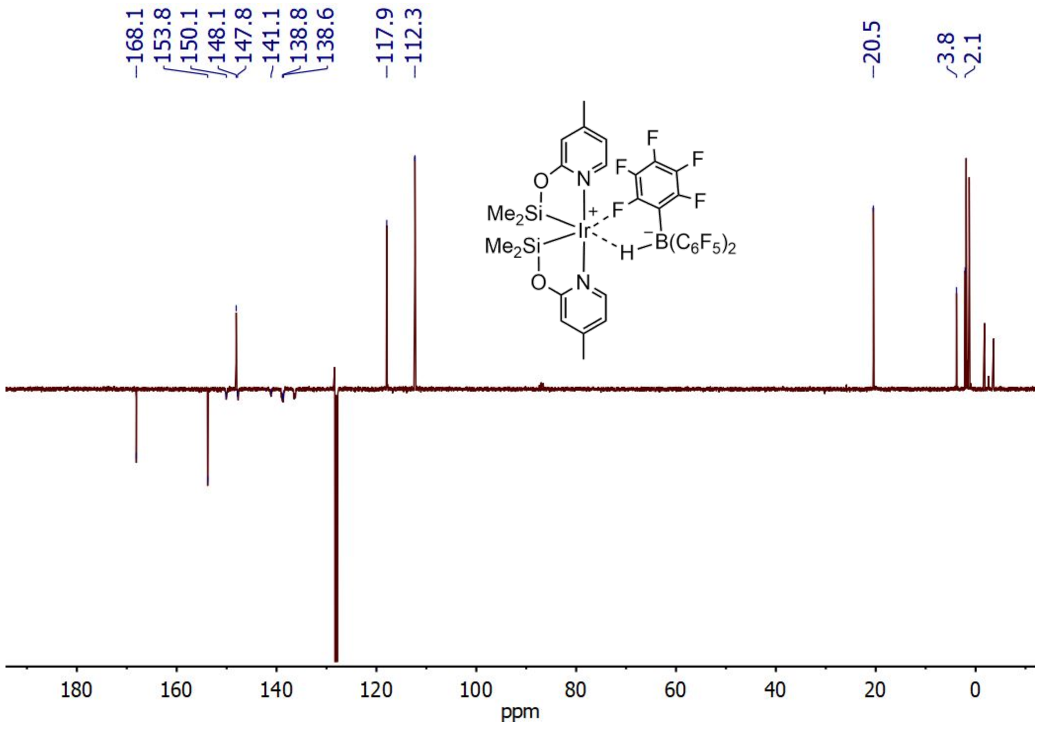

Figure S27. ^{13}C APT NMR spectrum of **4** in C_6D_6.

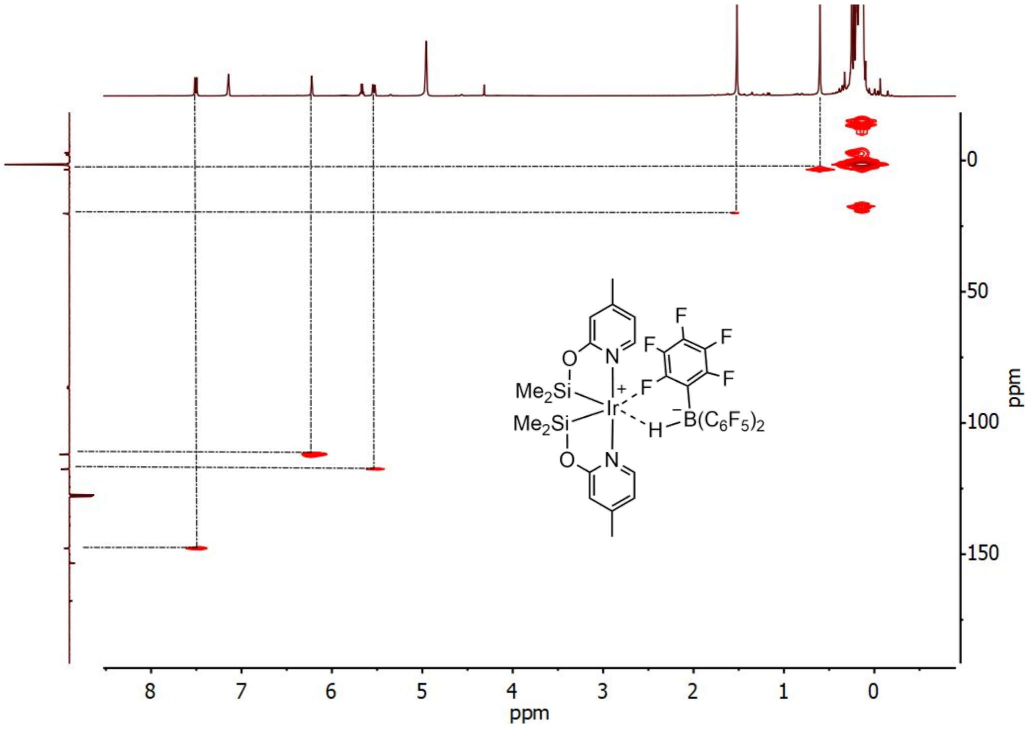

Figure S28. ^{1}H-^{13}C HSQC NMR spectrum of **4** in C_6D_6.

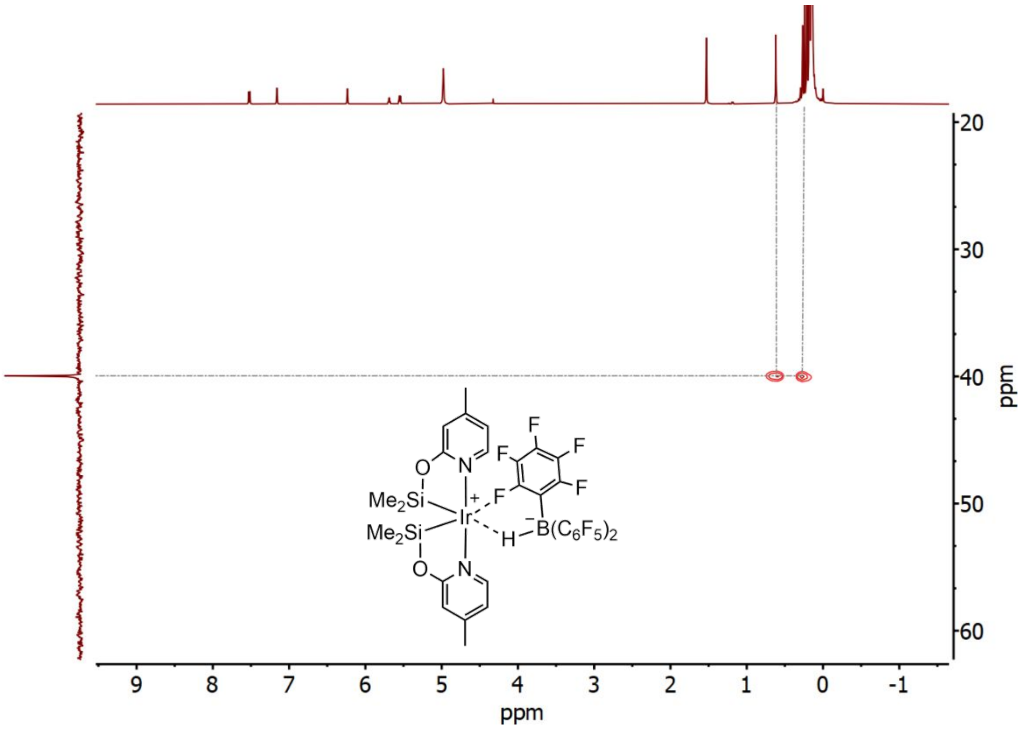

Figure S29. ^{1}H-^{29}Si HMBC NMR spectrum of **4** in C_6D_6.

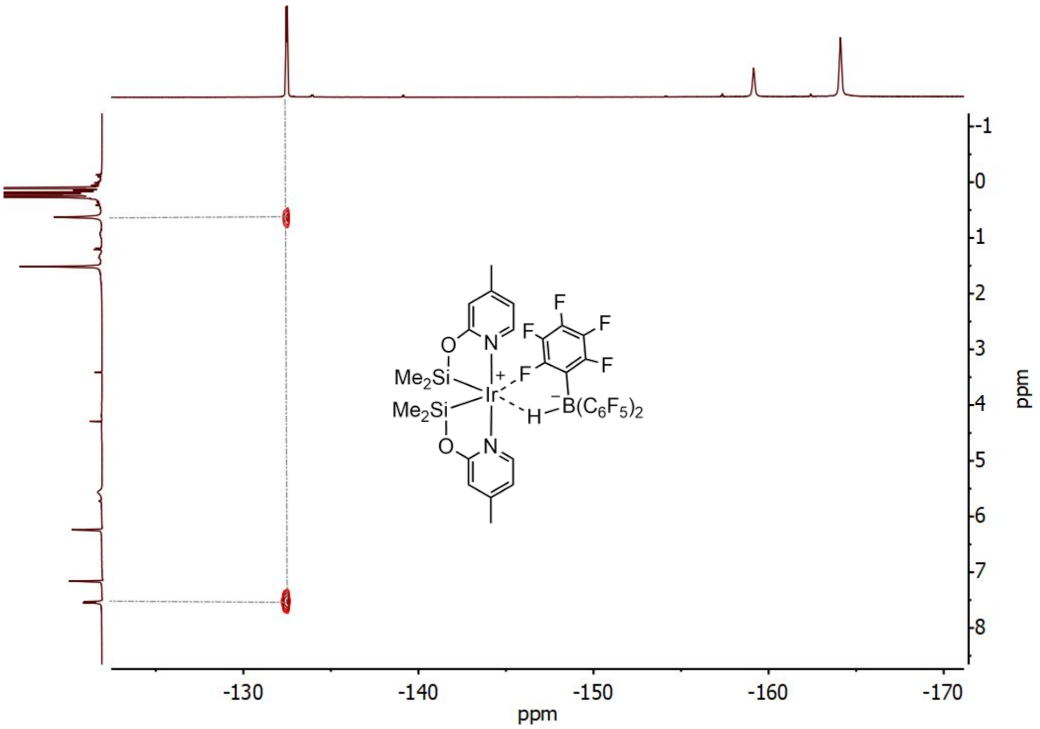

Figure S30. ^{19}F-^{1}H HOESY NMR spectrum of **4** in C_6D_6. This experiment shows a dipolar coupling between the *ortho*-H of the pyridinic ring and the protons of the SiMe group of the ligand with the *ortho*-F of the C_6F_5 groups.

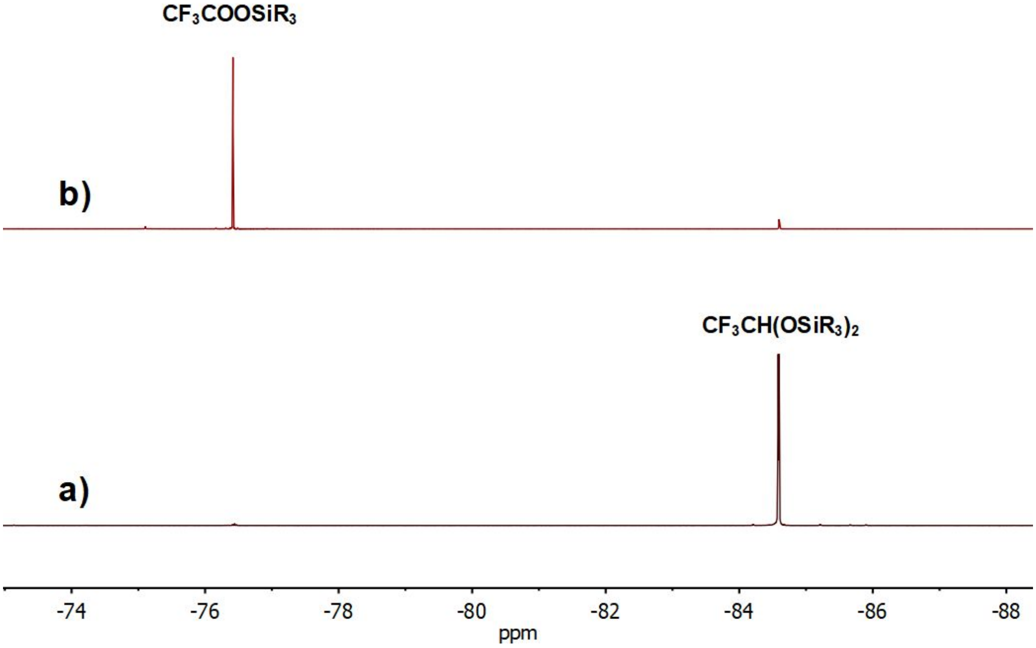

Figure S31. ^{19}F NMR spectra of the reaction of **3** with a) an excess of HMTS, and b) one equivalent of HMTS.

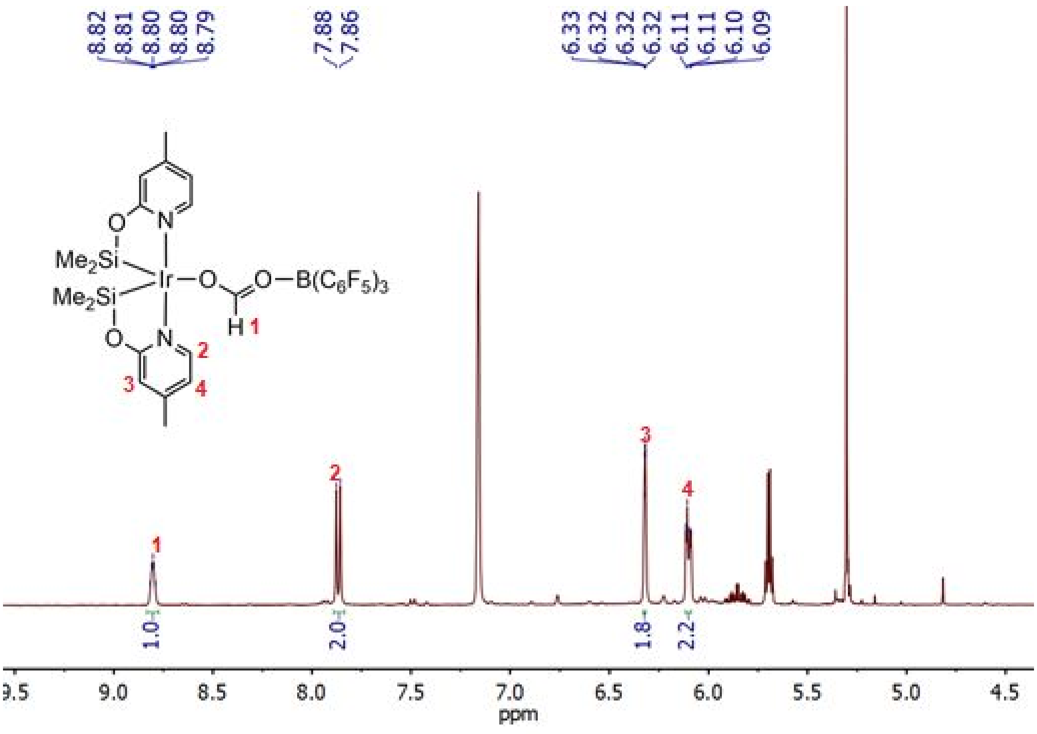

Figure S32. Selected region from the ^{1}H NMR spectrum of **5** in C_6D_6.

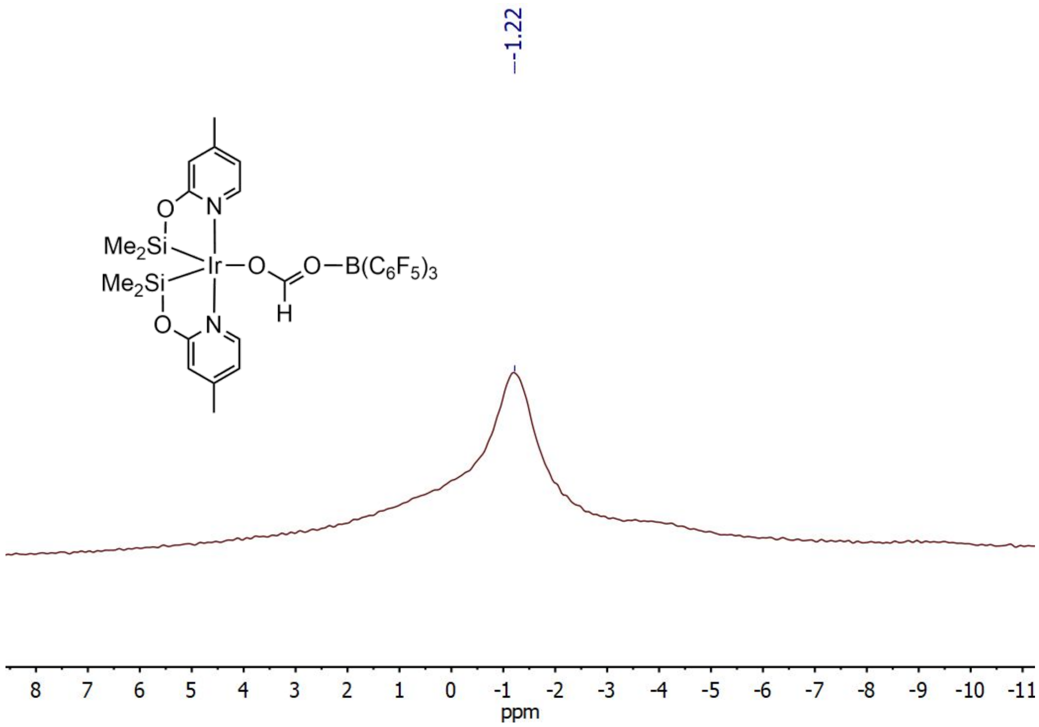

Figure S33. ^{11}B NMR spectrum of **5** in C_6D_6.

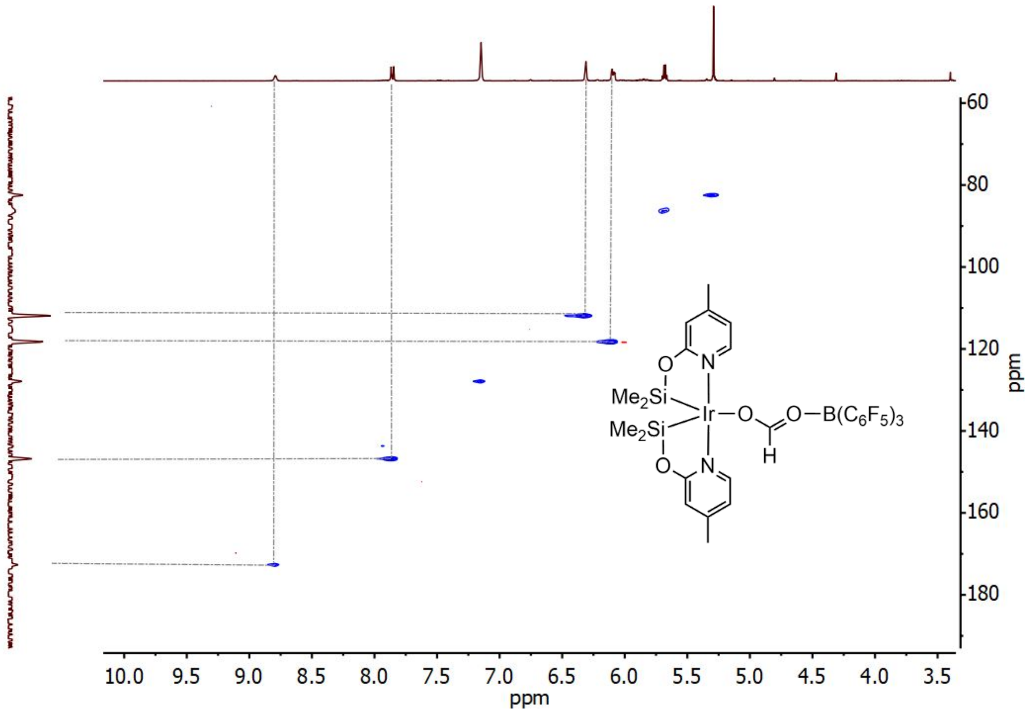

Figure S34. Selected region from the ^{1}H-^{13}C HSQC NMR spectrum of **5** in C_6D_6.

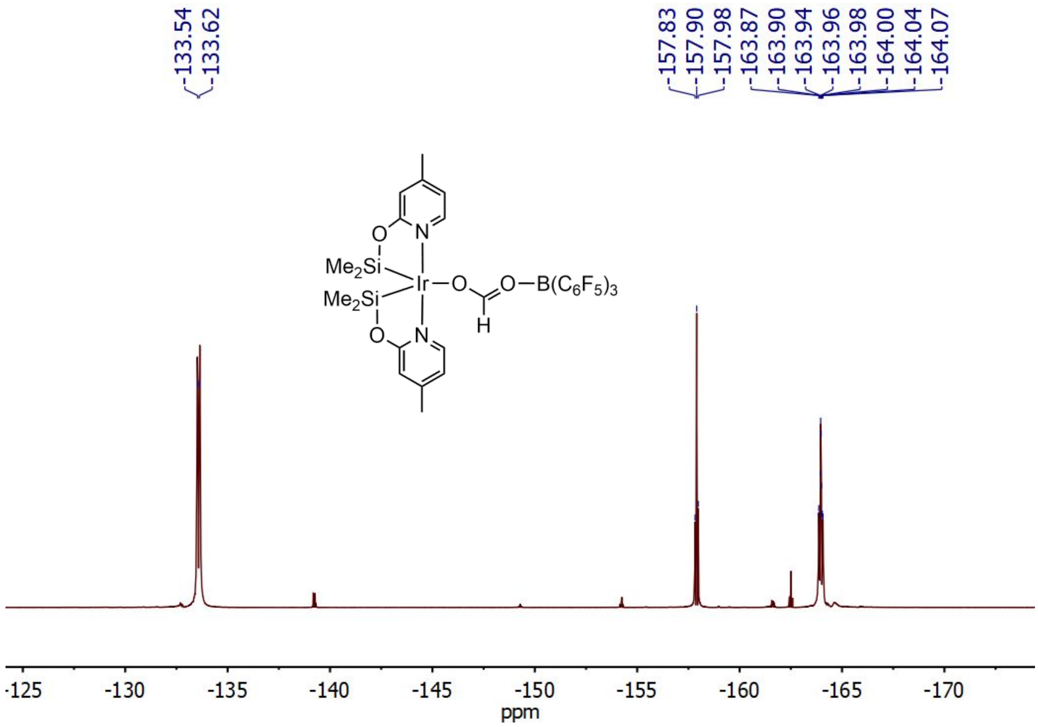

Figure S35. ^{19}F NMR spectrum of **5** in C_6D_6.

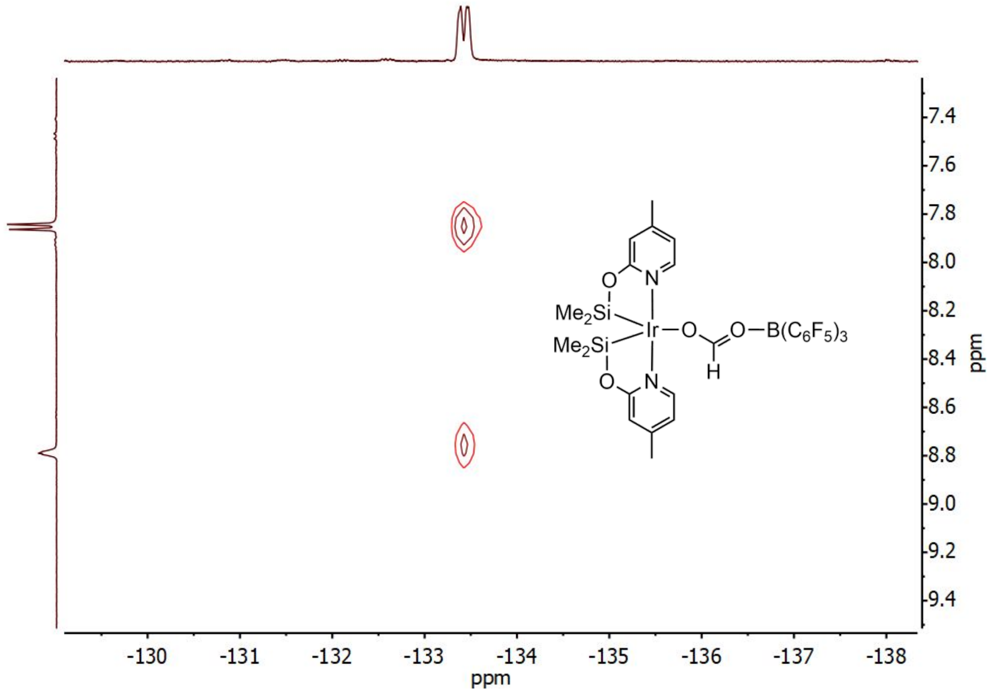

Figure S36. ^{19}F-^{1}H HOESY NMR spectrum of **5** in C_6D_6.

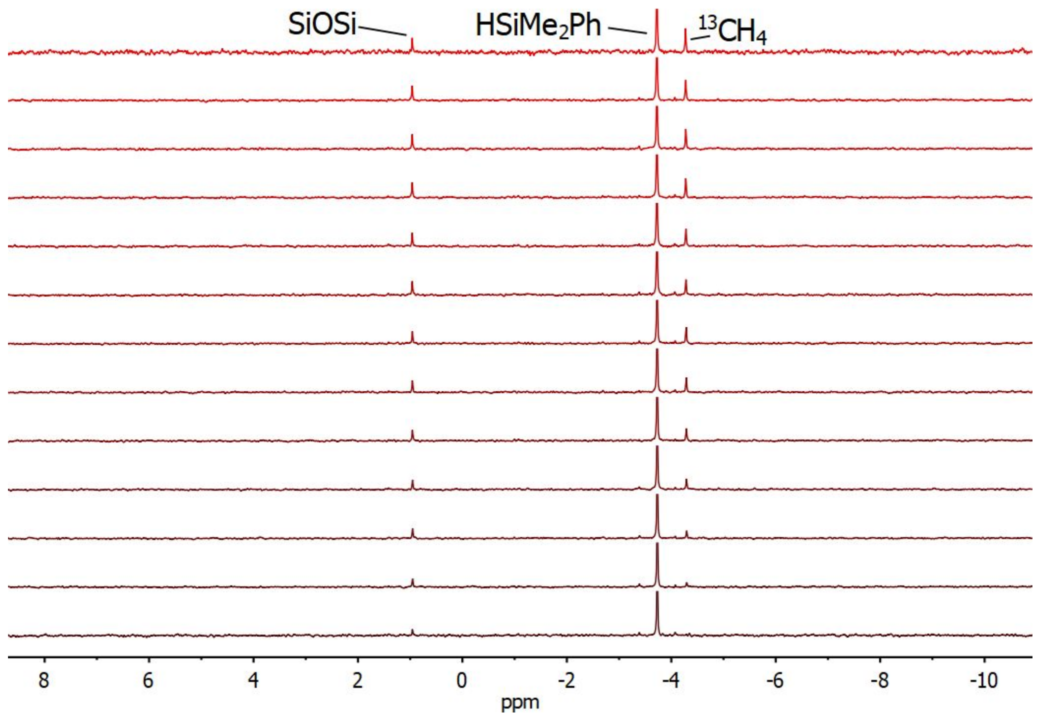

Figure S37. $^{13}C\{^{1}H\}$ NMR (C_6D_6) monitoring of the reaction of ^{13}C isotope-labelled $^{13}CO_2$ (2.7 bar) with $HSiMe_2Ph$ at RT in presence of **1** (1 mol %) and $B(C_6F_5)_3$ (2 mol %). Time between each spectrum: 1 hour.

3. Crystal structure determination of 3

X-ray diffraction data were collected on a Bruker D8-VENTURE diffractometer, using Mo κα radiation (λ = 0.71073 Å). Single crystal was mounted under oil in a MiTeGen mount and cooled to 100(2) K with an open-flow nitrogen gas. Data were collected using φ and ω narrow scans. Diffracted intensities were integrated and corrected for absorption effects using SAINT[S1] and SADABS[S2] programs, included in APEX4 package. The structure was solved by direct methods with SHELX[S3] and refined by full-matrix least squares on F^2 with SHELXL program[S4] with Olex2 program.[S5]

The molecular structure, depicted in Figure S38, evidences the trigonal bipyramidal coordination of the iridium atom, with nitrogen atoms occupying apical positions. The coordination of the borane ligand hardly influences the geometry of the $[Ir(\kappa^2\text{-NSi}^{Me})_2]$ fragment, whose geometrical parameters nicely agree with those of the parent compound **1**, with short Ir-Si bond lengths (2.2526(11) and 2.2599(11) Å).

The Ir-O bond length in **3** (2.285(3) Å) is shorter than those found in compound **1** (2.363(3) and 2.418(3) Å), and related $[Ir(CF_3CO_2)(\kappa^2\text{-NSi}^{iPr2})_2]$ complexes (2.413(3) and 2.421(3) Å).[S6] Geometrical parameters of OCOB fragment are similar to those observed the few reported examples of metal-coordinated tris (pentafluorophenyl)formyloxyborate),[S7-S11] the O(3)-C(17) bond length (1.220(5) Å) is shorter than C(17)-O(4) (1.271(4) Å), and O(4)-B(1) (1.550(5) Å) is not as long as datively bonded carbonyl adducts of $B(C_6F_5)_3$.[S12]

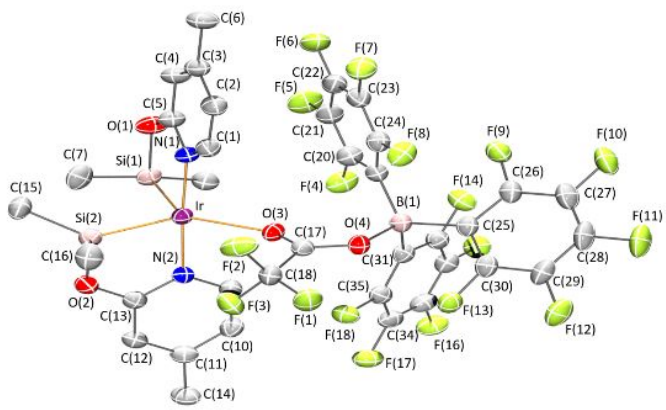

Figure S38. Molecular structure of compound **3**. Hydrogen atoms are omitted for clarity.

Table S1. Selected bonds lengths (Å) and angles (º) of compound **3**, compared to those of compound **1**.[S6]

	**3**	**1**
Ir-Si(1)	2.2599(11)	2.2645(10)
Ir-Si(2)	2.2526(11)	2.2505(11)
Ir-O(3)	2.285(3)	2.363(3)<br>2.418(3)
Ir-N(1)	2.056(3)	2.052(3)
Ir-N(2)	2.031(3)	2.056(3)
Si(1)-Ir-Si(2)	92.16(4)	95.84(4)
Si(1)-Ir-O(3)	135.79(8)	
Si(1)-Ir-N(1)	82.09(9)	82.22(8)
Si(1)-Ir-N(2)	98.22(9)	99.02(9)
Si(2)-Ir-O(3)	132.04(7)	
Si(2)-Ir-N(1)	101.28(10)	97.71(9)
Si(2)-Ir-N(2)	82.25(9)	82.24(9)
O(3)-Ir-N(1)	88.71(11)	
O(3)-Ir-N(2)	88.62(11)	
N(1)-Ir-N(2)	176.45(12)	178.76(12)

Structural data for **3**: $C_{36}H_{24}BF_{18}IrN_2O_4Si_2$; Mr = 1149.76; yellow block, 0.035 × 0.074 × 0.095 mm^3; triclinic $P\bar{1}$; a = 11.5292(14) Å, b = 11.7069(14) Å, c = 16.632(2) Å, α=99.410(5) ^{0}, β=93.813(5) ^{0}, γ=113.109(4)^0; V = 2015.7(4) Å^3, Z = 2, D_c = 1.894 g/cm^3; μ = 3.498 cm^{-1}; min. and max. absorption correction factors: 0.0111 and 0.0298, 2θmax = 56.662°; 99857 reflections measured, 10033 unique; R_{int} = 0.0416; number of data/restraint/parameters 10033/0/583; R_1 = 0.0339 [9339 ref. with I > 2σ(I)], wR(F^2) = 0.0910 (all data); largest difference peak 2.605 e·Å^{-3}.

4. Computational Details.

All DFT theoretical calculations were carried out using the Gaussian09 D.01 program package.[S13] The B3LYP-D3(BJ) exchange correlation functional[S14] has been employed for the geometry optimization and frequency calculations in combination to the def2-SVP basis set[S15] which considers effective core potentials for Ir. Single point calculations at the M06L/def-TZVP level of theory,[S16] including also the SMD approach[S17] for benzene to simulate solvation effects were performed to refine the energetic values. All calculations were done using the "ultrafine" grid. Relative energies are Gibbs free energies are referred to a 1 M standard state using the approximation of Goddard et al.[S18] at 25 °C. Analytical frequency analyses were employed to confirm the nature of the stationary points. Grimme's corrections to vibrational entropies based using RRHO and Free-rotor vibrational entropies[S19]] implemented in Goodvibes software[S20] have been applied. Gibbs free energies have been corrected by supressing the translational entropy term following the approach of Morokuma and co-workers.[S21] An intrinsic reaction path or coordinate scan calculations connecting both minima were performed for flat or unclear transition states. Graphical representations were made using the CYLVIEW software.[S22]

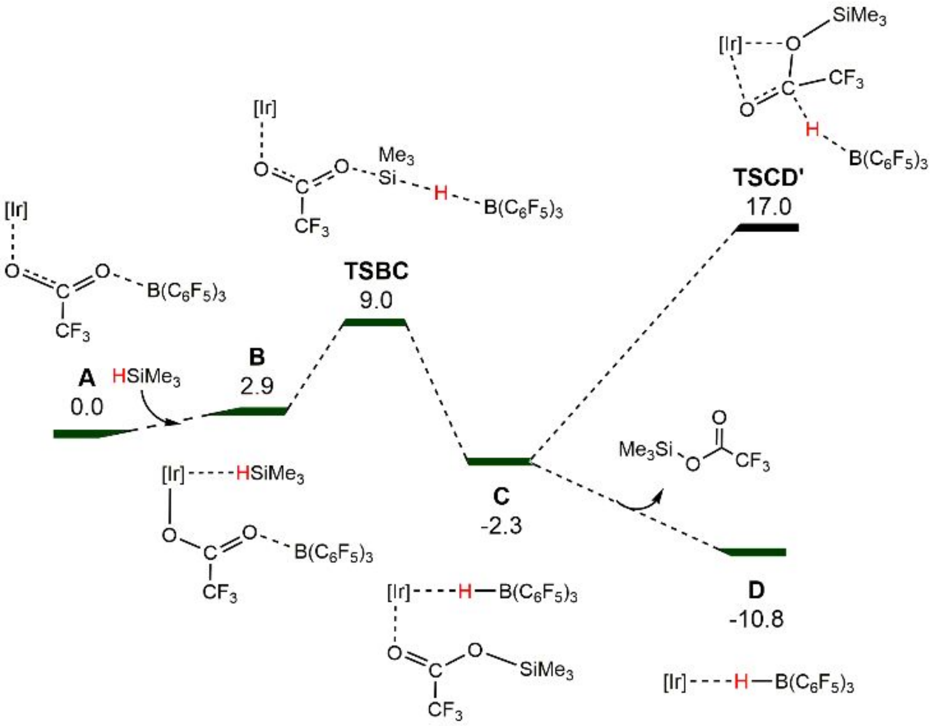

Figure S39. DFT calculated Gibbs free energy profile for the catalyst activation step (in kcal·mol^{-1}) relative to **A**.

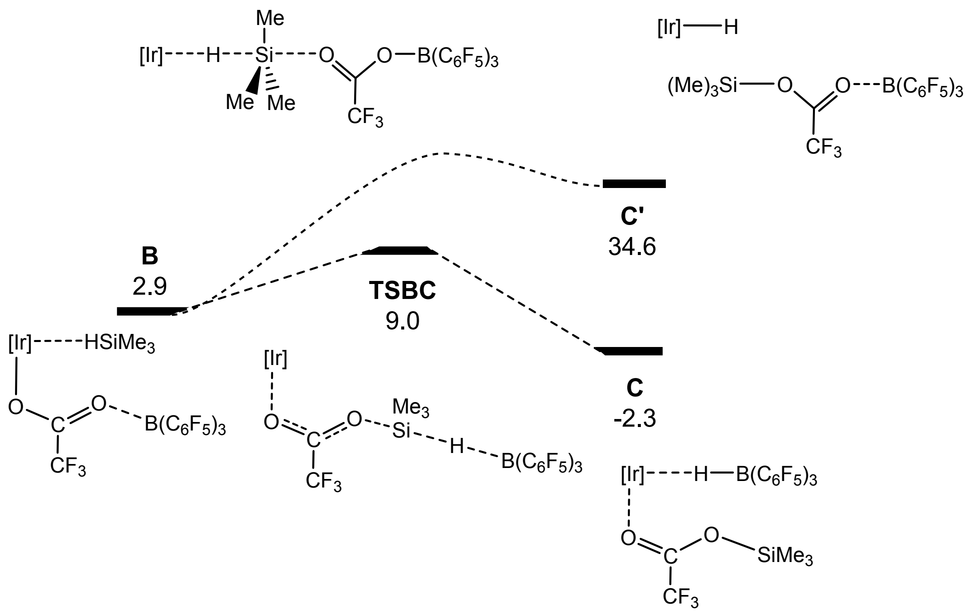

Figure S40. DFT calculated Gibbs free energy profile (in kcal·mol^{-1} relative to **A**) for a compassion between boron promoted Si–H cleavage (leading to **C**) or Ir promoted Si–H cleavage (leading to **C'**).

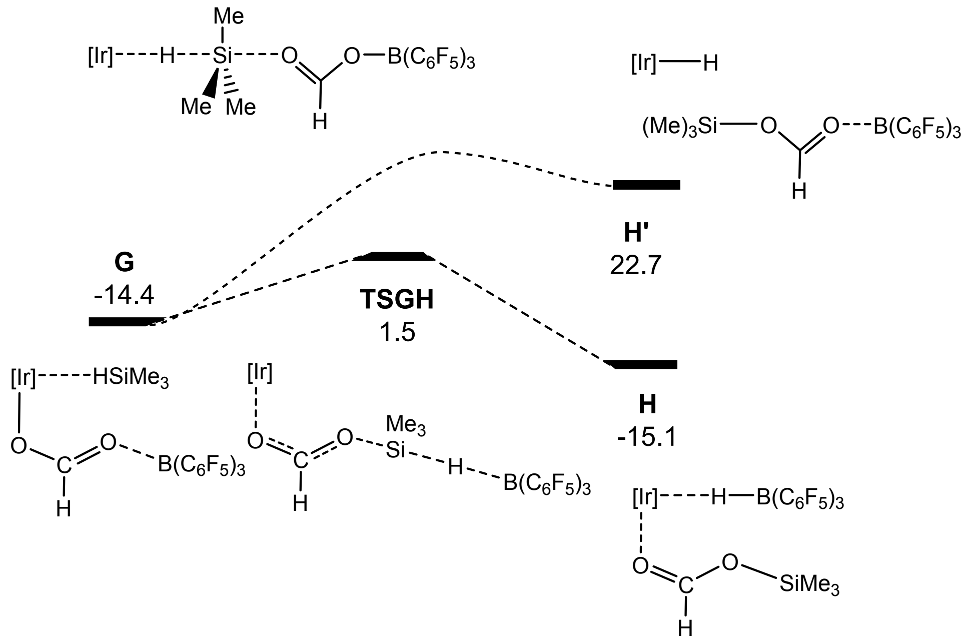

Figure S41. DFT calculated Gibbs free energy profile (in kcal·mol^{-1} relative to **A**) for a compassion between boron promoted Si–H cleavage (leading to **H**) or Ir promoted Si–H cleavage (leading to **H'**).

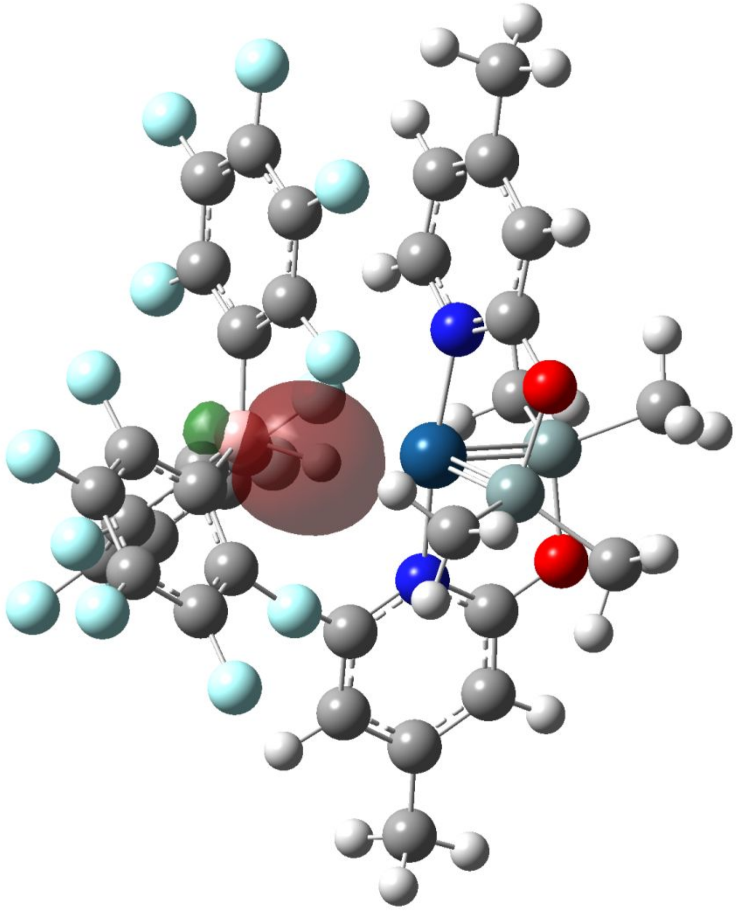

Figure S42. NBO orbital corresponding to the σ B-H bond in intermediate **D**.

Table S2. Energetic values for all DFT calculated structures, absolute energies in a.u. are the relative Gibbs free energies, in kcalmol^{-1}. Geometrical optimizations and vibrational frequencies were calculated using the B3LYPD3/def2-SVP method, E(B3LYPD3/DZ). Energies were further refined by M06L(SMD)/def2-TZVP single point calculations (solvent=benzene), E(M06L(SMD)/TZ). Thermochemical correction calculated at 323.15 K and 1M standard state, including quasi-harmonic corrections by Grimme and removing the translational entropy, Gcorr. Gibbs free energies were calculated using E(M06L(SMD)/TZ) values and Gcorr (G(M06L(SMD)/TZ,323,1M,qh,St)) and relative Gibbs free energies ΔG (to **A** and isolated molecules.)

	E(B3LYPD3/DZ)	E(M06L(SMD)/TZ)	Gcorr	G(M06L(SMD)/TZ,323,1M,qh,S_t)	ΔG
**A**	-4299.69371	-4303.36641	0.48185	-4302.88456	0
**B**	-4709.44188	-4713.26925	0.59317	-4712.67607	2.9
**TSBC**	-4709.41715	-4713.25847	0.59204	-4712.66643	9
**C**	-4709.45686	-4713.28145	0.59710	-4712.68436	-2.3
**D**	-3774.44227	-3777.56995	0.46796	-3777.10198	-10.8
**TSCD'**	-4709.42044	-4713.24762	0.59390	-4712.65372	17
**E**	-3962.90426	-3966.21906	0.47494	-3965.74413	-6.8
**TSEF**	-3962.86763	-3966.19361	0.47840	-3965.71521	11.4
**F**	-3962.91819	-3966.23573	0.48160	-3965.75413	-13.1
**G**	-4372.66767	-4376.14552	0.59305	-4375.55247	-14.4
**TSGH**	-4372.62308	-4376.11632	0.58911	-4375.52721	1.5
**H**	-4372.67183	-4376.14929	0.59576	-4375.55353	-15.1
**TSHI**	-4372.63671	-4376.11957	0.59246	-4375.52712	1.5
**I**	-4372.68174	-4376.15617	0.60082	-4375.55535	-16.2
**TSIJ**	-4782.36968	-4786.02445	0.70983	-4785.31463	7
**D**+bis(silyl)aceta[illegible]	-1007.96316	-1008.50094	0.22794	-1008.27300	-30.9
**C'**	-4709.39260	-4713.21891	0.59333	-4712.62558	34.6
**H'**	-4372.61048	-4376.10027	0.58967	-4375.51061	22.7
$HSiMe_3$	-409.72219	-409.89779	0.10159	-409.79620	
$B(C_6F_5)_3$	-2206.69278	-2208.90669	0.11191	-2208.79478	
trifluorosilylacet[illegible]	-934.98803	-935.70982	0.11385	-935.59597	
Silylformate	-598.20113	-598.57073	0.11374	-598.45699	
CO2	-188.44468	-188.65370	0.00515	-188.64855	

Figure S43. Geometrical representation of all DFT calculated species.

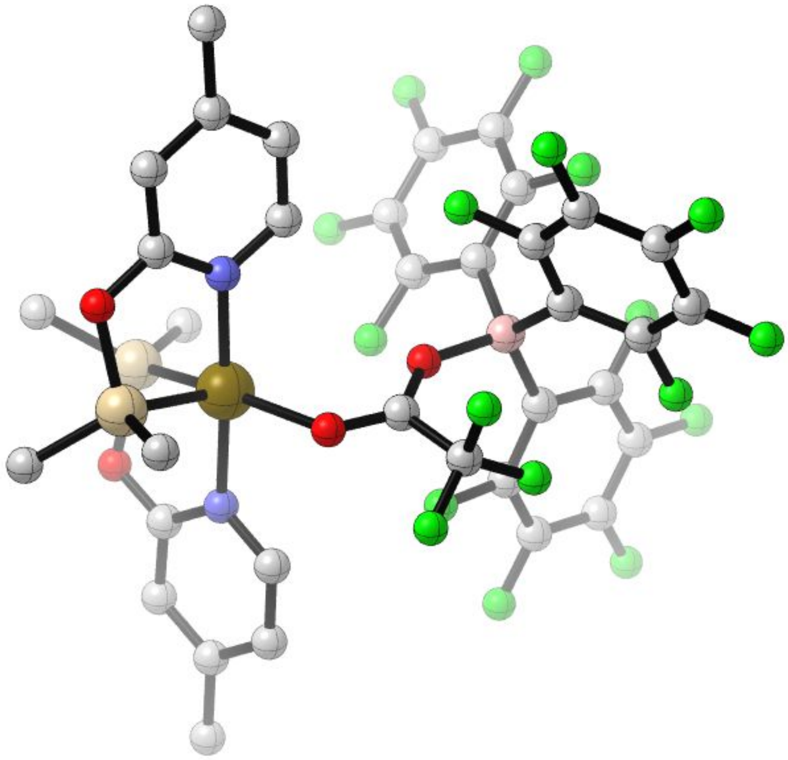

A

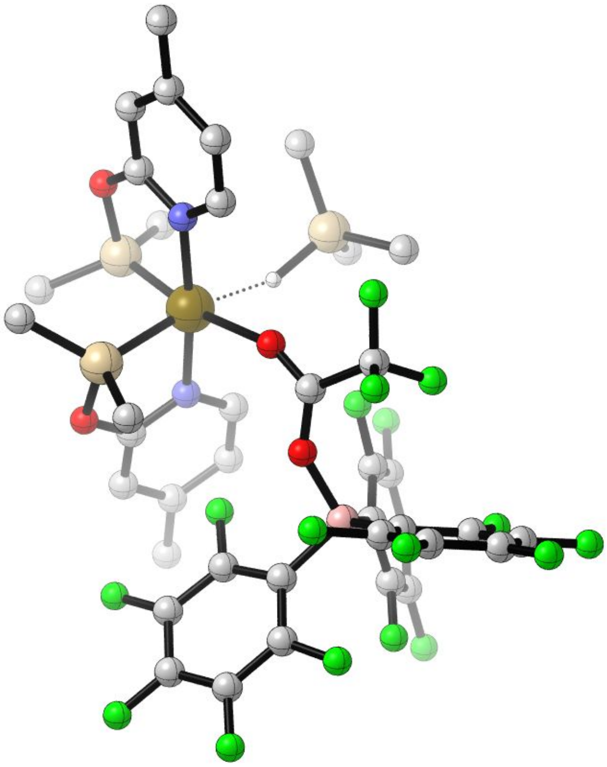

B

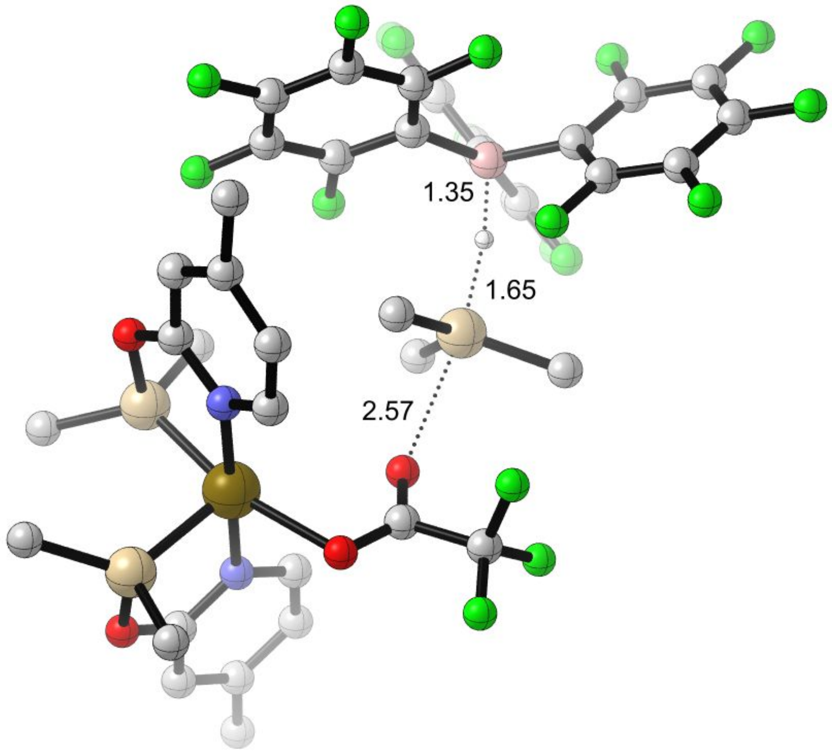

TSBC

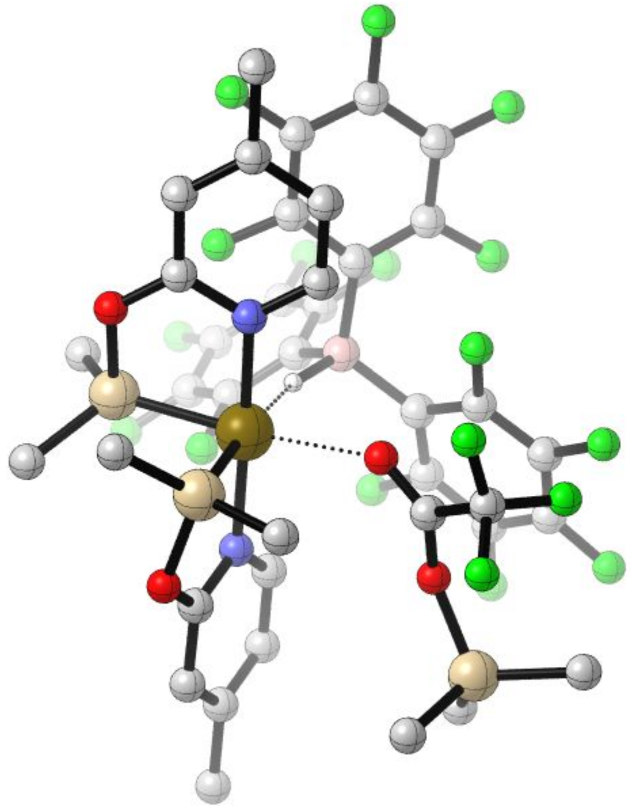

C

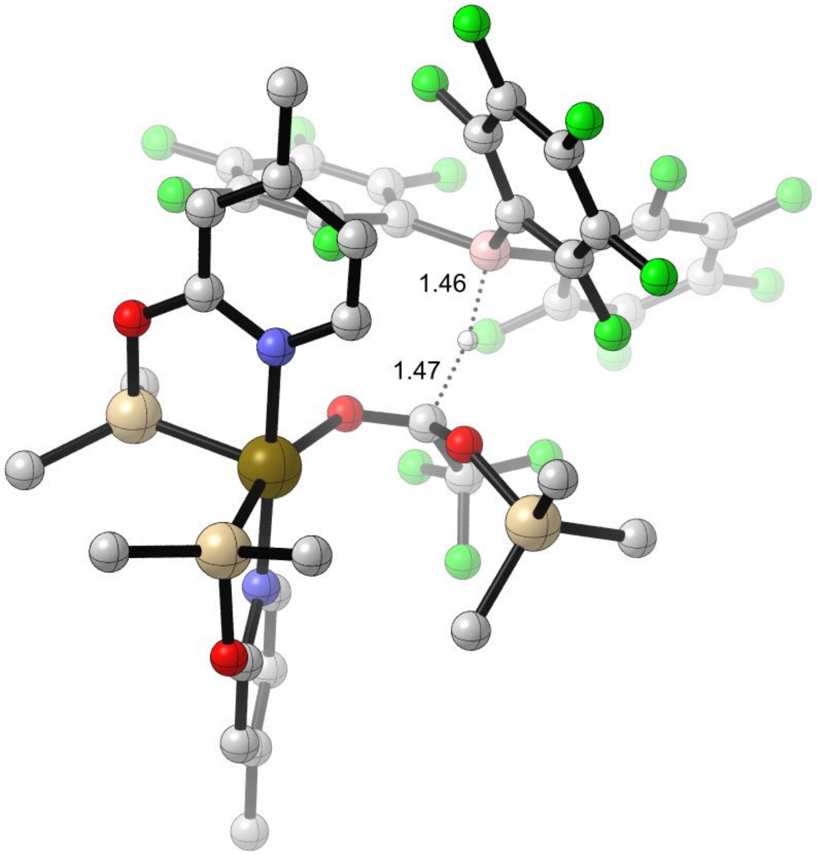

TSCD’

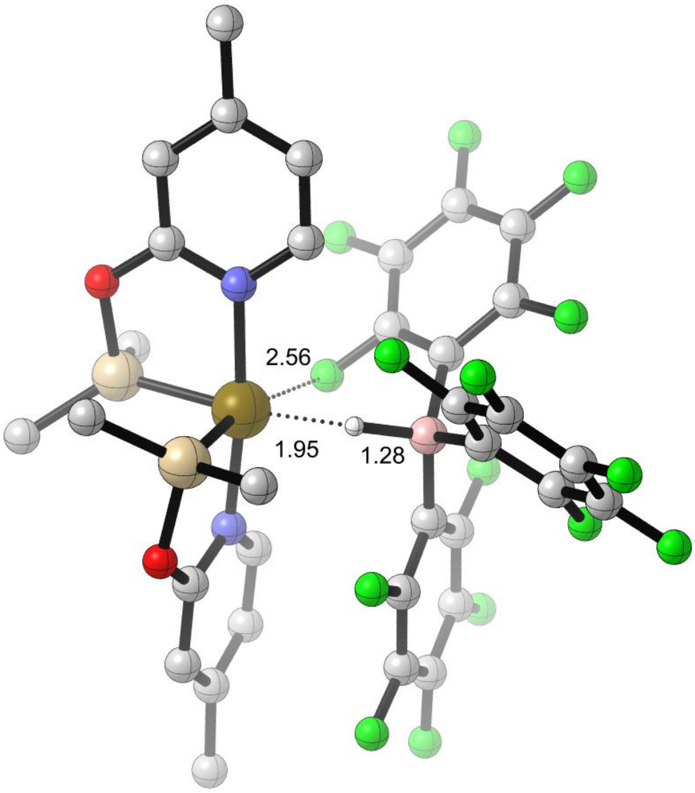

D

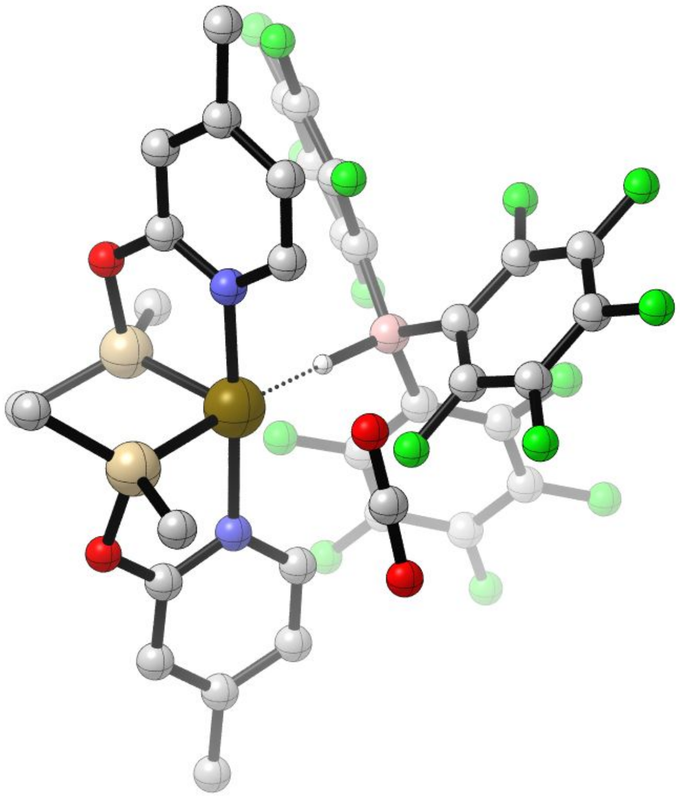

E

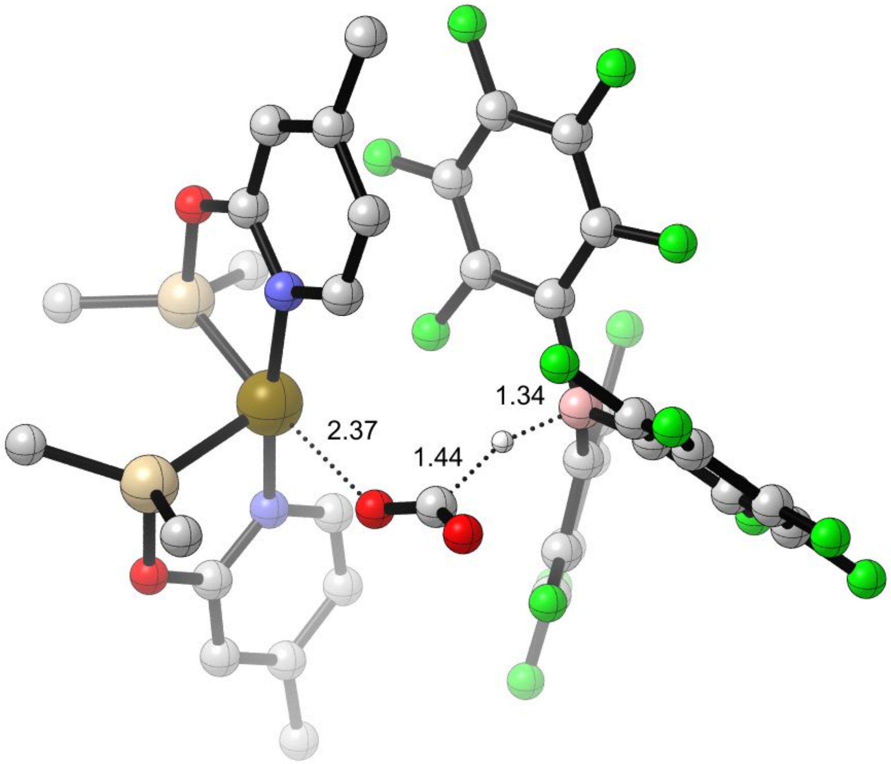

TSEF

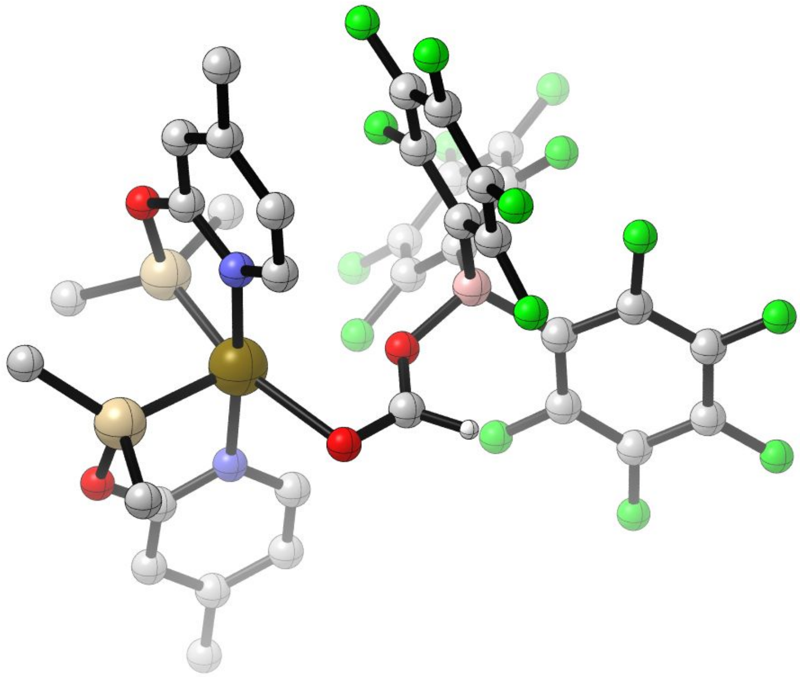

F

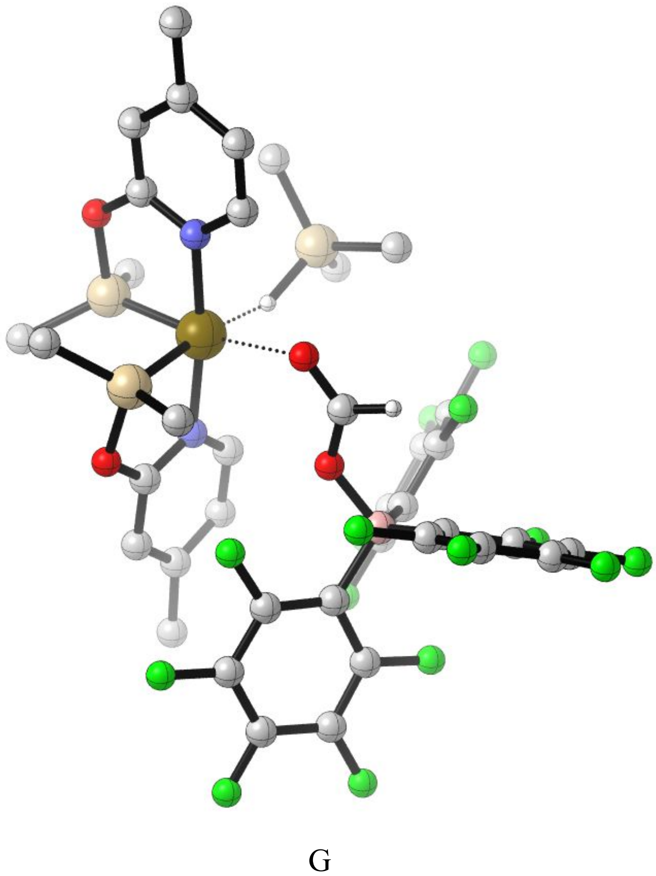

G

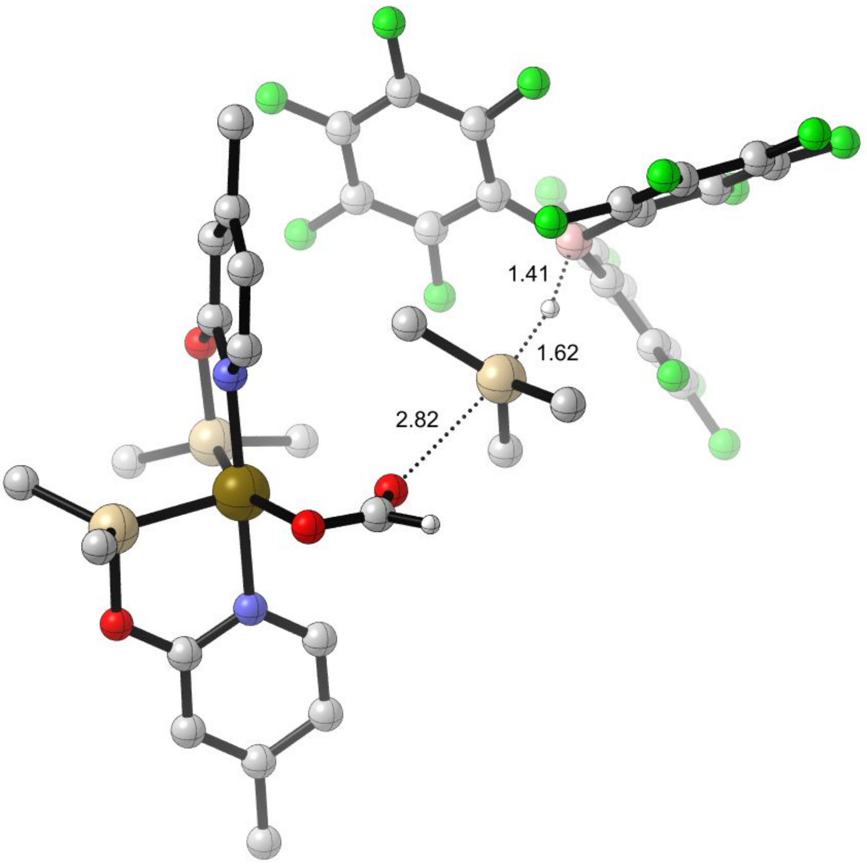

TSGH

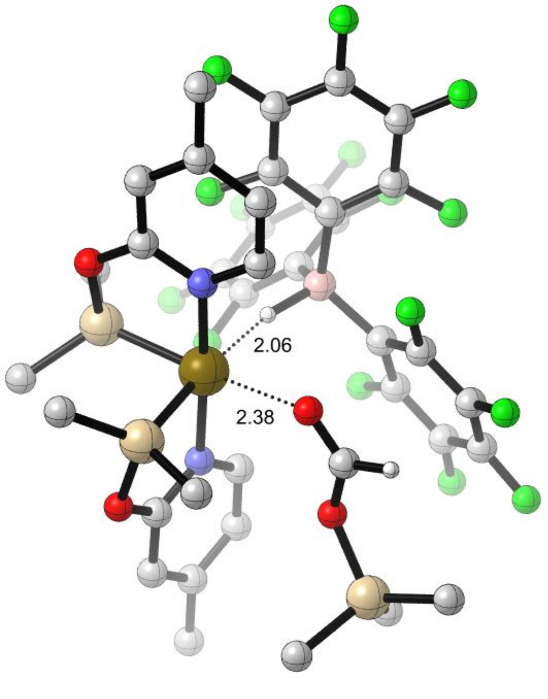

H

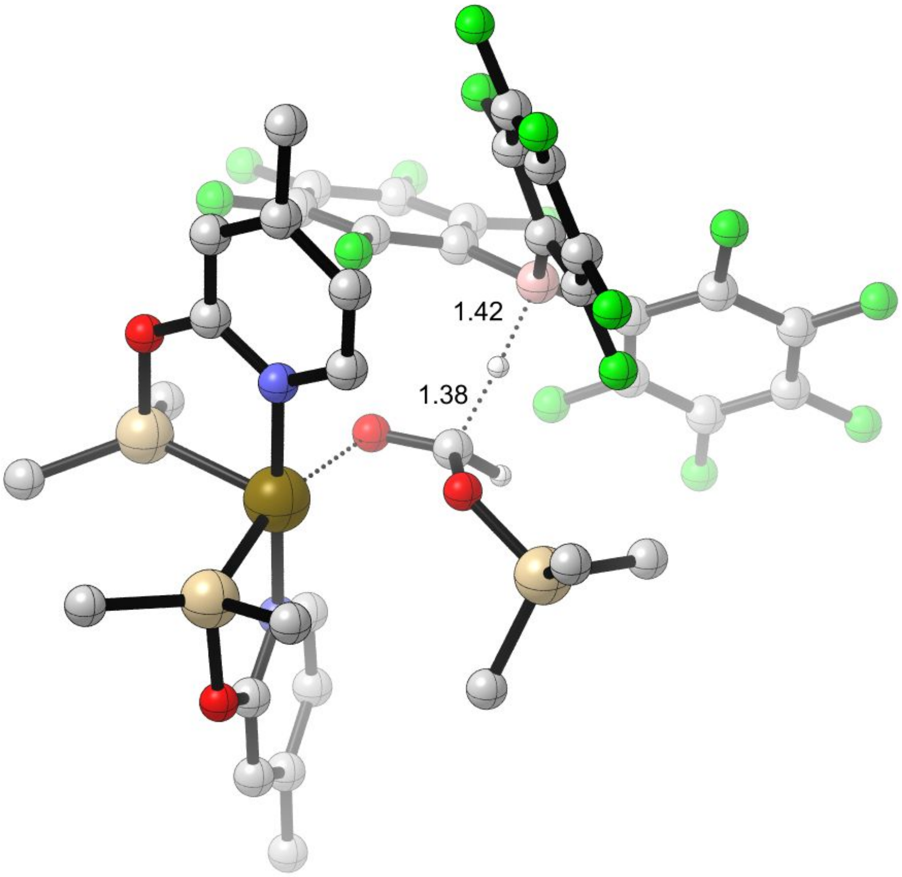

TSHI

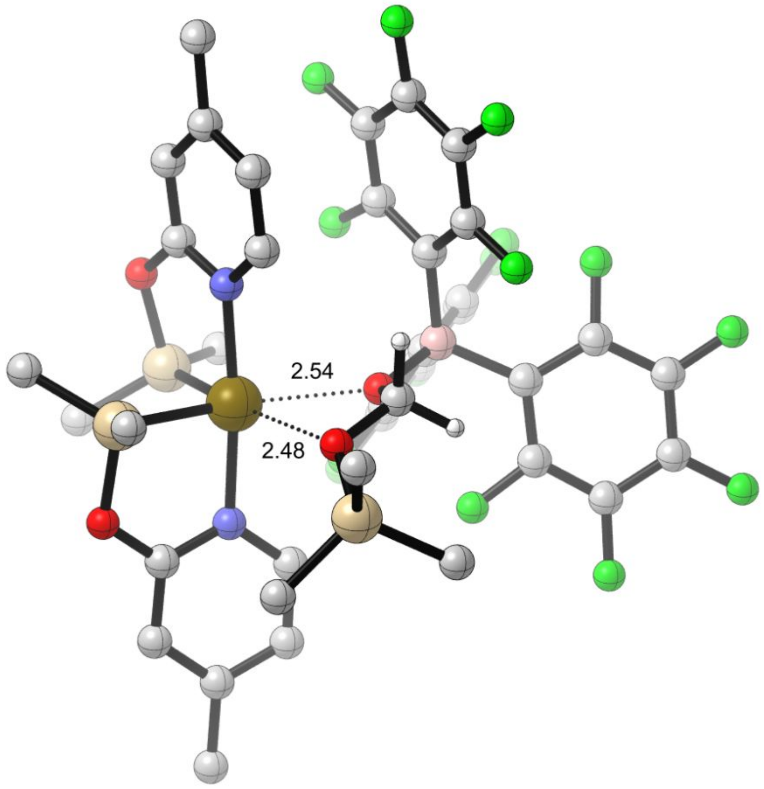

I

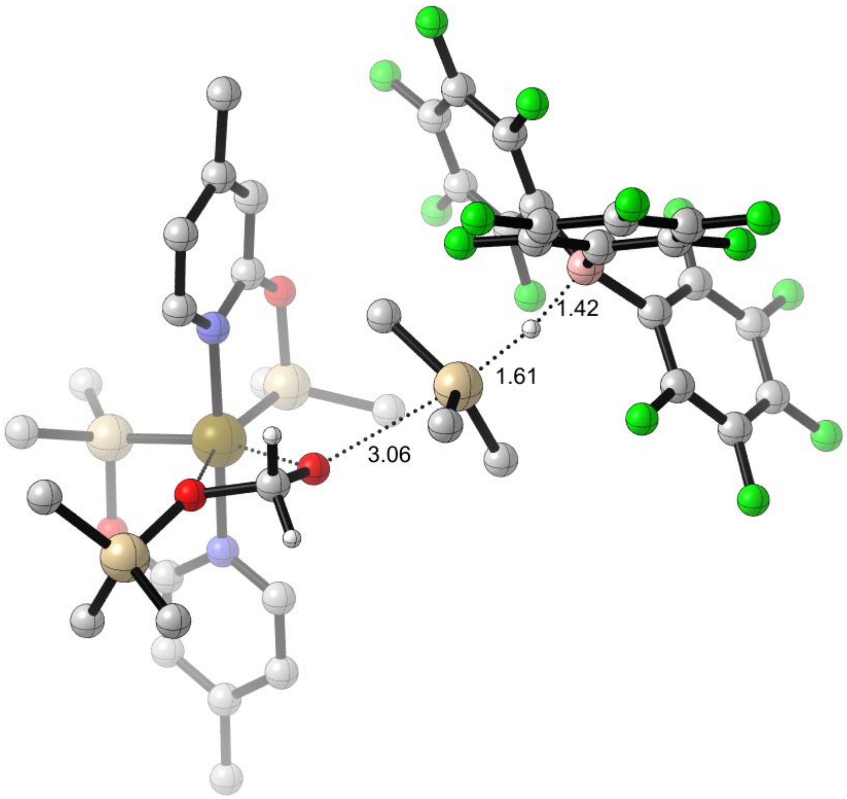

TSIJ

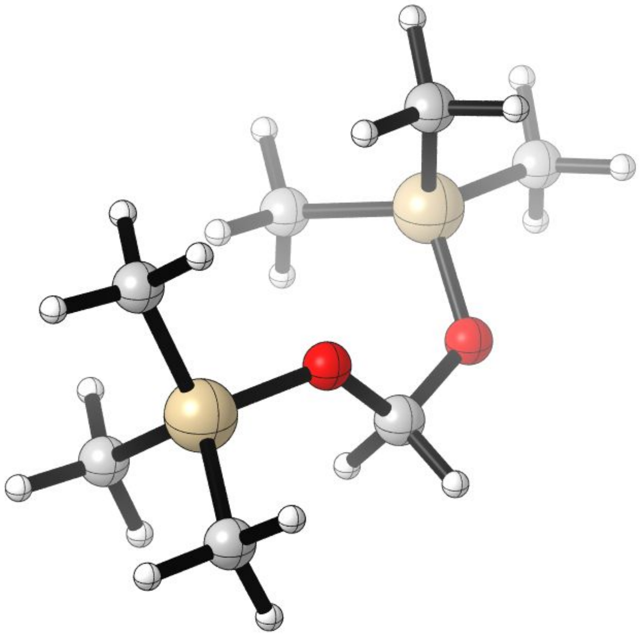

Bis(silyl)acetal

Table S3. List of Cartesian coordinates (xyz, in Å) for all DFT optimized structures.

88

A

77	2.456878	-0.276589	-0.234346
7	3.091836	1.702513	-0.414679
6	2.506530	2.575573	-1.272959
6	4.160203	2.124224	0.321987
6	2.926439	3.881239	-1.412113
1	1.679799	2.177813	-1.856329
6	4.633538	3.444009	0.211188
6	4.020184	4.345521	-0.650733
1	2.402076	4.538684	-2.106821
1	5.485185	3.726253	0.830635
7	1.925947	-2.281805	-0.043145
6	0.752373	-2.672026	0.507109
6	2.803043	-3.230750	-0.467414
6	0.418806	-3.999488	0.682598
1	0.071544	-1.871948	0.788814
6	2.524853	-4.595619	-0.298240
6	1.325756	-5.001947	0.282148
1	-0.551253	-4.250000	1.112885
1	3.266369	-5.312713	-0.651348
14	4.023283	-1.174229	-1.615302
14	3.916225	-0.237927	1.538401
8	4.749615	1.277995	1.139069
8	3.924553	-2.837897	-1.051361
6	3.433805	-1.219901	-3.396462
1	4.133968	-1.804826	-4.015698
1	2.430322	-1.662297	-3.469765
1	3.375119	-0.197953	-3.803198
6	5.817320	-0.642095	-1.575139
1	6.177383	-0.356063	-0.579280
1	6.456940	-1.449006	-1.969249
1	5.927991	0.230663	-2.239382
6	5.247211	-1.526339	1.849949
1	6.012094	-1.121782	2.533105
1	4.774050	-2.386792	2.352074
1	5.741901	-1.901564	0.945461
6	3.117202	0.144822	3.194101
1	2.624537	-0.746641	3.609385
1	3.892530	0.472399	3.906968
1	2.355168	0.932347	3.115101
6	4.490624	5.767111	-0.772497
1	4.744115	6.005986	-1.817973
1	3.691720	6.463490	-0.469185
1	5.373103	5.960621	-0.147447
6	0.990587	-6.455312	0.463755
1	0.824912	-6.684934	1.528916
1	0.057756	-6.705987	-0.066796
1	1.789736	-7.109284	0.088764
8	0.885023	-0.003682	-1.932949
8	-0.839313	-0.015651	-0.565092
6	-0.313446	0.167867	-1.706584
5	-2.222823	0.247340	0.099796
6	-2.423539	1.851149	0.340691

6	-3.526452	2.275983	1.088749
6	-1.540086	2.864588	-0.025649
6	-3.759848	3.604696	1.438921
6	-1.724049	4.209116	0.305178
6	-2.843201	4.582776	1.045866
6	-3.327879	-0.511469	-0.824074
6	-3.164124	-1.877920	-1.067799
6	-4.422297	0.077612	-1.458608
6	-4.013668	-2.632090	-1.871677
6	-5.305236	-0.638918	-2.272831
6	-5.098732	-2.001244	-2.482326
6	-2.053797	-0.418227	1.593996
6	-0.927714	-0.127350	2.371845
6	-3.019660	-1.210206	2.223633
6	-0.745626	-0.598552	3.672560
6	-2.871735	-1.713258	3.519846
6	-1.727162	-1.402990	4.251042
9	-0.424708	2.601105	-0.745900
9	-0.836686	5.128677	-0.079533
9	-3.039352	5.857337	1.372014
9	-4.834010	3.948613	2.145801
9	-4.430318	1.373169	1.494446
9	0.062872	0.646073	1.873294
9	0.344695	-0.288718	4.376325
9	-1.570016	-1.864995	5.487826
9	-3.818574	-2.473948	4.064281
9	-4.160270	-1.534466	1.607526
9	-4.672192	1.386137	-1.338141
9	-6.331810	-0.027597	-2.861456
9	-2.135153	-2.538262	-0.498260
9	-3.803461	-3.935231	-2.061779
9	-5.926038	-2.694600	-3.258785
6	-1.157830	0.592285	-2.943218
9	-2.126999	1.458844	-2.644735
9	-1.714973	-0.495984	-3.493121
9	-0.366731	1.162292	-3.852133

102
B

77	2.674204	-0.138311	-0.224296
8	-1.276879	0.715025	-0.453247
8	0.828274	1.397848	-0.325780
6	-0.381445	1.578105	-0.213075
6	-0.807165	3.017142	0.210502
9	-1.496349	2.998651	1.356700
9	-1.550221	3.598549	-0.735601
9	0.271188	3.794182	0.394532
7	3.903997	1.494513	-0.687940
6	3.437549	2.759090	-0.835327
6	5.237800	1.266860	-0.837814
6	4.259135	3.826814	-1.135875
1	2.364539	2.874939	-0.688724
6	6.117875	2.318345	-1.151815
6	5.644005	3.615430	-1.306290
1	3.824620	4.822391	-1.239237
1	7.174909	2.073919	-1.259388
7	1.664294	-1.924742	0.180096

6	1.217932	-2.244892	1.418468
6	1.551628	-2.850246	-0.811870
6	0.652756	-3.465770	1.718594
1	1.321607	-1.470088	2.173250
6	1.013364	-4.122051	-0.556389
6	0.540617	-4.445068	0.708533
1	0.297961	-3.651368	2.733067
1	0.937814	-4.813280	-1.394759
14	2.343954	-0.856723	-2.389324
14	4.668483	-1.241506	-0.065257
8	5.711789	0.046761	-0.680709
8	1.960257	-2.537082	-2.026586
6	0.784284	-0.163735	-3.169414
1	0.570673	-0.685596	-4.117010
1	-0.073193	-0.283297	-2.494827
1	0.903869	0.910038	-3.384391
6	3.714319	-0.843205	-3.673523
1	4.721816	-0.954465	-3.251821
1	3.550610	-1.642727	-4.414879
1	3.673913	0.120677	-4.206977
6	5.087959	-2.796533	-1.031785
1	6.177819	-2.961360	-1.007977
1	4.609481	-3.651761	-0.526129
1	4.751724	-2.798330	-2.074877
6	5.275298	-1.587774	1.686736
1	4.616045	-2.336693	2.156230
1	6.297065	-2.000880	1.666387
1	5.278357	-0.693693	2.322819
1	2.579175	0.405874	1.660328
14	2.909353	1.249928	2.886701
6	4.559991	2.107077	2.595492
1	5.366611	1.410905	2.324536
1	4.469135	2.851177	1.789352
1	4.866227	2.640339	3.510693
6	2.944668	0.025661	4.312360
1	3.670345	-0.784148	4.145247
1	3.214090	0.538448	5.250620
1	1.948129	-0.422732	4.450147
6	1.557343	2.523648	3.119761
1	1.730597	3.072644	4.060523
1	1.546571	3.250792	2.296729
1	0.566809	2.051323	3.172851
6	-0.101176	-5.772274	0.996524
1	0.364593	-6.255941	1.869888
1	-1.168409	-5.633462	1.236822
1	-0.030945	-6.453906	0.137925
6	6.561706	4.758054	-1.638405
1	6.498781	5.544129	-0.868560
1	7.607960	4.432092	-1.715908
1	6.271978	5.222550	-2.595108
6	-3.726295	1.563849	-0.088954
6	-3.934417	2.244186	-1.292095
6	-4.411666	2.077251	1.012847
6	-4.772399	3.347604	-1.421429
6	-5.266330	3.180930	0.931619
6	-5.447117	3.819194	-0.293761

6	-3.076372	-0.857471	-1.191755
6	-4.265850	-0.980543	-1.916034
6	-2.136346	-1.859543	-1.441339
6	-4.479940	-1.981686	-2.868951
6	-2.298931	-2.871094	-2.386663
6	-3.490617	-2.935255	-3.107098
6	-2.635386	-0.496354	1.380506
6	-3.703644	-1.315081	1.765163
6	-1.541134	-0.530845	2.239350
6	-3.679982	-2.149359	2.882910
6	-1.467018	-1.353015	3.363903
6	-2.535081	-2.185073	3.682032
9	-3.291642	1.839954	-2.398338
9	-4.928741	3.959821	-2.595365
9	-6.250701	4.875182	-0.387733
9	-5.897720	3.633161	2.015501
9	-4.271182	1.537240	2.232190
9	-4.830056	-1.313815	1.041238
9	-0.450268	0.239752	2.024784
9	-0.351309	-1.385053	4.110314
9	-2.464273	-2.999836	4.732602
9	-4.723105	-2.919065	3.185815
9	-5.284707	-0.135274	-1.728935
9	-5.628979	-2.042594	-3.540200
9	-3.681145	-3.894659	-4.009302
9	-1.341653	-3.782511	-2.589045
9	-0.989632	-1.885084	-0.741063
5	-2.728125	0.285437	-0.059378

102

TSBC

7	2.616680	-2.008328	-0.142300
6	2.318108	-2.789130	0.924702
6	2.272866	-2.442025	-1.390120
6	1.630454	-3.980088	0.808077
1	2.636182	-2.395940	1.890956
6	1.556921	-3.639200	-1.562651
6	1.206912	-4.418519	-0.464785
1	1.404769	-4.558463	1.704974
1	1.269335	-3.911496	-2.576959
7	4.333747	1.762272	0.259967
6	3.598484	2.859001	0.564467
6	5.665698	1.903416	0.030083
6	4.146010	4.124959	0.624397
1	2.544413	2.669361	0.771832
6	6.275173	3.168883	0.076481
6	5.523040	4.301316	0.370089
1	3.506676	4.973990	0.871062
1	7.346216	3.222900	-0.120247
14	5.650354	-0.767824	-0.062469
14	3.200208	-0.063377	-2.198430
8	2.607564	-1.717257	-2.432853
8	6.378630	0.828478	-0.247753
6	6.214198	-1.408900	1.610956
1	7.315218	-1.415643	1.671943
1	5.804571	-0.802431	2.431133
1	5.855903	-2.442969	1.746604

6	6.389554	-1.830215	-1.419713
1	6.664472	-1.227691	-2.295412
1	7.294102	-2.337275	-1.047555
1	5.673709	-2.602072	-1.743706
6	4.538538	0.163195	-3.503260
1	5.071663	-0.778139	-3.694330
1	4.093408	0.503045	-4.451781
1	5.277070	0.914993	-3.181831
6	1.734558	1.009781	-2.697694
1	1.980540	2.078153	-2.580238
1	1.479066	0.826794	-3.754300
1	0.839274	0.795590	-2.101387
6	6.144996	5.668341	0.426707
1	6.008981	6.116004	1.424636
1	7.220753	5.636581	0.206152
1	5.662389	6.345136	-0.297041
6	0.374556	-5.659650	-0.625362
1	-0.698306	-5.404135	-0.619195
1	0.576840	-6.159008	-1.583178
1	0.550437	-6.371199	0.194092
77	3.478307	-0.124652	0.081543
8	1.370491	0.928108	1.805926
14	-1.091380	0.730103	1.094714
6	-1.673249	1.118345	2.824977
1	-0.957256	1.797331	3.307253
1	-2.654442	1.612489	2.781328
1	-1.757279	0.206479	3.430279
6	-0.434525	-0.986173	0.749480
1	-1.246567	-1.698086	0.569076
1	0.239518	-0.984513	-0.114700
1	0.123456	-1.329369	1.627510
6	2.125870	0.369933	2.627822
6	-0.560734	2.202020	0.065350
1	-1.206996	2.342760	-0.809941
1	-0.602837	3.108773	0.686745
1	0.468824	2.059150	-0.283215
1	-2.578840	0.508651	0.408652
5	-3.665323	0.142969	-0.308738
6	-3.961353	1.488202	-1.142773
6	-3.879468	2.737637	-0.526193
6	-4.336316	1.503918	-2.487387
6	-4.118115	3.941556	-1.187409
6	-4.590020	2.683094	-3.190977
6	-4.479672	3.910303	-2.535224
6	-3.093379	-1.112224	-1.152597
6	-2.003847	-0.927790	-2.009820
6	-3.519272	-2.436785	-1.015886
6	-1.342533	-1.958918	-2.668665
6	-2.877502	-3.508207	-1.648294
6	-1.791818	-3.266490	-2.486591
6	-4.722669	-0.160637	0.871286
6	-4.364734	-0.914932	1.991140
6	-6.034246	0.323474	0.874563
6	-5.212477	-1.157931	3.068271
6	-6.924784	0.099987	1.929436
6	-6.509829	-0.642236	3.034649

```
   9   -1.495384    0.306519   -2.177036
   9   -0.279002   -1.716525   -3.434055
   9   -1.170768   -4.281338   -3.088874
   9   -3.285741   -4.761941   -1.448267
   9   -4.565787   -2.752259   -0.246594
   9   -4.480382    0.356642   -3.164445
   9   -4.942681    2.648353   -4.474375
   9   -4.716822    5.042587   -3.190318
   9   -3.546444    2.816330    0.775948
   9   -4.008879    5.107047   -0.551791
   9   -3.135225   -1.459517    2.057896
   9   -4.804021   -1.874335    4.113953
   9   -7.343202   -0.862076    4.046004
   9   -8.163942    0.584023    1.884487
   9   -6.509262    1.027618   -0.160502
   8    3.218685   -0.188902    2.376576
   6    1.646235    0.283030    4.097288
   9    1.123703    1.444791    4.513234
   9    2.622340   -0.056006    4.935461
   9    0.681213   -0.659255    4.182001
102
C
  77   -0.266873   -1.178051    1.033005
   8   -3.345964    0.127382   -1.102561
   8   -1.497017   -1.103805   -1.047536
   6   -2.591686   -0.850726   -1.496969
   7    1.126154   -2.443957    0.161358
   6    1.203218   -2.609051   -1.181139
   6    1.998053   -3.110461    0.966049
   6    2.148812   -3.420913   -1.772482
   1    0.480422   -2.045465   -1.767572
   6    2.979220   -3.950015    0.413323
   6    3.084257   -4.100079   -0.964996
   1    2.182002   -3.499469   -2.859863
   1    3.676958   -4.430828    1.098691
   7   -1.754005   -0.028926    1.961361
   6   -1.837827    1.312037    1.812445
   6   -2.673804   -0.655220    2.749097
   6   -2.793569    2.081964    2.443734
   1   -1.089158    1.763498    1.174080
   6   -3.675266    0.077595    3.411395
   6   -3.747288    1.460460    3.274649
   1   -2.787064    3.161752    2.288738
   1   -4.381495   -0.478601    4.028484
  14   -1.675846   -2.880088    1.690084
  14    1.010377   -1.559769    2.922963
   8    1.921504   -2.937165    2.269633
   8   -2.627116   -1.967167    2.874411
   6   -3.016985   -3.396807    0.463792
   1   -3.644675   -4.175951    0.927144
   1   -3.677131   -2.558258    0.202772
   1   -2.584868   -3.810138   -0.459255
   6   -1.084954   -4.482377    2.472943
   1   -0.117083   -4.409261    2.982280
   1   -1.839912   -4.858892    3.182861
   1   -0.987830   -5.226375    1.664685
```

6	0.198827	-2.120568	4.531506
1	0.947605	-2.644266	5.148943
1	-0.094125	-1.207699	5.077022
1	-0.691075	-2.752107	4.443231
6	2.356047	-0.397671	3.536282
1	1.921204	0.393584	4.162742
1	3.034580	-1.003095	4.161091
1	2.943045	0.066920	2.738969
1	0.848876	0.358481	0.322242
14	-4.997695	0.727725	-1.174974
6	-5.557034	0.756748	-2.953924
1	-5.730496	-0.244025	-3.371547
1	-6.500069	1.325315	-3.014973
1	-4.812321	1.277405	-3.575237
6	-4.882608	2.445288	-0.470792
1	-5.901857	2.839125	-0.321108
1	-4.369418	2.435818	0.500513
1	-4.351038	3.136171	-1.138435
6	-5.952434	-0.421942	-0.053211
1	-5.987384	-1.445563	-0.453735
1	-5.486139	-0.450539	0.945156
1	-6.988460	-0.064516	0.065618
6	-4.806962	2.270474	3.966534
1	-4.349519	3.019919	4.632442
1	-5.413048	2.824526	3.230880
1	-5.478768	1.639946	4.565024
6	4.199103	-4.888525	-1.589904
1	3.828229	-5.535227	-2.399997
1	4.931658	-4.193260	-2.032685
1	4.725511	-5.509267	-0.851901
5	1.389216	1.233663	-0.444469
6	2.570029	0.463744	-1.277914
6	3.436284	-0.398362	-0.602535
6	2.882405	0.645134	-2.630495
6	4.471574	-1.108197	-1.208204
6	3.885364	-0.067856	-3.292330
6	4.680490	-0.962548	-2.575901
6	0.150587	1.837148	-1.327166
6	-0.348412	1.208486	-2.475514
6	-0.560410	2.983583	-0.954091
6	-1.409828	1.699520	-3.234181
6	-1.655298	3.490503	-1.658194
6	-2.082996	2.842656	-2.810622
6	2.106952	2.325341	0.536385
6	3.103569	3.180098	0.055250
6	1.830190	2.462066	1.895819
6	3.818435	4.066243	0.865211
6	2.521775	3.320918	2.749122
6	3.531080	4.130264	2.229050
9	3.293612	-0.589417	0.720991
9	5.238994	-1.945635	-0.503210
9	5.631916	-1.670080	-3.188355
9	4.107126	0.118014	-4.593815
9	2.228477	1.548656	-3.370363
9	0.829665	1.762128	2.461879
9	2.220492	3.374853	4.049026

9	4.202555	4.963499	3.020940
9	4.764442	4.851098	0.349718
9	3.411998	3.187816	-1.250385
9	-0.251474	3.660186	0.167765
9	0.173795	0.046884	-2.904105
9	-1.823981	1.061677	-4.330607
9	-3.160050	3.276612	-3.472928
9	-2.320239	4.558976	-1.210097
6	-3.137978	-1.756924	-2.636704
9	-2.340725	-2.799569	-2.829360
9	-3.245159	-1.081011	-3.780259
9	-4.364950	-2.214040	-2.313714

102

TSCD'

77	2.597694	0.072669	-0.003857
8	0.567972	-0.787060	0.693420
8	0.141353	-0.865705	-1.504748
6	-0.178876	-1.220428	-0.228358
7	1.868145	2.002039	-0.219687
6	1.138786	2.375509	-1.296037
6	2.107512	2.908263	0.759738
6	0.649821	3.655338	-1.453017
1	0.939225	1.591312	-2.024319
6	1.613673	4.218840	0.668308
6	0.878363	4.614801	-0.442993
1	0.076981	3.909594	-2.344391
1	1.818760	4.896714	1.496778
7	3.398153	-1.843895	0.183627
6	2.812463	-2.801026	0.944684
6	4.553943	-2.139712	-0.472585
6	3.319425	-4.078937	1.053437
1	1.901225	-2.493042	1.455854
6	5.107386	-3.430947	-0.405509
6	4.495986	-4.422156	0.353636
1	2.796674	-4.809972	1.671852
1	6.025834	-3.615364	-0.963224
14	4.547262	0.470730	-1.142270
14	3.304716	0.864924	2.011118
8	2.820815	2.534506	1.806675
8	5.152573	-1.200765	-1.172878
6	4.237948	0.906067	-2.953659
1	5.158006	0.768877	-3.545348
1	3.443366	0.301205	-3.410694
1	3.940416	1.965564	-3.026449
6	6.045506	1.478328	-0.613753
1	6.555688	1.031597	0.249865
1	6.764961	1.534964	-1.446764
1	5.750723	2.506507	-0.348192
6	5.074529	0.946445	2.618998
1	5.581853	-0.020390	2.471747
1	5.653572	1.728764	2.112708
1	5.067531	1.162825	3.700063
6	2.253578	0.067957	3.348171
1	2.739554	-0.868936	3.667905
1	2.171403	0.735516	4.220174
1	1.248128	-0.174941	2.980871

6	5.060621	-5.812251	0.437662
1	4.336043	-6.547607	0.051235
1	5.991781	-5.910467	-0.137021
1	5.267245	-6.086350	1.484877
6	0.314752	6.001142	-0.569161
1	0.592562	6.633975	0.284690
1	0.670532	6.484496	-1.493485
1	-0.783345	5.955931	-0.630492
1	-1.524316	-0.665377	-0.039268
5	-2.720871	0.089777	0.337109
6	-3.937578	-0.943150	0.114090
6	-3.993762	-2.166263	0.795588
6	-5.017716	-0.709376	-0.749060
6	-4.998105	-3.113566	0.618194
6	-6.050527	-1.628709	-0.955246
6	-6.040311	-2.840880	-0.268893
6	-2.323353	0.488228	1.845224
6	-1.186291	1.272119	2.073715
6	-3.043674	0.162950	3.002850
6	-0.751112	1.675608	3.331042
6	-2.626985	0.519687	4.289585
6	-1.465650	1.272566	4.457682
6	-2.648510	1.297232	-0.733822
6	-2.810537	2.645472	-0.399215
6	-2.474197	1.044364	-2.096202
6	-2.758923	3.675195	-1.339623
6	-2.421019	2.040996	-3.068013
6	-2.534810	3.374804	-2.679716
9	-2.412636	-0.224450	-2.531997
9	-2.249386	1.739204	-4.359058
9	-2.416677	4.351494	-3.577975
9	-2.884107	4.951160	-0.959727
9	-3.050820	3.022099	0.862347
9	-0.485497	1.740656	1.033150
9	0.334421	2.443820	3.463159
9	-1.056536	1.623037	5.673415
9	-3.344234	0.165229	5.354692
9	-4.212927	-0.482033	2.938374
9	-3.037746	-2.476631	1.676776
9	-4.979638	-4.263986	1.290005
9	-7.014454	-3.726127	-0.453081
9	-5.136970	0.441725	-1.426444
9	-7.049194	-1.347505	-1.791742
14	0.454278	-1.574139	-3.049704
6	2.129011	-2.422684	-2.990772
1	2.933761	-1.730262	-2.709148
1	2.141887	-3.268529	-2.290851
1	2.365918	-2.806332	-3.997410
6	0.545625	-0.106441	-4.205529
1	-0.412638	0.423506	-4.275087
1	1.324097	0.609875	-3.903872
1	0.805647	-0.462570	-5.216232
6	-0.876318	-2.772475	-3.587961
1	-0.695532	-3.031490	-4.644980
1	-0.861414	-3.701974	-3.003006
1	-1.876125	-2.324027	-3.511310

6	-0.575050	-2.723518	-0.084748
9	0.373829	-3.487132	-0.688560
9	-0.607495	-3.090448	1.189164
9	-1.736070	-3.029042	-0.668428

82

D

77	-0.786143	-1.332424	0.304412
7	1.026936	-2.307459	0.085656
6	1.611086	-2.449484	-1.128780
6	1.606326	-2.881979	1.172605
6	2.807994	-3.110423	-1.301251
1	1.085311	-1.987227	-1.963039
6	2.848175	-3.531060	1.059582
6	3.476161	-3.639149	-0.175716
1	3.247273	-3.177782	-2.297075
1	3.298463	-3.925364	1.970453
7	-2.752374	-0.587616	0.449577
6	-3.057826	0.733952	0.435851
6	-3.778420	-1.483534	0.539664
6	-4.349331	1.210891	0.521392
1	-2.213171	1.409475	0.363688
6	-5.111800	-1.052363	0.647875
6	-5.421278	0.302605	0.639821
1	-4.520214	2.288243	0.505634
1	-5.882509	-1.820006	0.720834
14	-1.932628	-3.287548	-0.068122
14	-0.437384	-1.778618	2.517273
8	0.980714	-2.819984	2.329441
8	-3.515288	-2.776589	0.513506
6	-2.167943	-3.548541	-1.922124
1	-2.921344	-4.330658	-2.113702
1	-2.484412	-2.623890	-2.427979
1	-1.217854	-3.871156	-2.379302
6	-1.537940	-4.946168	0.710642
1	-1.219181	-4.880942	1.758799
1	-2.408189	-5.619598	0.644731
1	-0.715000	-5.403529	0.136778
6	-1.706056	-2.706823	3.544265
1	-1.217216	-3.164792	4.419992
1	-2.435966	-1.969498	3.918017
1	-2.262041	-3.481235	3.002283
6	0.195750	-0.363793	3.571857
1	-0.607229	0.349525	3.804393
1	0.582399	-0.782229	4.516466
1	1.007456	0.177563	3.070348
1	0.054374	0.428079	0.361303
6	4.845486	-4.236818	-0.320146
1	4.873895	-4.977602	-1.134484
1	5.561552	-3.439434	-0.578131
1	5.184100	-4.716434	0.608195
6	-6.837726	0.791957	0.744006
1	-6.959912	1.438716	1.628072
1	-7.103688	1.399099	-0.136502
1	-7.552449	-0.038770	0.821018
5	0.635411	1.342736	-0.322788
6	2.034416	0.697889	-0.839751

6	2.915057	0.146084	0.095962
6	2.481001	0.660942	-2.161780
6	4.146847	-0.412111	-0.233035
6	3.704162	0.099535	-2.542521
6	4.541833	-0.440198	-1.569030
6	0.924258	2.512352	0.771964
6	0.126338	2.711063	1.896739
6	2.015409	3.380307	0.665230
6	0.398829	3.652518	2.889679
6	2.327177	4.344155	1.625537
6	1.513646	4.478087	2.752138
6	-0.452097	1.748612	-1.472843
6	-0.939152	3.040495	-1.694774
6	-1.073965	0.767936	-2.245002
6	-1.997944	3.320802	-2.568913
6	-2.134760	0.983445	-3.114821
6	-2.605164	2.287485	-3.281046
9	-0.677905	-0.533369	-2.121123
9	-2.707433	-0.030665	-3.765159
9	-3.618472	2.537920	-4.104354
9	-2.430242	4.569813	-2.725330
9	-0.421858	4.094463	-1.053824
9	1.735582	1.174707	-3.151344
9	4.067248	0.060504	-3.824843
9	5.687843	-1.031085	-1.914044
9	2.569502	0.103314	1.395511
9	4.921356	-0.975263	0.697727
9	2.818718	3.326334	-0.407653
9	3.383787	5.141520	1.474682
9	1.795009	5.389540	3.679810
9	-0.396297	3.769113	3.954501
9	-0.990726	1.975916	2.076049

85
E

77	0.964463	-1.313556	-0.140395
8	0.668097	-2.331362	3.758503
6	1.630339	-1.740684	4.045509
7	-0.775516	-2.431971	0.015913
6	-1.411868	-2.584332	1.199070
6	-1.275375	-3.050604	-1.085800
6	-2.583353	-3.303186	1.324418
1	-0.948687	-2.095272	2.055172
6	-2.480333	-3.763123	-1.025947
6	-3.165463	-3.882391	0.180001
1	-3.061694	-3.381236	2.301373
1	-2.860896	-4.196780	-1.950867
7	2.840370	-0.378271	-0.208812
6	3.029839	0.925403	0.102508
6	3.918711	-1.115283	-0.604153
6	4.236410	1.570059	-0.070689
1	2.161933	1.441874	0.502194
6	5.164261	-0.505874	-0.827093
6	5.337715	0.852942	-0.581993
1	4.317676	2.629327	0.175376
1	5.981295	-1.138019	-1.175772
14	2.353110	-3.148525	0.012430

14	0.671215	-1.753856	-2.381073
8	-0.599597	-2.961597	-2.217623
8	3.774373	-2.418295	-0.755982
6	2.992875	-3.555300	1.738309
1	3.811535	-4.289878	1.656596
1	3.389104	-2.664247	2.247049
1	2.197561	-3.995736	2.358395
6	1.999170	-4.785263	-0.835501
1	1.394945	-4.698910	-1.747065
1	2.943437	-5.299829	-1.078544
1	1.446749	-5.419925	-0.122910
6	2.048913	-2.482451	-3.427181
1	1.601065	-2.913303	-4.338211
1	2.699980	-1.648898	-3.739087
1	2.680155	-3.236882	-2.946949
6	-0.148643	-0.461150	-3.454056
1	0.513546	0.387294	-3.667153
1	-0.417383	-0.954791	-4.403140
1	-1.062770	-0.077443	-2.991617
1	-0.123747	0.373591	-0.239194
6	6.649702	1.540204	-0.830986
1	6.530627	2.319279	-1.601886
1	7.006541	2.043996	0.081590
1	7.423552	0.836448	-1.166776
6	-4.508208	-4.551366	0.242348
1	-4.712896	-4.963520	1.240930
1	-5.291569	-3.806565	0.019287
1	-4.597666	-5.356171	-0.501620
5	-0.926769	1.348788	0.006265
6	-0.021648	2.698909	-0.159248
6	-0.162480	3.841098	0.635565
6	0.988357	2.783567	-1.120794
6	0.663168	4.965303	0.523034
6	1.845153	3.871832	-1.263263
6	1.682692	4.976888	-0.427470
6	-1.413756	1.157983	1.551025
6	-2.684874	1.521245	2.007500
6	-0.549645	0.703516	2.543835
6	-3.080083	1.429951	3.342665
6	-0.883206	0.626985	3.896514
6	-2.169501	0.979346	4.299197
6	-2.116873	1.195092	-1.099369
6	-2.292623	2.014699	-2.219756
6	-3.015004	0.125477	-1.027625
6	-3.242459	1.764482	-3.217011
6	-3.960521	-0.179470	-2.002915
6	-4.075394	0.651480	-3.117732
9	1.200404	1.754683	-1.960424
9	-1.526313	3.095793	-2.421389
9	-3.001170	-0.688211	0.040914
9	-4.755093	-1.246294	-1.871724
9	-4.973752	0.391757	-4.063931
9	-3.346542	2.575482	-4.268884
9	2.830894	3.853943	-2.163942
9	2.492623	6.027443	-0.537013
9	0.484331	6.021088	1.315285

9	-1.128635	3.921745	1.561121
9	0.715756	0.307273	2.221361
9	-3.590870	2.017928	1.158202
9	-4.306898	1.785528	3.716943
9	-2.516914	0.902692	5.580650
9	0.008108	0.226309	4.811542
8	2.607617	-1.179973	4.322265

85

TSEF

77	-2.146787	-0.344285	-0.522633
7	-2.762307	1.615474	-0.293300
6	-2.305683	2.358206	0.742581
6	-3.475469	2.214138	-1.279166
6	-2.536310	3.713711	0.839009
1	-1.719554	1.820052	1.489659
6	-3.737380	3.594674	-1.236973
6	-3.258966	4.366205	-0.183268
1	-2.133650	4.274668	1.681823
1	-4.297104	4.032085	-2.063965
7	-1.354347	-2.272534	-0.627935
6	-0.156629	-2.521152	-1.219069
6	-1.944933	-3.269137	0.090011
6	0.513855	-3.716405	-1.084012
1	0.275666	-1.712891	-1.804427
6	-1.312484	-4.516448	0.246086
6	-0.062199	-4.750141	-0.313595
1	1.491814	-3.830215	-1.552589
1	-1.815033	-5.260647	0.863640
14	-3.726410	-1.385458	0.768262
14	-3.362926	-0.195992	-2.454350
8	-3.901576	1.474631	-2.284716
8	-3.101373	-3.037046	0.670218
6	-3.549487	-0.827031	2.559471
1	-4.002736	-1.565212	3.241266
1	-2.501073	-0.686045	2.850584
1	-4.081549	0.130709	2.687663
6	-5.561079	-1.596694	0.436338
1	-5.757476	-2.241645	-0.429673
1	-6.027698	-2.057937	1.322266
1	-6.047481	-0.622764	0.267700
6	-4.923268	-1.155128	-2.848114
1	-5.760393	-0.874717	-2.196740
1	-5.210638	-0.945065	-3.891481
1	-4.752801	-2.239325	-2.755317
6	-2.244008	-0.245144	-3.961793
1	-1.861082	-1.268703	-4.109381
1	-2.811479	0.039112	-4.863329
1	-1.384424	0.429467	-3.852855
1	1.433063	0.328876	-0.672253
6	-3.460232	5.853320	-0.129920
1	-3.976954	6.146268	0.798174
1	-2.480930	6.359282	-0.127095
1	-4.042281	6.220916	-0.985906
6	0.689354	-6.025961	-0.065558
1	1.101304	-6.432377	-1.002040
1	1.541263	-5.831568	0.606712

1	0.055076	-6.788949	0.405488
5	2.280379	0.416131	0.366183
6	1.756454	1.752836	1.107415
6	1.389612	2.883263	0.369738
6	1.538270	1.845004	2.484834
6	0.765658	3.997068	0.929043
6	0.924056	2.943819	3.091688
6	0.531098	4.023596	2.302545
6	3.655708	0.488214	-0.450509
6	3.927911	-0.465699	-1.437373
6	4.596287	1.512226	-0.325971
6	5.050413	-0.424973	-2.257799
6	5.737600	1.594916	-1.128734
6	5.962732	0.621410	-2.101645
6	1.939884	-0.969887	1.137186
6	2.848631	-2.016552	1.337205
6	0.657874	-1.219133	1.631620
6	2.492737	-3.237236	1.920821
6	0.273419	-2.392413	2.268860
6	1.197293	-3.427186	2.391134
9	-0.316525	-0.277947	1.488613
9	-0.970544	-2.560206	2.722683
9	0.831327	-4.593558	2.917305
9	3.376488	-4.230828	2.015738
9	4.127716	-1.904472	0.971471
9	1.897244	0.842944	3.300044
9	0.712477	2.969483	4.406722
9	-0.105394	5.062030	2.850177
9	1.612026	2.920181	-0.951602
9	0.363322	5.018276	0.170548
9	4.441771	2.473452	0.595701
9	6.610808	2.589032	-0.970873
9	7.044715	0.683841	-2.872617
9	5.263333	-1.361890	-3.181308
9	3.082981	-1.499895	-1.610707
6	0.752729	0.641541	-1.902902
8	-0.391373	0.868633	-1.564527
8	1.512790	0.544363	-2.804022

85

F

77	-2.139842	-0.025556	-0.387137
14	-2.828536	0.305100	1.777585
14	-4.247229	0.325759	-1.145590
8	-2.831670	2.082115	1.769415
8	-4.917228	-1.265388	-0.800108
8	-0.886890	-0.373989	-2.405504
8	0.514544	-0.432770	-0.716819
7	-1.704251	2.006625	-0.242608
7	-2.742149	-2.018988	-0.591717
6	-1.081058	2.675865	-1.244349
1	-0.765142	2.067576	-2.091179
6	-0.870525	4.038262	-1.214508
1	-0.358599	4.520815	-2.047758
6	-1.298829	4.784809	-0.094809
6	-1.950340	4.099137	0.922352
1	-2.316962	4.605859	1.815136

6	-2.165312	2.712455	0.829448
6	-1.015125	6.255302	0.008768
1	0.028081	6.405941	0.328215
1	-1.670362	6.744122	0.742978
1	-1.132791	6.755019	-0.964470
6	-1.552274	-0.055904	3.109322
1	-0.572329	0.368590	2.850898
1	-1.437562	-1.135117	3.280479
1	-1.891813	0.411881	4.048764
6	-4.491478	-0.249833	2.455467
1	-4.379671	-1.289739	2.805639
1	-5.313848	-0.230579	1.729518
1	-4.767556	0.365754	3.327407
6	-1.870545	-3.055941	-0.617931
1	-0.818018	-2.798184	-0.530169
6	-2.279873	-4.369984	-0.728163
1	-1.529074	-5.161514	-0.736995
6	-3.654839	-4.667893	-0.818825
6	-4.544794	-3.598573	-0.826199
1	-5.621083	-3.743558	-0.922809
6	-4.071254	-2.280216	-0.733848
6	-4.129277	-6.090467	-0.915528
1	-3.850544	-6.652736	-0.009185
1	-3.655895	-6.602514	-1.768677
1	-5.219705	-6.150011	-1.035844
6	-4.240398	0.462272	-3.021619
1	-3.635487	-0.338605	-3.471945
1	-3.799059	1.425154	-3.326344
1	-5.265599	0.408397	-3.424091
6	-5.415482	1.613413	-0.451476
1	-6.450272	1.385375	-0.755739
1	-5.144204	2.589142	-0.887295
1	-5.378538	1.711348	0.640281
6	0.262964	-0.493577	-1.984462
1	1.117979	-0.642248	-2.667758
6	2.028405	1.465661	-0.183121
6	2.346287	2.115381	-1.379284
6	1.698606	2.315694	0.875632
6	2.433622	3.499139	-1.513137
6	1.764869	3.708342	0.788635
6	2.153488	4.300808	-0.409146
6	1.980239	-0.774884	1.402212
6	1.071564	-1.652813	1.986147
6	3.099136	-0.480082	2.189860
6	1.231555	-2.189527	3.266381
6	3.302881	-0.987703	3.471518
6	2.351950	-1.851684	4.019975
6	3.054952	-1.013266	-0.944807
6	4.341486	-0.556716	-1.250217
6	2.814999	-2.354480	-1.263976
6	5.312146	-1.359151	-1.857197
6	3.748873	-3.190197	-1.875891
6	5.014914	-2.684715	-2.172662
9	1.268740	1.827030	2.050189
9	1.401141	4.477141	1.816634
9	2.173555	5.629689	-0.526462

9	2.717857	4.062882	-2.687262
9	2.562893	1.397079	-2.497174
9	4.714213	0.696458	-0.970809
9	1.619818	-2.912401	-0.975695
9	3.449001	-4.456134	-2.164806
9	5.927267	-3.459055	-2.751433
9	6.518604	-0.869912	-2.133987
9	4.041424	0.345395	1.709847
9	4.384813	-0.658579	4.174318
9	2.517555	-2.345361	5.243746
9	0.297972	-2.996223	3.777527
9	-0.053010	-2.022978	1.347411
5	1.917114	-0.169298	-0.111765

99

G

77	-2.441326	0.347690	-0.199747
8	0.993821	-0.415290	-0.467924
8	-0.967254	-1.271380	-1.046496
6	0.266432	-1.330463	-0.983596
7	-4.032868	-0.779950	-0.948274
6	-3.852026	-1.925259	-1.649950
6	-5.298875	-0.318937	-0.749749
6	-4.903559	-2.659140	-2.160588
1	-2.810677	-2.222960	-1.779793
6	-6.405542	-1.027064	-1.254599
6	-6.227128	-2.208401	-1.963746
1	-4.698768	-3.576710	-2.714491
1	-7.396272	-0.614706	-1.062222
7	-1.005038	1.699175	0.489654
6	-0.383790	1.566379	1.687553
6	-0.734434	2.797137	-0.264594
6	0.524475	2.484971	2.164518
1	-0.638453	0.670887	2.253844
6	0.214571	3.745822	0.153201
6	0.878094	3.591596	1.361838
1	1.000108	2.324893	3.130636
1	0.444473	4.560526	-0.532552
14	-2.265813	1.627281	-2.108041
14	-4.116759	1.642455	0.668537
8	-5.489467	0.795807	-0.076318
8	-1.364231	2.963744	-1.413188
6	-1.169347	0.884719	-3.440129
1	-1.062683	1.600228	-4.272594
1	-0.168489	0.638503	-3.065723
1	-1.633550	-0.033918	-3.834027
6	-3.778351	2.326322	-2.980607
1	-4.645172	2.490727	-2.328259
1	-3.522777	3.279257	-3.473132
1	-4.076034	1.614549	-3.768077
6	-4.334365	3.477347	0.319722
1	-5.358631	3.793022	0.578161
1	-3.640552	4.027268	0.977637
1	-4.120644	3.779554	-0.712055
6	-4.383386	1.485087	2.531738
1	-3.538043	1.966147	3.051958
1	-5.307325	2.000086	2.842135

1	-4.437291	0.443274	2.873051
1	-2.327021	-0.737799	1.447396
14	-2.757697	-2.035387	2.133605
6	-4.611440	-2.298539	1.925359
1	-5.205255	-1.392889	2.112554
1	-4.848478	-2.659850	0.914181
1	-4.940465	-3.070877	2.640531
6	-2.311457	-1.771642	3.942386
1	-2.788012	-0.862868	4.342210
1	-2.657351	-2.628542	4.543982
1	-1.225518	-1.688243	4.091725
6	-1.836894	-3.464759	1.349021
1	-2.166503	-4.409164	1.813194
1	-2.052521	-3.515189	0.271726
1	-0.750498	-3.387874	1.477490
1	0.780551	-2.223728	-1.384193
6	1.973207	4.524445	1.790478
1	1.727710	5.013689	2.747087
1	2.900721	3.951543	1.948324
1	2.165885	5.299912	1.036673
6	-7.392188	-2.986209	-2.508103
1	-7.403010	-4.008814	-2.097141
1	-8.350890	-2.506823	-2.267334
1	-7.317975	-3.081652	-3.603651
5	2.490100	-0.489719	-0.108776
6	3.264914	-1.645145	-0.972014
6	4.142208	-2.596363	-0.446056
6	3.159696	-1.637723	-2.366702
6	4.852491	-3.499164	-1.242998
6	3.841321	-2.523047	-3.201415
6	4.701248	-3.461865	-2.629050
6	3.181398	0.935012	-0.498263
6	2.596991	1.957080	-1.244385
6	4.495519	1.176132	-0.087317
6	3.224616	3.184956	-1.475464
6	5.167957	2.376424	-0.307464
6	4.516714	3.398690	-1.002135
6	2.385421	-0.764106	1.512722
6	1.762050	-1.941870	1.935309
6	2.643327	0.148773	2.535178
6	1.382089	-2.203743	3.248098
6	2.268243	-0.060969	3.866879
6	1.619792	-1.238367	4.227035
9	2.353080	-0.741579	-2.966469
9	3.687686	-2.477450	-4.524284
9	5.367864	-4.316395	-3.399856
9	5.673719	-4.391405	-0.692212
9	4.350815	-2.686755	0.872738
9	3.226163	1.333890	2.297404
9	2.474014	0.893191	4.778545
9	1.212446	-1.438596	5.478073
9	0.747405	-3.333629	3.570125
9	1.460467	-2.893975	1.029036
9	1.375371	1.824104	-1.781042
9	2.589409	4.155889	-2.138858
9	5.125255	4.564415	-1.210514

9	6.409621	2.562084	0.138931
9	5.147489	0.230410	0.605808

99

TSGH

7	2.712490	-1.977346	0.158468
6	2.475105	-2.837785	1.176956
6	2.239984	-2.283562	-1.082276
6	1.731955	-3.992674	1.019320
1	2.880599	-2.533056	2.143119
6	1.474437	-3.441660	-1.297372
6	1.190734	-4.305910	-0.245591
1	1.552944	-4.636832	1.881368
1	1.086777	-3.608750	-2.301529
7	4.589450	1.695397	0.729093
6	3.903750	2.809970	1.079390
6	5.929528	1.786258	0.514314
6	4.507160	4.044411	1.212540
1	2.838769	2.657841	1.257370
6	6.595532	3.019602	0.637983
6	5.894669	4.168421	0.985228
1	3.903950	4.909185	1.493496
1	7.669831	3.033534	0.452182
14	5.777752	-0.866882	0.171056
14	3.237295	0.112388	-1.775829
8	2.476681	-1.461234	-2.078249
8	6.596955	0.703970	0.176187
6	6.349715	-1.734619	1.741405
1	7.450124	-1.791163	1.784252
1	5.976590	-1.216844	2.637283
1	5.953073	-2.763847	1.757857
6	6.496189	-1.775560	-1.306978
1	6.660037	-1.100235	-2.157011
1	7.461495	-2.232702	-1.035746
1	5.817478	-2.580532	-1.632258
6	4.462560	0.306577	-3.190058
1	4.987103	-0.634381	-3.403864
1	3.932804	0.620750	-4.103882
1	5.216096	1.074599	-2.950972
6	1.848177	1.352047	-2.072217
1	2.258525	2.372643	-2.149647
1	1.315889	1.114467	-3.007296
1	1.118154	1.344316	-1.254236
6	6.579479	5.499534	1.122665
1	6.458792	5.895322	2.144322
1	7.654065	5.429547	0.904843
1	6.134378	6.239163	0.437343
6	0.337552	-5.525932	-0.459887
1	-0.481433	-5.320753	-1.164563
1	0.938069	-6.346322	-0.888351
1	-0.093957	-5.886667	0.484591
77	3.645095	-0.139135	0.463923
8	1.659220	0.737519	1.817396
14	-1.089263	0.653695	1.173185
6	-1.460782	0.662769	3.006104
1	-0.638912	1.165890	3.538070
1	-2.388930	1.222229	3.197952

1	-1.575654	-0.356187	3.400713
6	-0.324306	-0.900426	0.485418
1	-1.084611	-1.671452	0.318585
1	0.202055	-0.703220	-0.454510
1	0.398999	-1.278850	1.211791
6	2.251705	0.287109	2.835617
1	1.762305	0.408661	3.830420
6	-0.682423	2.343414	0.484931
1	-0.881279	2.398101	-0.593011
1	-1.298014	3.098215	0.995600
1	0.377462	2.561876	0.669676
1	-2.575996	0.485473	0.564280
5	-3.711670	0.162817	-0.213823
6	-4.046061	1.611360	-0.819656
6	-4.020967	2.741542	-0.000209
6	-4.384033	1.842227	-2.154735
6	-4.264473	4.033491	-0.460556
6	-4.647901	3.117448	-2.659750
6	-4.586373	4.220098	-1.806261
6	-3.103845	-0.944970	-1.215433
6	-2.038445	-0.618791	-2.063117
6	-3.465297	-2.297078	-1.213164
6	-1.329382	-1.548863	-2.817552
6	-2.776714	-3.267641	-1.948450
6	-1.705326	-2.890206	-2.754124
6	-4.688667	-0.333052	0.965775
6	-4.249728	-1.227195	1.945367
6	-6.005061	0.114100	1.112104
6	-5.025742	-1.637373	3.025336
6	-6.826045	-0.276979	2.174500
6	-6.331718	-1.155026	3.138703
9	-1.587989	0.646339	-2.108665
9	-0.279851	-1.183354	-3.549781
9	-1.023714	-3.813433	-3.432708
9	-3.122819	-4.552720	-1.875977
9	-4.490956	-2.738950	-0.478700
9	-4.473835	0.822726	-3.018116
9	-4.963049	3.289875	-3.941264
9	-4.831017	5.440395	-2.271657
9	-3.733385	2.607813	1.308757
9	-4.195692	5.077951	0.362786
9	-3.008076	-1.741915	1.863992
9	-4.541570	-2.479705	3.936171
9	-7.097690	-1.533997	4.155610
9	-8.073104	0.177110	2.270013
9	-6.549640	0.945621	0.215982
8	3.360356	-0.299020	2.792478

99

H

77	-0.490005	-1.487001	0.360924
8	-3.280339	0.148161	-1.430570
8	-1.308937	-0.826259	-1.777036
6	-2.318220	-0.296542	-2.197795
7	1.140161	-2.389103	-0.554282
6	1.467101	-2.139095	-1.844930
6	1.913201	-3.238897	0.176745

6	2.568479	-2.706121	-2.452174
1	0.814339	-1.442194	-2.366998
6	3.045507	-3.842719	-0.396344
6	3.402511	-3.569028	-1.711697
1	2.800303	-2.450653	-3.486969
1	3.651698	-4.485457	0.241794
7	-2.196724	-0.715725	1.301149
6	-2.359588	0.602279	1.550194
6	-3.198579	-1.570885	1.645741
6	-3.482317	1.120822	2.163208
1	-1.540718	1.244867	1.250958
6	-4.363909	-1.101213	2.278099
6	-4.523517	0.253803	2.550321
1	-3.537492	2.195807	2.340975
1	-5.127549	-1.835450	2.536516
14	-1.845044	-3.336926	0.187661
14	0.470569	-2.373379	2.269880
8	1.597582	-3.468071	1.433618
8	-3.077955	-2.854398	1.362877
6	-2.853520	-3.475720	-1.406382
1	-3.473189	-4.387034	-1.371875
1	-3.523917	-2.613653	-1.526438
1	-2.196490	-3.536189	-2.287811
6	-1.280349	-5.085414	0.578433
1	-0.449357	-5.143966	1.291020
1	-2.126186	-5.680818	0.960330
1	-0.948919	-5.545029	-0.367656
6	-0.554988	-3.425314	3.456861
1	0.106679	-4.134548	3.981611
1	-0.970510	-2.742377	4.216878
1	-1.391858	-3.979804	3.018954
6	1.591007	-1.418097	3.442340
1	0.990773	-0.837266	4.156752
1	2.176246	-2.164528	4.006005
1	2.286918	-0.740924	2.938087
1	0.621752	0.251098	0.327814
14	-4.893879	0.674579	-1.847506
6	-4.939878	0.947809	-3.698108
1	-4.787130	0.015184	-4.264100
1	-5.928112	1.347155	-3.980446
1	-4.184383	1.682655	-4.015110
6	-5.195820	2.230763	-0.872691
1	-6.258147	2.514455	-0.953851
1	-4.961492	2.067327	0.188966
1	-4.592098	3.074173	-1.232769
6	-5.979780	-0.736322	-1.280907
1	-5.785631	-1.652818	-1.860010
1	-5.788895	-0.956368	-0.218530
1	-7.045904	-0.480919	-1.394587
6	-5.762812	0.787174	3.212379
1	-5.509024	1.314671	4.145929
1	-6.267668	1.518545	2.559604
1	-6.476313	-0.013694	3.450589
6	4.672885	-4.100251	-2.311229
1	4.505138	-4.488294	-3.327810
1	5.408693	-3.282562	-2.388378

```
   1    5.114860   -4.895701   -1.695574
   1   -2.463662   -0.165429   -3.288387
   5    1.212247    1.320700   -0.055557
   6    2.581535    0.857552   -0.822113
   6    3.382600   -0.135070   -0.253971
   6    3.110566    1.426991   -1.985982
   6    4.565139   -0.609560   -0.818775
   6    4.272402    0.965309   -2.609758
   6    5.000776   -0.071166   -2.025570
   6    0.090823    2.093520   -0.964299
   6   -0.155692    1.776434   -2.306685
   6   -0.796854    3.035159   -0.431027
   6   -1.177705    2.345823   -3.065847
   6   -1.836629    3.629689   -1.151351
   6   -2.030589    3.279839   -2.482464
   6    1.654254    2.123597    1.296133
   6    2.621262    3.132279    1.250670
   6    1.161709    1.845313    2.570031
   6    3.110857    3.784172    2.385209
   6    1.624566    2.457548    3.734400
   6    2.613366    3.436397    3.641675
   9    3.021316   -0.701639    0.910533
   9    5.262266   -1.591252   -0.238115
   9    6.102372   -0.544300   -2.612283
   9    4.704691    1.520950   -3.742355
   9    2.518248    2.479194   -2.564256
   9    0.162760    0.959299    2.735300
   9    1.123800    2.119978    4.925304
   9    3.067807    4.041464    4.737129
   9    4.040253    4.734049    2.280038
   9    3.123314    3.529900    0.071664
   9   -0.730738    3.396966    0.862205
   9    0.560233    0.831420   -2.932181
   9   -1.404063    1.941490   -4.322777
   9   -3.057660    3.783279   -3.174496
   9   -2.672632    4.487942   -0.561550
99
TSHI
  77   -2.436218   -0.090415   -0.107174
   8   -0.525154    0.717086    0.864986
   8   -0.264714    0.906817   -1.353721
   6    0.206204    1.158393   -0.072203
   7   -1.610470   -1.961794   -0.439210
   6   -0.879799   -2.227833   -1.544616
   6   -1.791442   -2.939334    0.485062
   6   -0.312002   -3.463400   -1.779023
   1   -0.755018   -1.397063   -2.238211
   6   -1.206928   -4.204640    0.317153
   6   -0.448891   -4.484332   -0.815922
   1    0.248930   -3.630196   -2.697470
   1   -1.361429   -4.942025    1.105178
   7   -3.263301    1.807401    0.161522
   6   -2.706110    2.724475    0.989536
   6   -4.375753    2.148063   -0.546888
   6   -3.209213    4.002731    1.131216
   1   -1.817794    2.381486    1.521633
```

6	-4.927174	3.438107	-0.440315
6	-4.352255	4.386282	0.398088
1	-2.714817	4.700932	1.808296
1	-5.810970	3.658235	-1.039899
14	-4.270363	-0.395421	-1.448564
14	-3.268800	-1.093118	1.762607
8	-2.535999	-2.676622	1.540563
8	-4.927947	1.259374	-1.338975
6	-3.785095	-0.563815	-3.268486
1	-4.561501	-0.133458	-3.921785
1	-2.827681	-0.072524	-3.490512
1	-3.674688	-1.630933	-3.524527
6	-5.801289	-1.461907	-1.214111
1	-6.341877	-1.215546	-0.290936
1	-6.483926	-1.314530	-2.067081
1	-5.528658	-2.529485	-1.178365
6	-5.061907	-1.479814	2.151870
1	-5.682693	-0.571363	2.096435
1	-5.479145	-2.232717	1.470544
1	-5.124528	-1.870870	3.180971
6	-2.490147	-0.290564	3.271548
1	-3.096108	0.583058	3.564894
1	-2.458577	-0.995583	4.116740
1	-1.471332	0.054714	3.052021
6	-4.945374	5.759655	0.547470
1	-4.170760	6.508389	0.769391
1	-5.483193	6.068832	-0.360288
1	-5.665550	5.775587	1.383486
6	0.240361	-5.807519	-0.995429
1	-0.317331	-6.623838	-0.514038
1	0.380649	-6.047265	-2.059099
1	1.242092	-5.773421	-0.536409
1	0.655413	2.160197	0.029123
1	1.419363	0.496502	-0.029698
5	2.682692	0.053441	0.442477
6	3.586623	1.345897	0.118326
6	3.245739	2.614769	0.603099
6	4.706730	1.319810	-0.721257
6	3.917192	3.784523	0.257423
6	5.414302	2.467406	-1.091885
6	5.014895	3.709673	-0.601438
6	2.283158	-0.262644	1.961652
6	1.253730	-1.173091	2.224946
6	2.847794	0.333129	3.094012
6	0.788664	-1.474577	3.500234
6	2.399172	0.077578	4.393075
6	1.355866	-0.826093	4.597003
6	2.872969	-1.231132	-0.513255
6	3.201442	-2.511280	-0.057438
6	2.628287	-1.135865	-1.883533
6	3.223356	-3.631314	-0.891621
6	2.630641	-2.223965	-2.749765
6	2.917753	-3.490080	-2.242673
9	2.364033	0.067217	-2.425248
9	2.289501	-2.082874	-4.035125
9	2.843686	-4.560314	-3.033719

9	3.489519	-4.843287	-0.396809
9	3.506443	-2.725760	1.226786
9	0.684052	-1.842227	1.211421
9	-0.192593	-2.362161	3.677254
9	0.915753	-1.079229	5.826508
9	2.965934	0.680000	5.438206
9	3.880956	1.177987	2.982977
9	2.202896	2.757355	1.441839
9	3.519529	4.965731	0.729264
9	5.672654	4.811795	-0.946621
9	5.167662	0.164841	-1.218328
9	6.470079	2.382036	-1.898737
14	-0.150950	1.984186	-2.697115
6	-1.712728	3.026160	-2.665518
1	-2.612280	2.426631	-2.872095
1	-1.840230	3.501909	-1.680370
1	-1.654530	3.832600	-3.414968
6	-0.075308	0.912050	-4.227074
1	0.773608	0.216005	-4.188671
1	-1.000876	0.331824	-4.360283
1	0.040822	1.548817	-5.119786
6	1.362887	3.074020	-2.497944
1	1.534841	3.631250	-3.433804
1	1.241679	3.811902	-1.689299
1	2.262921	2.477953	-2.292063

99

I

77	1.950680	0.289103	0.034480
8	1.110387	-0.789151	-2.037515
8	-0.424278	-0.476607	-0.413817
6	-0.262531	-0.554563	-1.788519
7	2.787686	-1.602120	0.294645
6	2.037038	-2.723438	0.406401
6	4.144058	-1.713338	0.310708
6	2.594322	-3.971422	0.605342
1	0.963161	-2.577790	0.328452
6	4.762420	-2.953934	0.535837
6	3.995405	-4.103681	0.695720
1	1.937904	-4.838241	0.695299
1	5.852114	-2.979744	0.556877
7	1.335677	2.257340	-0.272366
6	0.845066	2.677760	-1.463699
6	1.564658	3.180050	0.703578
6	0.525808	3.993429	-1.720398
1	0.724648	1.903878	-2.220081
6	1.235752	4.532940	0.502925
6	0.705317	4.960682	-0.706714
1	0.119645	4.269435	-2.694237
1	1.406146	5.220402	1.331199
14	2.274829	1.059274	2.172864
14	4.100049	0.850143	-0.442128
8	4.884107	-0.639280	0.088697
8	2.120040	2.793554	1.830083
6	0.941604	0.818492	3.467580
1	1.171663	1.509952	4.296082
1	-0.057861	1.055055	3.087791

1	0.950670	-0.206417	3.862208
6	3.891768	0.766927	3.099354
1	4.756985	0.523857	2.470627
1	4.136820	1.647674	3.715976
1	3.725888	-0.078713	3.786983
6	5.058135	2.291566	0.284641
1	4.695480	3.213402	-0.199393
1	4.960433	2.421399	1.367659
1	6.126636	2.182517	0.034993
6	4.381090	0.994174	-2.304050
1	4.013651	1.979574	-2.635557
1	5.455568	0.934040	-2.543935
1	3.849085	0.229619	-2.881477
14	1.611855	-1.860260	-3.287979
6	3.281891	-2.577712	-2.843352
1	3.189538	-3.313569	-2.032218
1	3.678781	-3.101324	-3.729190
1	4.021861	-1.824246	-2.544862
6	1.692307	-0.842349	-4.862292
1	1.990246	-1.463766	-5.722784
1	0.707777	-0.402595	-5.091313
1	2.418570	-0.019098	-4.771737
6	0.349974	-3.243060	-3.437376
1	0.073980	-3.647838	-2.451303
1	-0.574018	-2.920592	-3.942109
1	0.781994	-4.063430	-4.034092
6	0.290766	6.386301	-0.927026
1	0.635768	7.040477	-0.114926
1	0.681168	6.769869	-1.882652
1	-0.807707	6.447226	-0.974841
6	4.627012	-5.444966	0.941226
1	4.333300	-5.833538	1.930245
1	4.286828	-6.180209	0.194495
1	5.723836	-5.393106	0.904301
1	-0.554331	0.385773	-2.285150
1	-0.876430	-1.359927	-2.219197
5	-1.848048	-0.232185	0.041428
6	-2.036806	-0.497750	1.652005
6	-1.187628	-1.249939	2.459591
6	-3.211723	-0.078264	2.291161
6	-1.453397	-1.560971	3.795295
6	-3.520829	-0.358640	3.621835
6	-2.627019	-1.108147	4.388429
6	-2.196423	1.327173	-0.401347
6	-2.648847	1.684729	-1.674631
6	-1.952158	2.411546	0.445833
6	-2.903210	2.997678	-2.074707
6	-2.208962	3.738886	0.102653
6	-2.692183	4.033624	-1.168978
6	-2.847633	-1.387602	-0.620410
6	-4.180664	-1.191053	-1.000652
6	-2.412327	-2.714348	-0.719687
6	-5.003468	-2.212355	-1.485905
6	-3.189469	-3.762205	-1.213152
6	-4.504832	-3.509436	-1.598335
9	-1.161561	-3.063516	-0.342741

9	-2.683449	-4.993611	-1.313202
9	-5.272132	-4.491138	-2.065575
9	-6.260812	-1.956402	-1.843808
9	-4.751370	0.016677	-0.926157
9	-2.844431	0.745817	-2.620696
9	-3.316262	3.270587	-3.313185
9	-2.893715	5.302489	-1.535108
9	-1.942273	4.732740	0.954217
9	-1.421383	2.227087	1.668405
9	-0.001650	-1.692405	2.005877
9	-0.556153	-2.241031	4.515825
9	-2.892705	-1.380325	5.663797
9	-4.121263	0.638308	1.615315
9	-4.655001	0.080992	4.165739

113

TSIJ

7	2.376944	-1.928501	-0.360962
6	2.275558	-2.779396	0.685641
6	1.716912	-2.223974	-1.512160
6	1.501567	-3.924296	0.643726
1	2.822523	-2.477697	1.580222
6	0.923917	-3.378445	-1.615732
6	0.786745	-4.238356	-0.531932
1	1.437262	-4.561618	1.526827
1	0.396855	-3.544832	-2.554209
7	4.440852	1.665510	-0.012249
6	3.835117	2.774704	0.474201
6	5.763137	1.709234	-0.326038
6	4.508922	3.966619	0.652145
1	2.782610	2.623544	0.728904
6	6.493459	2.903581	-0.173548
6	5.878356	4.048751	0.317057
1	3.975189	4.832613	1.047476
1	7.550049	2.886297	-0.441933
14	5.436133	-0.900453	-0.914131
14	2.613548	0.172053	-2.330287
8	1.799229	-1.389674	-2.524074
8	6.357838	0.622047	-0.761454
6	6.229315	-2.077949	0.337404
1	7.321406	-2.130360	0.195327
1	6.026607	-1.790295	1.376422
1	5.818026	-3.090197	0.183440
6	5.985181	-1.552074	-2.596083
1	6.012429	-0.758857	-3.354513
1	6.995546	-1.985079	-2.515282
1	5.304273	-2.343307	-2.949980
6	3.566446	0.378680	-3.936446
1	4.045222	-0.554191	-4.259805
1	2.867117	0.700899	-4.725241
1	4.343813	1.153180	-3.835505
6	1.235158	1.450733	-2.339789
1	1.646333	2.448861	-2.564066
1	0.481320	1.200097	-3.103101
1	0.748471	1.495462	-1.360277
6	6.635581	5.335469	0.494649
1	6.593262	5.671373	1.543558

1	7.691048	5.230618	0.207910
1	6.189303	6.136888	-0.116658
6	-0.095250	-5.453662	-0.619729
1	-0.971564	-5.266812	-1.257406
1	0.456998	-6.301070	-1.060743
1	-0.446174	-5.767881	0.373824
77	3.396512	-0.120159	-0.207568
8	1.690544	0.792931	1.023554
14	-1.370660	0.694858	0.957854
6	-1.496729	0.655603	2.825381
1	-0.593890	1.108083	3.264357
1	-2.366347	1.245476	3.153169
1	-1.601422	-0.370482	3.203543
6	-0.634213	-0.821384	0.172655
1	-1.362876	-1.639243	0.129979
1	-0.262691	-0.605816	-0.833630
1	0.215068	-1.131567	0.786481
6	1.976172	0.348585	2.249786
1	1.352366	-0.518609	2.583627
6	-1.050303	2.415236	0.310362
1	-1.264779	2.499474	-0.762512
1	-1.674276	3.134938	0.859858
1	0.011304	2.640392	0.487200
1	-2.910355	0.504866	0.537792
5	-4.119764	0.156310	-0.122676
6	-4.555215	1.606467	-0.654978
6	-4.468610	2.720708	0.182031
6	-5.043802	1.853617	-1.939546
6	-4.792216	4.014324	-0.220811
6	-5.392774	3.130680	-2.384425
6	-5.264722	4.217984	-1.518687
6	-3.587127	-0.912329	-1.204354
6	-2.637082	-0.532362	-2.159935
6	-3.896306	-2.277277	-1.187261
6	-1.987830	-1.422566	-3.010482
6	-3.260320	-3.208875	-2.013805
6	-2.303954	-2.778295	-2.930030
6	-4.945582	-0.388274	1.147023
6	-4.378541	-1.286309	2.054797
6	-6.249438	0.018809	1.445211
6	-5.020329	-1.735628	3.204975
6	-6.938266	-0.412887	2.583104
6	-6.318133	-1.292463	3.470012
9	-2.240011	0.749135	-2.229095
9	-1.047383	-1.004229	-3.853631
9	-1.670984	-3.664143	-3.698795
9	-3.546200	-4.508284	-1.924507
9	-4.815383	-2.769432	-0.350478
9	-5.202534	0.848503	-2.809841
9	-5.851282	3.319242	-3.619595
9	-5.588900	5.440006	-1.927595
9	-4.037656	2.569506	1.448924
9	-4.659021	5.044220	0.612877
9	-3.140710	-1.764716	1.829001
9	-4.416899	-2.578863	4.040951
9	-6.957446	-1.709762	4.557098

```
   9   -8.178888    0.004070    2.823154
   9   -6.911101    0.849209    0.630728
   8    3.363744   -0.164533    2.258574
   1    1.908708    1.131386    3.038673
  14    4.482623   -0.079627    3.533566
   6    3.742743    0.888406    4.966262
   1    4.462921    0.912755    5.801249
   1    3.523476    1.930628    4.684804
   1    2.813380    0.430676    5.341055
   6    6.024905    0.796725    2.922886
   1    6.464306    0.311133    2.040386
   1    5.790563    1.838426    2.653517
   1    6.791781    0.814049    3.714959
   6    4.843911   -1.841097    4.080601
   1    5.600154   -1.863017    4.882606
   1    3.927141   -2.315072    4.468350
   1    5.217930   -2.458531    3.249536
34
BPh3F
   5    0.000074    0.000165   -0.000251
   6   -1.391894    0.717694   -0.000151
   6   -1.616558    1.906303   -0.713154
   6   -2.490112    0.210826    0.713088
   6   -2.850154    2.554066   -0.732077
   9   -0.635781    2.452567   -1.435431
   6   -3.733401    0.839775    0.732610
   9   -2.365817   -0.905147    1.435010
   6   -3.912149    2.016665    0.000400
   9   -3.026203    3.668104   -1.433012
   9   -4.742640    0.336651    1.433808
   9   -5.088197    2.622780    0.000658
   6    0.074600   -1.564119   -0.000305
   6   -0.842225   -2.352650   -0.713868
   6    1.062067   -2.261992    0.713450
   6   -0.786660   -3.744865   -0.733008
   9   -1.805407   -1.775998   -1.436334
   6    1.138748   -3.653211    0.732863
   9    1.966015   -1.596518    1.436104
   6    0.209195   -4.396152   -0.000031
   9   -1.663154   -4.454085   -1.434510
   9    2.078466   -4.275991    1.434582
   9    0.271978   -5.717743    0.000137
   6    1.317567    0.846707   -0.000125
   6    2.459244    0.446379   -0.712864
   6    1.427897    2.051433    0.712610
   6    3.637152    1.190626   -0.732031
   9    2.441790   -0.676488   -1.434584
   6    2.594332    2.813514    0.731882
   9    0.399182    2.502142    1.434146
   6    3.702935    2.379305    0.000018
   9    4.689942    0.785578   -1.432707
   9    2.663348    3.939401    1.432595
   9    4.816047    3.094454    0.000140
3
co2
   6    0.000000    0.000000    0.000000
```

```
   8    0.000000    0.000000    1.163062
   8    0.000000    0.000000   -1.163062
14
HSiMe3
  14   -0.000002    0.000000   -0.384109
   1    0.000009   -0.000009   -1.882124
   6   -1.182765    1.339403    0.225343
   1   -1.207269    1.366477    1.327634
   1   -0.874707    2.336405   -0.129558
   1   -2.210060    1.157523   -0.130263
   6    1.751345    0.354600    0.225343
   1    1.787061    0.362090    1.327636
   1    2.460799   -0.410569   -0.129713
   1    2.107403    1.335294   -0.130094
   6   -0.568577   -1.694004    0.225343
   1   -1.585976   -1.925794   -0.129685
   1    0.102679   -2.492708   -0.130121
   1   -0.579928   -1.728696    1.327637
17
SF
   8    2.358047    0.406986   -0.000138
   6    1.950671   -0.727315   -0.000323
   1    2.616217   -1.618636   -0.000554
   8    0.671852   -1.090076   -0.000295
  14   -0.640394    0.055876    0.000080
   6   -0.524903    1.086403   -1.557771
   1    0.408866    1.668193   -1.561409
   1   -0.540778    0.447616   -2.455493
   1   -1.374264    1.786353   -1.624493
   6   -0.524753    1.084893    1.558921
   1   -0.539777    0.445155    2.455978
   1    0.408715    1.667178    1.562668
   1   -1.374423    1.784348    1.626831
   6   -2.147722   -1.052103   -0.000612
   1   -2.163768   -1.699116    0.890862
   1   -3.071684   -0.450659   -0.001307
   1   -2.162546   -1.699231   -0.892030
20
SF_CF3
   8   -0.623932   -1.672987   -0.000036
   6   -0.777869   -0.479827    0.000044
   8    0.164526    0.441742    0.000005
  14    1.878725    0.084109    0.000030
   6    2.252335   -0.875009   -1.560050
   1    1.715978   -1.835762   -1.558653
   1    1.949508   -0.307292   -2.454411
   1    3.332904   -1.079704   -1.638123
   6    2.252238   -0.875075    1.560088
   1    1.949077   -0.307575    2.454471
   1    1.716221   -1.836018    1.558477
   1    3.332868   -1.079417    1.638320
   6    2.639991    1.788961    0.000011
   1    2.333792    2.358100    0.891968
   1    3.740316    1.721897   -0.000735
   1    2.332632    2.358421   -0.891347
   6   -2.178853    0.175154    0.000037
```

```
   9   -2.330377    0.946165    1.085086
   9   -2.330890    0.944541   -1.086115
   9   -3.133984   -0.749089    0.000926
31
bisSA
   8   -0.668486    0.280466   -0.524550
   6    0.029989    1.487369   -0.363501
   1   -0.181559    1.914081    0.641348
   8    1.388938    1.282441   -0.520526
  14    2.225693   -0.093588    0.004397
   6    1.968403   -1.527335   -1.179855
   1    0.913234   -1.837915   -1.184247
   1    2.238942   -1.231388   -2.206474
   1    2.592779   -2.392161   -0.899211
   6    1.679349   -0.554790    1.746928
   1    1.732324    0.314537    2.422796
   1    0.644712   -0.929864    1.750873
   1    2.327688   -1.343637    2.163089
   6    4.022756    0.443512   -0.006950
   1    4.193268    1.278762    0.691064
   1    4.685394   -0.388000    0.284777
   1    4.321633    0.778247   -1.013403
   1   -0.295937    2.220583   -1.126529
  14   -2.219929   -0.103565    0.012183
   6   -2.378817    0.288887    1.846228
   1   -2.278236    1.368094    2.045792
   1   -3.367175   -0.024907    2.221348
   1   -1.611220   -0.237236    2.435945
   6   -2.382057   -1.940522   -0.310960
   1   -2.230141   -2.160741   -1.379703
   1   -1.630029   -2.506691    0.261586
   1   -3.379883   -2.310437   -0.023889
   6   -3.484145    0.882140   -0.969523
   1   -3.376580    0.691130   -2.049536
   1   -4.512937    0.613366   -0.677380
   1   -3.363440    1.965498   -0.803953
102
C'
77  2.498187   0.346227  -0.293235
8  -1.512188   1.368306  -2.427258
8  -1.514714   0.162465  -0.615624
6  -1.864438   0.327255  -1.779092
7  3.056775   -1.637563  -0.450448
6  3.146198   -2.424263  0.646661
6  3.281780   -2.186733  -1.672321
6  3.424217   -3.773822  0.576594
1  2.973204   -1.921136  1.599216
6  3.570465   -3.556206  -1.804580
6  3.628909   -4.375130  -0.683285
1  3.460773   -4.364289  1.492558
1  3.718819   -3.947626  -2.811269
7  1.863555   2.294396  0.142055
6  0.616665   2.708482  -0.202581
6  2.605527   3.097156  0.968489
6  0.038243   3.864545  0.275143
1  0.084699   2.039223  -0.869366
```

6	2.055496	4.297047	1.480971
6	0.764893	4.687688	1.164701
1	-0.985508	4.111454	-0.011759
1	2.678527	4.871316	2.166695
14	4.429757	1.091638	0.944386
14	2.927836	0.330136	-2.516162
8	3.212642	-1.405146	-2.728413
8	3.807823	2.748867	1.318827
6	4.809797	0.412272	2.678726
1	5.464847	1.090181	3.251720
1	3.888257	0.249664	3.257685
1	5.328366	-0.558103	2.585449
6	6.122268	1.509147	0.201382
1	6.750532	2.052062	0.927514
1	6.652206	0.583794	-0.081533
1	6.021892	2.127893	-0.702638
6	4.527127	1.137374	-3.100471
1	4.470186	2.231523	-2.977788
1	5.383310	0.777899	-2.512802
1	4.709765	0.917533	-4.165821
6	1.614506	0.720040	-3.814869
1	1.270560	1.756537	-3.679336
1	2.015249	0.620278	-4.837306
1	0.750286	0.051520	-3.696327
1	1.071691	0.017869	-1.108268
14	-2.044848	2.535163	-3.655717
6	-3.711269	3.120057	-3.056123
1	-4.435761	2.299911	-2.961426
1	-3.619521	3.603350	-2.071077
1	-4.113479	3.863015	-3.764841
6	-0.754575	3.872581	-3.553660
1	-1.073193	4.727826	-4.172637
1	-0.623682	4.226855	-2.520287
1	0.222155	3.531204	-3.925215
6	-2.048865	1.649081	-5.293833
1	-2.222548	2.380094	-6.100945
1	-1.072723	1.171793	-5.472064
1	-2.833063	0.881366	-5.352374
6	3.842000	-5.857600	-0.793853
1	2.906407	-6.380044	-0.534482
1	4.133076	-6.154877	-1.810915
1	4.614696	-6.204907	-0.090084
6	0.126092	5.900884	1.779062
1	-0.320159	6.548976	1.008011
1	-0.687341	5.593826	2.456656
1	0.847405	6.491120	2.360418
5	-1.903769	-0.550255	0.803645
6	-1.350997	0.581480	1.855531
6	-0.014561	0.616168	2.282327
6	-2.123138	1.652809	2.328045
6	0.525028	1.615020	3.090022
6	-1.614909	2.688906	3.116624
6	-0.277378	2.676660	3.495354
6	-1.075823	-1.950871	0.856416
6	-0.872410	-2.566824	2.097251
6	-0.516571	-2.627826	-0.230440

```
6  -0.199758   -3.774716  2.265491
6  0.141643   -3.854423  -0.112033
6  0.308322   -4.427787  1.142865
6  -3.536013   -0.732528  0.745162
6  -4.372123   0.172161  0.076842
6  -4.215139   -1.825623  1.298044
6  -5.745610   0.018390  -0.079386
6  -5.598021   -2.015373  1.185109
6  -6.371907   -1.091049  0.488110
9  -3.570011   -2.777135  1.977929
9  -6.174825   -3.079392  1.735715
9  -7.682086   -1.263101  0.362649
9  -6.453658   0.915684  -0.764607
9  -3.857440   1.278190  -0.511295
9  -1.336545   -1.979714  3.208882
9  -0.032830   -4.303542  3.476253
9  0.966552   -5.579210  1.274183
9  0.637887   -4.460275  -1.190708
9  -0.568872   -2.139451  -1.481951
9  0.863669   -0.334241  1.911631
9  1.810921   1.588101  3.434490
9  0.230112   3.672538  4.212928
9  -2.393906   3.711066  3.476265
9  -3.424421   1.763973  2.033535
6  -2.688418   -0.710095  -2.602030
9  -3.091479   -1.752588  -1.899295
9  -1.946115   -1.122953  -3.629565
9  -3.779650   -0.097080  -3.099185
99
H'
77  2.506052   -0.187799  0.061477
8  -1.168413   -2.759042  1.381807
8  -1.502943   -1.069212  0.011529
6  -1.825432   -1.724496  1.009935
7  2.452104   1.862012  0.358457
6  2.198991   2.718339  -0.658384
6  2.740443   2.369444  1.584905
6  2.232593   4.089680  -0.507775
1  1.968588   2.250790  -1.616418
6  2.796283   3.758108  1.796246
6  2.536349   4.641211  0.754374
1  2.003484   4.729740  -1.360407
1  3.022303   4.107858  2.803514
7  2.628993   -2.217714  -0.437200
6  1.699634   -3.105969  -0.002073
6  3.619796   -2.676574  -1.263634
6  1.701149   -4.441809  -0.344664
1  0.930818   -2.682572  0.635809
6  3.671657   -4.041502  -1.632769
6  2.719279   -4.942383  -1.185462
1  0.910899   -5.092338  0.031933
1  4.487711   -4.343661  -2.289862
14  4.428159   -0.097126  -1.386197
14  3.081078   -0.208036  2.250115
8  2.974893   1.530309  2.571943
8  4.526245   -1.864112  -1.726840
```

6	4.299940	0.634417	-3.135770
1	5.114038	0.279185	-3.790180
1	3.336698	0.387991	-3.609369
1	4.370620	1.735034	-3.079912
6	6.195046	0.275829	-0.812943
1	6.928607	0.096931	-1.616966
1	6.276176	1.332324	-0.505517
1	6.469717	-0.346647	0.051888
6	4.867045	-0.657130	2.651203
1	5.056075	-1.715690	2.406872
1	5.563033	-0.045858	2.059613
1	5.082445	-0.505399	3.722191
6	1.992339	-0.975917	3.589092
1	1.828607	-2.042707	3.361070
1	2.445254	-0.902081	4.591646
1	1.016368	-0.469000	3.591661
1	1.139464	-0.439041	1.008115
14	-1.578981	-3.719517	2.818854
6	-3.195271	-4.542142	2.369619
1	-3.951866	-3.811034	2.041777
1	-3.049996	-5.258885	1.545793
1	-3.608292	-5.092218	3.230785
6	-0.137372	-4.883447	2.970570
1	-0.229606	-5.481036	3.892015
1	-0.087707	-5.577361	2.118169
1	0.810961	-4.326212	3.017908
6	-1.701217	-2.498726	4.223185
1	-1.885455	-3.032440	5.170032
1	-0.760002	-1.936058	4.322600
1	-2.518881	-1.774545	4.084206
6	2.517771	6.128521	0.962168
1	1.488082	6.503838	0.847725
1	2.873242	6.404247	1.964492
1	3.140643	6.643104	0.213419
6	2.753026	-6.393866	-1.576390
1	2.816748	-7.038134	-0.683966
1	1.830608	-6.675319	-2.110762
1	3.609435	-6.620687	-2.226393
1	-2.701094	-1.425701	1.603710
5	-2.276391	0.257048	-0.451521
6	-2.034358	0.248453	-2.058870
6	-0.729159	0.265864	-2.560677
6	-3.038063	0.224961	-3.030548
6	-0.414703	0.241490	-3.916269
6	-2.770930	0.200748	-4.403906
6	-1.450564	0.208133	-4.851306
6	-1.531727	1.484082	0.333233
6	-1.350565	2.739024	-0.257766
6	-1.117359	1.407059	1.664258
6	-0.827251	3.838464	0.422275
6	-0.558723	2.467071	2.375652
6	-0.439380	3.706197	1.753672
6	-3.849939	0.106541	-0.007695
6	-4.533475	-1.114010	-0.057564
6	-4.632874	1.190096	0.407197
6	-5.858520	-1.288899	0.334521

6	-5.974653	1.070016	0.784302
6	-6.592668	-0.179272	0.753835
9	-4.131263	2.428801	0.449132
9	-6.664407	2.138520	1.171995
9	-7.860467	-0.313251	1.124184
9	-6.418297	-2.498302	0.314638
9	-3.897038	-2.229188	-0.480522
9	-1.732160	2.956209	-1.523879
9	-0.696205	5.017424	-0.188121
9	0.033174	4.753874	2.424362
9	-0.161713	2.305853	3.636368
9	-1.287815	0.269485	2.383729
9	0.311831	0.333634	-1.712430
9	0.855330	0.264722	-4.322645
9	-1.181198	0.186505	-6.151819
9	-3.768335	0.173662	-5.285479
9	-4.335233	0.232623	-2.692079

5. References

[S1] SAINT+, version 6.01: Area-Detector Integration Software, Bruker AXS, Madison, WI, 2001.

[S2] (a) Blessing R.H., An empirical correction for absorption anisotropy. *Acta Crystallogr*. **1995**, *A51*, 33-3. (b) SADABS, Area Detector Absorption Correction Program, Bruker AXS, Madison, WI, **1996**.

[S3] (a) Sheldrick, G.M. Phase annealing in SHELX-90: direct methods for larger structures. *Acta Crystallogr*. **1990**, *A46*, 467-473. (b) G. M. Sheldrick, A short history of SHELX *Acta Crystallogr***.** **2008**, *A64*, 112-122.

[S4] Sheldrick G.M., Acta *Crystallogr*. Crystal structure refinement with SHELXL. **2015**, *C71*, 3-8.

[S5] Dolomanov, O. V., Bourhis, L. J., Gildea, R. J, Howard, J.A.K., Puschmann, H. OLEX2: a complete structure solution, refinement and analysis program.J. *Appl. Cryst*. **2009**, *42*, 339-341.

[S6] Guzmán, J.; García-Orduña, P.; Polo, V.; Lahoz, F. J.; Oro, L. A.; Fernández-Alvarez, F. J. Ir-catalyzed selective reduction of CO_2 to the methoxy or formate level with $HSiMe(OSiMe_3)_2$. *Catal. Sci. Technol*. **2019**, *9*, 2858–2867.

[S7] Jiang, Y.; Blacque, O.; Fox, T.; Berke, H. Catalytic CO2 Activation Assisted by Rhenium Hydride/$B(C_6F_5)_3$ Frustrated Lewis Pairs—Metal Hydrides Functioning as FLP Bases. *J. Am. Chem. Soc*. **2013**, *135*, 7751–7760.

[S8] Ríos, P.; Curado, N.; López-Serrano, J.; Rodríguez. A. Selective reduction of carbon dioxide to bis(silyl)acetal catalyzed by a PBP-supported nickel complex. *Chem. Commun*. **2016**, *52*, 2114−2117.

[S9] Bibal, C.; Santini, C. C.; Chauvin, Y.; Vallée, C.; Olivier-Bourbigou, H. A selective synthesis of hydroxyborate anions as novel anchors for zirconocene catalysts. *Dalton Trans.* **2008**, 2866–2870.

[S10] Berkefeld, A.; Piers, W. E.; Parvez, M.; Castro, L.; Maron, L.; Eisenstein, O. Decamethylscandocinium-hydrido-(perfluorophenyl)borate: fixation and tandem tris(perfluorophenyl)borane catalysed deoxygenative hydrosilation of carbon dioxide. *Chem. Sci.*, **2013**, *4*, 2152–2162.

[S11] Agnew, D. W; Moore, C. E.; Rheingold, A. L.; Figueroa, J. S. Controlled cis Labilization of CO from Manganese(I) Mixed Carbonyl/Isocyanide Complexes: An Entry Point to Coordinatively Unsaturated Metallo-Lewis Acids. *Organometallics* **2017**, *36*, 363–371.

[S12] Parks, D. J.; Piers, W. E.; Parvez, M.; Atencio, R.; Zaworotko, M. J. Synthesis and Solution and Solid-State Structures of Tris(pentafluorophenyl)borane Adducts of PhC(O)X (X = H, Me, NPri2) *Organometallics* **1998**, *17*, 1369-1377.

[S13] Gaussian 09, Revision D.01, M. J. Frisch, G. W. Trucks, H. B. Schlegel, G. E. Scuseria, M. A. Robb, J. R. Cheeseman, G. Scalmani, V. Barone, G. A. Petersson, H. Nakatsuji, X. Li, M. Caricato, A. Marenich, J. Bloino, B. G. Janesko, R. Gomperts, B. Mennucci, H. P. Hratchian, J. V. Ortiz, A. F. Izmaylov, J. L. Sonnenberg, D. Williams-Young, F. Ding, F. Lipparini, F. Egidi, J. Goings, B. Peng, A. Petrone, T. Henderson, D. Ranasinghe, V. G. Zakrzewski, J. Gao, N. Rega, G. Zheng, W. Liang, M. Hada, M. Ehara, K. Toyota, R. Fukuda, J. Hasegawa, M. Ishida, T. Nakajima, Y. Honda, O. Kitao, H. Nakai, T. Vreven, K. Throssell, J. A. Montgomery, Jr., J. E. Peralta, F. Ogliaro, M. Bearpark, J. J. Heyd, E. Brothers, K. N. Kudin, V. N. Staroverov, T. Keith, R. Kobayashi, J. Normand, K. Raghavachari, A. Rendell, J. C. Burant, S. S. Iyengar, J. Tomasi, M. Cossi, J. M. Millam, M. Klene, C. Adamo, R. Cammi, J. W. Ochterski, R. L. Martin, K. Morokuma, O. Farkas, J. B. Foresman, and D. J. Fox, Gaussian, Inc., Wallingford CT, **2016**.

[S14] (a) A. D. Becke, *J. Chem. Phys.* **1993**, *98*, 1372−1377. (b) E. R. Johnson, A. D. Becke, *J. Chem. Phys.* **2006**, *124*, 174104 (c) C. Lee, W. Yang, R. G. Parr, *Phys. Rev. B* **1988**, *37*, 785−789. (d) S. Grimme, J. Antony, S. Ehrlich, H. Krieg, *J. Chem. Phys.* **2010**, *132*, 154104.

[S15] F. R. Weigend, R. Ahlrichs, *Phys. Chem. Chem. Phys.* **2005**, *7*, 3297−3305.

[S16] Y. Zhao, D. G. Truhlar, *J. Chem. Phys.* **2006**, *125*, 194101.

[S17] A. V. Marenich, C. J. Cramer, D. G. Truhlar, *J. Phys. Chem. B* **2009**, *113*, 6378−6396.

[S18] V. S. Bryantsev, M. S. Diallo, W. A. Goddard, III, *J. Phys. Chem. B* **2008**, *112*, 9709−9719.

[S19] S. Grimme, *Chem. Eur. J.* **2012**, *18*, 9955-9964

[S20] G. Luchini, J. V. Alegre-Requena, I. Funes-Ardoiz, R. S. Paton, F1000Research, 2020, 9, 291. GoodVibes version 3.1.1 DOI: 10.12688/f1000research.22758.1

[S21] R. Tanaka, M. Yamashita, L. W. Chung, K. Morokuma, K., K. Nozaki. *Organometallics*, **2011**, *30*, 6742-6750

[S22] C.Y. Legault; CYLview, 2.0, Université de Sherbrooke, 2020 (http://www.cylview.org).
